# Always Look on Both Sides: Phylogenetic Information Conveyed by Simple Sequence Repeat Allele Sequences

**DOI:** 10.1371/journal.pone.0040699

**Published:** 2012-07-13

**Authors:** Stéphanie Barthe, Felix Gugerli, Noelle A. Barkley, Laurent Maggia, Céline Cardi, Ivan Scotti

**Affiliations:** 1 Unité Mixte de Recherche “Ecologie des forêts de Guyane”, University of French West Indies and French Guiana, Kourou, French Guiana; 2 Biodiversity and Conservation Biology Research Unit, Swiss Federal Research Institute for Forest, Snow and Landscape Research, Birmensdorf, Switzerland; 3 Plant Genetic Resources Conservation Unit, United States Department of Agriculture – Agricultural Research Service, Griffin, Georgia, United States of America; 4 Unité Mixte de Recherche “Amélioration génétique et adaptation des plantes méditerranéennes et tropicales”, Institut Agronomique néo-Calédonien, Nouméa, New Caledonia; 5 Unité Mixte de Recherche “Amélioration génétique et adaptation des plantes méditerranéennes et tropicales”, Centre de coopération internationale en recherche agronomique pour le développement, Montpellier, France; 6 Unité Mixte de Recherche Ecologie des forêts de Guyane, Institut National de la Recherche Agronomique, Kourou, French Guiana; Michigan State University, United States of America

## Abstract

Simple sequence repeat (SSR) markers are widely used tools for inferences about genetic diversity, phylogeography and spatial genetic structure. Their applications assume that variation among alleles is essentially caused by an expansion or contraction of the number of repeats and that, accessorily, mutations in the target sequences follow the stepwise mutation model (SMM). Generally speaking, PCR amplicon sizes are used as direct indicators of the number of SSR repeats composing an allele with the data analysis either ignoring the extent of allele size differences or assuming that there is a direct correlation between differences in amplicon size and evolutionary distance. However, without precisely knowing the kind and distribution of polymorphism within an allele (SSR and the associated flanking region (FR) sequences), it is hard to say what kind of evolutionary message is conveyed by such a synthetic descriptor of polymorphism as DNA amplicon size. In this study, we sequenced several SSR alleles in multiple populations of three divergent tree genera and disentangled the types of polymorphisms contained in each portion of the DNA amplicon containing an SSR. The patterns of diversity provided by amplicon size variation, SSR variation itself, insertions/deletions (indels), and single nucleotide polymorphisms (SNPs) observed in the FRs were compared. Amplicon size variation largely reflected SSR repeat number. The amount of variation was as large in FRs as in the SSR itself. The former contributed significantly to the phylogenetic information and sometimes was the main source of differentiation among individuals and populations contained by FR and SSR regions of SSR markers. The presence of mutations occurring at different rates within a marker’s sequence offers the opportunity to analyse evolutionary events occurring on various timescales, but at the same time calls for caution in the interpretation of SSR marker data when the distribution of within-locus polymorphism is not known.

## Introduction

Simple sequence repeats (SSRs; see glossary, Table 1) can conveniently be used as genetic markers owing to their polymorphism and relative ease of interpretation [1]. SSRs are widely used for, e.g., reconstructing phylogenetic relationships [2], for analysing spatial genetic structure among and within populations [1], and for detecting and explaining patterns linked to habitat fragmentation and gene flow [3]. In many studies, SSR markers demonstrated recent expansions or bottlenecks in various plant [4], [5], animal [6]–[8] and human populations [9], [10].

**Table 1:** Glossary.

**Amplicon**	Product of a DNA amplification reaction.
**SSR**	Simple sequences repeat (or “microsatellite”). Tandem repeat of simple di- to hexa-nucleotide sequence motifs.
**FR**	Flanking region. DNA sequences appearing in an amplicon on either side of the SSR sequence.
**Indel**	A sequence gap in a DNA sequence alignment caused by an insertion or deletion mutation.
**SNP**	Single nucleotide polymorphism. DNA polymorphism that involves a change in a single base of a DNA sequence.
**SSR locus**	A specific genomic region consisting of SSR (microsatellite) DNA and its flanking regions (FRs).

SSRs are often expected to mutate following the stepwise mutation model (SMM) [11], whereby mutations alter the length of the repeat either by adding or by deleting a single repeat unit at a fixed rate [12]. More elaborate mutational processes, such as the two-phase model (TPM; [13]) and the generalized stepwise model (GSM; [14], [15]), allow for multi-step mutations. Departures from these models can however occur [16]–[20]. Moreover, SSR alleles are generally scored as the length (in base pairs) of PCR amplicons (see glossary, Table 1) containing the SSR, with differences in amplicon size taken to represent differences in repeat number in the SSR. Amplicon size includes of course SSR repeat number plus the length of the flanking region (FR; see glossary, Table 1). These chunks of sequence can be and actually often are polymorphic and may contain both single nucleotide polymorphisms (SNPs; see glossary, Table 1) and insertions/deletions (indels; see glossary, Table 1) [18], [21]–[24]. Indels which have been demonstrated to occur in the FR sequences of some SSR alleles clearly contribute to total amplicon size, but they are interpreted under the SMM as (false) variation in SSR length. SNPs contribute to the total sequence variance of SSR loci (see glossary, Table 1) that goes largely unnoticed when only amplicon size is recorded (which is typical of most SSR studies). As a consequence, SSR amplicon size data are prone to a particular form of size homoplasy [15], i.e. equally sized alleles may have different sequences and therefore be evolutionarily different. This casts doubt on the evolutionary and population-genetic inferences that can be drawn from estimates of divergence obtained from SSR amplicon size data. It is therefore necessary to examine the kinds of evolutionary information conveyed in SSR alleles, to assess their consequences on fragment size-based evolutionary inferences, and to find ways to take advantage of this so far unexploited source of variability.

**Figure 1:**
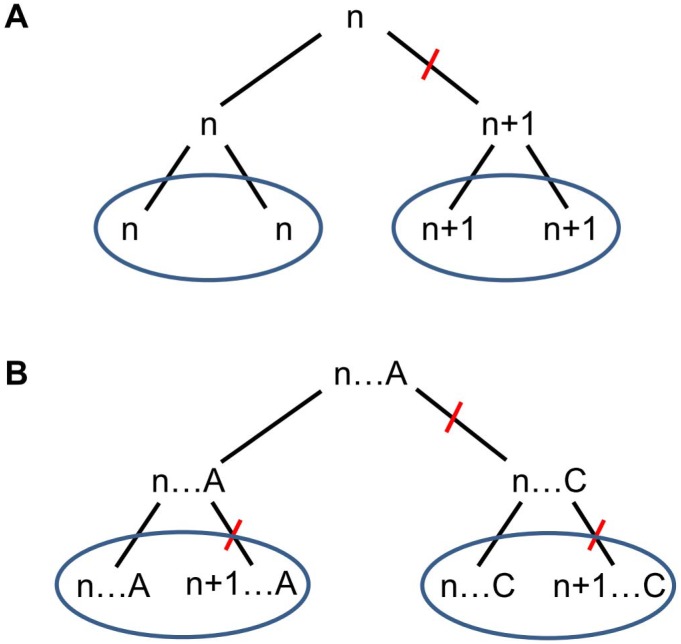
Two alternative genealogies for simple sequence repeat (SSR) alleles containing the same number of repeats. SSR alleles are indicated by their number of repeats (n, n+1). The A/C letters indicate a SNP in the flanking regions. Red bars correspond to mutational events in the flanking region sequence or in the number of SSR repeats. (A) No sequence information: deduced genealogy of observed data (third line) groups alleles together according to their number of repeats and involves a single SSR mutation. Genealogy is recent. (B) Consideration of sequence information: deduced genealogy involves a SNP mutation and two SSR mutations (alternative topology will involve two identical and independent substitutions at the same nucleotide site and a unique SSR mutation, which is less likely). Genealogy is ancient and SSR alleles do not group according to their numbers of repeats.

Figure 1 succinctly shows how information in FR sequence variation may change one’s view of SSR marker evolutionary information content. Figure 1A shows a “plain” SSR locus, where a mutation (bar on the right branch) changes the number of repeats from n to n+1. The four observed alleles are then linked by a simple genealogy where alleles with the same number of repeats are evolutionarily closer. This is the classical view of SSR allele evolution under repeat-number mutational models. Figure 1B shows four alleles with the same numbers of repeats as in Figure 1A, but in this case information from a SNP in the FR (indicated by the “…A/…C” symbols) is added. Here, alleles with different numbers of repeats share the same SNP. Given that SNP mutation rates are generally lower than those of SSRs, the most-likely genealogy is one in which divergence occurs first at the SNP (mutation in the upper right branch) and then in SSRs within flanking-region haplotypes (mutations in the lower branches). In this case, three mutations have occurred, the genealogy is expected to be older than in 1A, and the alleles are grouped based on their FR sequences and not based on the number of repeats. A simulation study [25] revealed that SSR and SNP sources of variation provide independent evolutionary information, so it is reasonable to study the role of SNP variation in SSR flanking regions. We therefore asked the following questions: How much does amplicon size variation reflect variation in SSR repeat number? How important is the contribution of the various sources of sequence variation in total SSR diversity? Do these extra-SSR polymorphisms carry a different population and phylogenetic differentiation signal than the SSRs themselves? If so, how do the different signals combine? How does one best interpret SSR data, once these departures from the SMM have been acknowledged and assessed? To answer these questions, we sequenced SSR amplicons obtained from three divergent genera of long-lived angiosperm tree species to inspect the distribution of polymorphisms within and around SSRs and to evaluate the impact of each source of sequence variation on the detection of evolutionary and population genetic signals.

## Materials and Methods

### Choice of Data Sets

Three data sets were utilized in this study. The structures of the three data sets analysed here varied in the following ways: the *Citrus* data represent sequences of individual trees each belonging to a different species, variety or cultivar; the *Jacaranda* data were collected in four different sampling sites considered as four distinct populations; and the oak data came from a single mixed stand of sessile/pedunculate oaks (*Quercus robur, Q. petraea*). The Citrus, Jacaranda, and Quercus data sets were submitted to partially different types of analyses according to the structure of each data set.

**Table 2:** Characteristics of simple sequence repeat (SSR) data sets of the three taxa, *Citrus* (C), *Jacaranda* (J) and *Quercus* (Q).

Data set	Type of data	SSR locus	Size (bp) a	N b	Reference
Set C	Collection ofprovenances	cAGG9	123	34	[30]
		CCT01	167	34	[27]
		GT03	177	36	[27]
Set J	Fourpopulations	Jc3A10	180	100	This study
		Jc3F4	144	101	This study
		Jc3H10	215	96	This study
Set Q	One mixedpopulation	QrZAG30	255	47	[31]

a Total length of the amplicon consensus sequence in base pairs.

b Total number of analysed alleles.

Given the taxonomy and prevailing theory on many *Citrus* species being derived by natural hybridization from ancestral species, alleles were chosen to minimize known hybrids from the data set of Barkley et al. [26], [27], who examined the genetic diversity of 370 *Citrus* accessions. The individual identity of each accession (as provided by the University of California, Riverside citrus database, http://www.citrusvariety.ucr.edu/) is given in the Supplementary Table 1 of Barkley et al. [26]. The *Jacaranda* data were obtained from three sites in French Guiana (Counami, Paracou and Saint-Laurent) and one in Brazil (Tapajos) (Table S1). The *Quercus* alleles were taken from genetic analyses of *Q. petraea* and *Q. robur* in a mixed oak stand in Switzerland [28], [29]. The SSR markers used for this study were: cAGG9, CCT01 and GT03 for *Citrus* [26], [30]; Jc3H10, Jc3F4 and Jc3H10 for *Jacaranda*; QrZAG30 for *Quercus* [31]. Allele sequencing for the *Citrus* and *Quercus* data sets was as reported in Barkley et al. [26] and Gugerli et al. [29].

### Molecular Methods

*Jacaranda* markers were obtained following the method developed for SSR markers in tropical crops by Billotte et al. [32]. Ten nanograms of DNA was digested with *Rsa*I, followed by an enrichment step in (GT)_n_ and (GA)_n_ repeats by hybridization of cleaved fragments with biotin-labelled (CA)_8_ and (CT)_8_ oligoprobes and capture of the selected sequences with streptavidincoated magnetic beads. Those fragments were ligated into a pGEM-TEasy vector (Promega, Madison, WI), and plasmids were then used to transform competent XL-1 Blue strain *Escherichia coli* (Stratagene, La Jolla, CA). Positive colonies were tested using colony polymerase chain reaction (PCR) to check the presence of inserts. Amplified fragments were then transferred from an agarose gel onto a N+ Hybond membrane for SSR presence screening by hybridization with labeled γ^−32^ P (GT)_15_ and (GA)_15_ oligoprobes. One hundred and thirteen bacterial clones containing plasmids with inserts that gave strong hybridization signals, with sizes ranging from 500 to 1000bp, were selected and cultured. Plasmids were extracted and sequenced using the universal T7 primer on an automated ABI 377 capillary sequencer (Applied Biosystems, Foster City, CA). After discarding duplicates, hybrid clones and clones with the SSR region too close to either end of the sequence, thirteen sequences were suitable for primer design and allowed a successful design using Oligo 3 software (Molecular Biology Insights Inc, USA). Upon screening for polymorphism and clarity of PCR patterns, three primer pairs were chosen for subsequent analyses. Primer sequences, annealing temperatures and GenBank/EMBL accession numbers are shown in Table S2. Dried cambium discs and leaf samples were flash-frozen in liquid nitrogen and later ground to a powder using a mortar and pestle. DNA extractions were performed by following Colpaert et al. [33]. PCRs for the detection of SSR polymorphisms were carried out in a 12 µL volume containing 6 µL 20-fold diluted DNA, 1× *Taq* buffer, 0.26 mM of dNTP, 0.03 U/µL *Taq* DNA polymerase (all products from Invitrogen, Carlsbad, CA) and 0.54 µM of each primer (MWG Biotech, Ebersberg, Germany). For Jc3A10 primers, 0.3% BSA was added. An initial denaturation at 94°C for 5 min was followed by 35 cycles of 94°C for 30 s, annealing temperature for 30 s and 72°C for 30 s, and a final extension at 72°C for 10 min. Genotyping was performed using fluorescently labeled primers (PET, 6FAM and NED) in the previous PCR protocol and fragments were separated on an ABI 3130XL capillary sequencer (Applied Biosystems, Foster City, CA) using ABI POP4 and Applied Biosystems LIZ-500 as internal standard, following the manufacturer’s instructions. DNA was bidirectionally sequenced directly from PCR amplification products. When necessary, gametic phase was determined in heterozygotes by cloning amplicons and sequencing at least one allele per individual following the TA cloning kit protocol from Invitrogen (Carlsbad, CA). PCR products were ligated with the plasmid pCR®2.1 and used to transform competent DH5 cells. After an overnight incubation at 37°C, white colonies were isolated. The presence of an SSR allele was checked by PCR with universal M13 primers. PCRs consisted of 1 µL of cultivated colonies, 1× *Taq* buffer, 0.125 mM dNTPs, 0.025 U/µL *Taq* DNA polymerase (all products from Invitrogen, Carlsbad, CA) and 0.83 µM forward and reverse primers. Cycling conditions consisted of 94°C for 5 min; 35 cycles of: 94°C for 1 min, 50°C for 1 min, 72°C for 2 min; and one cycle of 72°C for 5 min. PCR products were checked on a 4% agarose gel in 0.5× TAE. Plasmids were sequenced with the ABI BigDye® Terminator V3.1 kit (Applied Biosystems, Foster City, CA) following the manufacturer’s protocol. Cloning products were diluted 1∶40, purified with ExoSap-IT (USB Corporation, Cleveland, OH), and separated on an ABI 3130XL capillary sequencer (Applied Biosystems, Foster City, CA). All sequences were aligned and edited using CodonCode Aligner V1.6.3 (Codoncode Corporation, Dedham, MA).

### Data Analyses

For each data set (*Citrus, Jacaranda,* and *Quercus*), each allele was characterised by the following information: (a) amplicon size variation, (b) SSR variation, (c) FR sequence variation and (d) amplicon sequence variation. Each of these portions of information conveyed by SSR marker data was analysed as a separate source of variation. Results were compared across data sets to assess their evolutionary information content. Indels were coded as SNP mutations to represent them as single mutational events.

Levels of polymorphism (number of alleles (*A*) or haplotypes (*h*), Nei’s genetic diversity (*H_e_*), number of SNPs and indels) were recorded for each source of variation. Linkage disequilibrium (LD) was computed for FR sequence variation.

**Table 3:** Polymorphism at each simple sequence repeat (SSR) locus differentiated by segments of the DNA amplicon. SSR1, SSR2 and SSR3: respectively first, second and third SSR occurring in each amplicon (see figure S1 for details on each amplicon’s sequence); FR: flanking regions; *H_e_*: Nei’s genetic diversity; A: number of alleles; h: number of haplotypes. Data sets: C, *Citrus*; J, *Jacaranda*; Q, *Quercus*.

	Amplicon size variation		Amplicon sequence variation		SSR1 variation			SSR2 variation			SSR3 variation			FR sequence variation					
SSR locus(data set)	*He*	*A*	*He*	*h*	Repeat unit	*He*	*A*	Repeat unit	*He*	*A*	Repeat unit	*He*	*A*	Sequence length (bp)	Numberof SNPs	SNPs per 100 bp	Numberof indels	*He*	*h*
cAGG9 (C)	0.772	4	0.868	11	(GAG)_n_	0.772	4	(GAA)_n_	0.485	4	–	–	–	82	5	6.10	1	0.542	8
CCT01 (C)	0.758	4	0.966	20	(TCC)_n_	–	–	–	–	–	–	–	–	140	17	12.14	0	0.861	14
GT03 (C)	0.783	5	0.900	12	(GT)_n_	–	–	(AT)_n_	–	–	–	–	–	138	12	8.70	1	0.808	9
Jc3A10 (J)	0.856	13	0.937	43	(CT)_n_	0.769	12	(CA)_n_	0.700	6	–	–	–	116	2	1.72	1	0.306	5
Jc3F4 (J)	0.912	20	0.920	32	(GA)_n_	0.853	15	–	–	–	–	–	–	104	3	2.88	4	0.662	11
Jc3H10 (J)	0.856	13	0.922	40	(CT)_n_	0.748	19	(CACG)_n_	0.476	3	(CGCACA)_n_	0.527	4	111	1	0.90	2	0.409	2
QrZAG30 (Q)	0.966	23	0.983	31	(GA)_n_	0.915	16	–	–	–	–	–	–	185	14	7.57	15	0.922	18

Data were analysed in two ways: (a) for all data sets, at the level of individual alleles; (b) for *Jacaranda* only, at the population level.

- At the individual-allele level, matrices of pairwise genetic distances were computed between individuals and between alleles based on each source of variation. For amplicon length, distance is represented by difference in length; for SSR length, distance is the difference in number of repeat units; for whole amplicon sequence, distance is the total number of differences, including SNPs, indels and number of repeat units; for flanking region sequences, distance is the total number of SNPs and indels. The correlation between matrices was tested by a Mantel test. LD between all pairs of polymorphic sites in FR sequences was tested with a Markov chain Monte Carlo (MCMC) procedure of 10,000 steps and a burn-in phase of 1,000 steps (default values). Pairwise genetic distances for amplicon and FR sequence variation were computed based on the number of mutational steps between sequences. Pairwise genetic distances for SSR variation and amplicon size variation data were computed as Euclidian distances (genetic distances based on the SSR variation were not computed for *Citrus* loci CCT01 and GT03, which displayed extremely complex repeat sequences; see Figure S1). Most of the above calculations were performed with ARLEQUIN versions 3.11 and 3.5.1.2 [34]. Genetic distance matrices for SSR variation and amplicon size variation data were computed with an ad-hoc routine in R [35], and correlations between genetic distance matrices were computed with the mantel.test function of the R package “ape”.
- At the population level, for *Jacaranda,* global and pairwise F_ST_ values [36] were computed for all sources of variation; R_ST_ values [37] were computed on sources of variability comprising amplicon size variation and SSR variation; N_ST_ values [38] were computed on amplicon and FR sequence variations. In N_ST_ calculations on amplicon sequence variation, mutations in repeat number were weighted less than substitutions (weight  = 0.05) to account for their faster mutation rate. Weight choice was based on the observation that SSRs have, in our *Jacaranda* sample, approximately ten times more alleles than each SNP (see Results section). Considering that rare alleles might be missing from our sample, we estimated a ratio of 1∶20 in allele richness between SNPs and SSRs and used this ratio to establish the relative weight. A hierarchical analysis of molecular variance (AMOVA) was performed to partition genetic variance into within- and among-population components. The significance of variance components was tested by 1,000 permutations [39]. These analyses were performed with ARLEQUIN 3.11 and 3.5.3.1. Locus-by-locus pairwise F_ST_, R_ST_, and N_ST_ matrices were used to construct population-level consensus UPGMA (Unweighted Pair Group Method with Arithmetic Mean) cladograms by averaging genetic distance information of three loci with SplitsTree4 v4.6 [40], [41].

## Results

### Distribution of Polymorphism

In order to evaluate homoplasy and the variation in phylogenetic signal from different regions of SSR alleles (repeat region and FR), sequence data produced from three divergent tree genera were evaluated. Forty-six, 101, and 47 samples constituted the *Citrus, Jacaranda,* and *Quercus* data sets, respectively. Three, three and one SSRs were genotyped and sequenced for the *Citrus*, *Jacaranda* and *Quercus* data sets, respectively. The lengths of the sequenced fragments varied between 123 and 255 bp (Table 2). Whenever possible, multiple alleles of the same fragment size were genotyped and sequenced to estimate the degree of sequence divergence among equally sized alleles (“size homoplasy”). This includes both variation in SSR repeat number (that is taken into account by the SMM) and variation in FR sequences, which is the focus of our study.

**Figure 2:**
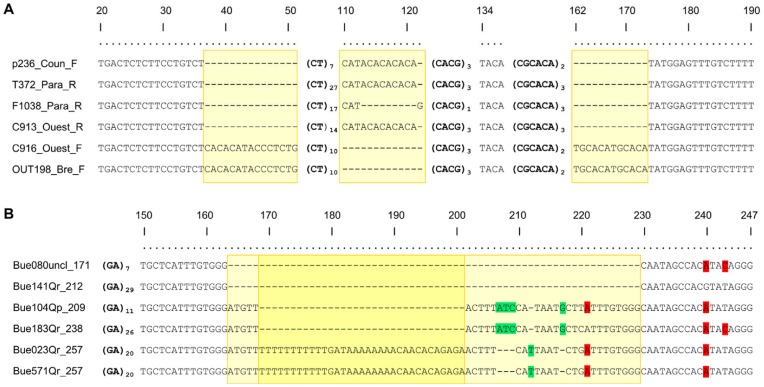
Alignment of a subset of DNA fragments at simple sequence repeat (SSR) loci Jc3H10 (*Jacaranda copaia*) (A) and QrZAG30 (*Quercus robur*) (B). Numbers in the top line indicate positions relative to the consensus sequence of (A) 190 bp and (B) 247 bp. Bold nucleotides in brackets indicate SSR motifs and their number of repeats. Dashes indicate gaps, highlighted nucleotides in green (light grey*) indicate indels of one to three bases, highlighted nucleotides in red (dark grey*) mark mutations from one base to another, and yellow (light grey*) boxes indicate groups of insertion/deletions longer than three bases and considered as a single mutational event. * In shades-of-gray printouts.

Levels of polymorphism (number of alleles/haplotypes, Nei’s genetic diversity, number of SNPs and indels) for each amplicon and each source of variation are displayed in Table 3. Large amounts of variability were observed, as expected, in the amplicon sequences. SNPs were found to interrupt the repeat in all three taxa (imperfect SSRs: 33 sequences, data not shown). Sequences with SNPs within the repeat were excluded from subsequent analyses owing to the complexity of the mutation model of SSR repeats containing SNPs. Four loci had two or more repeats (compound SSRs). The highest number of alleles and Nei’s genetic diversity were detected for amplicon sequence variation, then for amplicon size variation and lowest was found for SSR variation (Table 3). FR sequence variation showed the lowest diversity. Indels were observed in six loci out of seven, while SNP density varied between 0.90 and 12.14 SNPs per 100 bp.

**Figure 3:**
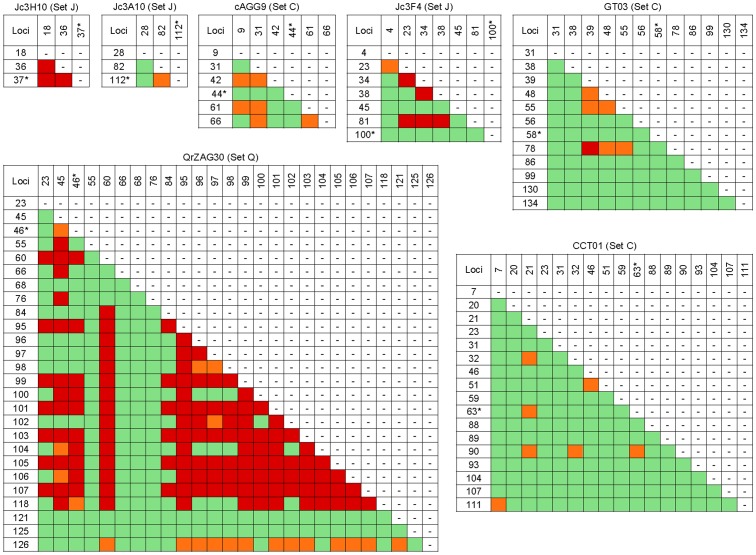
Linkage Disequilibrium (LD) for pairs of polymorphisms in the flanking regions. Dark cells contain LD values significantly different from zero. An asterisk next to a locus name indicates that the SSR repeat(s) is (are) located between that locus and the next. Colouring (shading) indicates the degree of significance of the test: green (light grey*), *P*>0.05; orange (grey*), 0.05<*P*>0.001; red (dark grey*), *P*<0.001. * In shades-of-gray printouts.

An example of the complexity of the polymorphisms observed is shown for markers Jc3H10 and QrZAG30 (*Jacaranda* and *Quercus* data set, Figure 2; the complete alignments for all sequenced amplicons are given in Figure S1). Three indels of several nucleotides (between 12 and 15 bases) were detected in the FR sequences of the compound marker Jc3H10. One was upstream of the first SSR, the second was between the repeat motifs and the last was detected after the SSR repeats. In the oak marker QrZAG30, in addition to SNPs, a large 66-nucleotide indel was observed, with SNPs occurring among alleles carrying the DNA fragment involved in the indel. Several cases of size homoplasy were detected, such as: (i) indels in the FRs compensating differences in number of repeats, (ii) compound SSRs with the same number of repeats, but composed of different numbers of repeats in different motifs, (iii) SSRs with the same amplicon size and number of repeats but with SNPs in the FR sequences or interrupting the repeat (Figure S1). For the three data sets, 50 alleles with the same amplicon sizes out of 68 (74%) showed size homoplasy (Table S3).

### Linkage Disequilibrium

LD was tested for all pairs of SNP and indel sites within the FR sequence variation of each marker (Figure 3). For the *Citrus* data set, only 19 pairs out of 217 (8.8%) showed significant LD. For the three SSR loci, LD was very irregular and there was no relationship between LD and sequence distance (Mantel tests, *P*>0.05 for all loci). For the *Jacaranda* data set, ten pairs out of 27 (37%) showed significant LD. For marker Jc3F4, the SNP at position 81 was in disequilibrium with two indels and one SNP, all located upstream of the SSR. LD was strongest for Jc3H10, where all three pairs were significant. The relationship between LD and sequence distance was significant (Mantel test, *P*  = 0.023). Tests of correlation between LD and distance in bp could not be performed for Jc3A10 and Jc3H10 because only three nucleotide sites were polymorphic (three LD values). For the *Quercus* data set (marker QrZAG30), 146 pairs of SNPs and indels out of 325 (44.9%) showed significant LD. In particular, 13 or 14 contiguous SNPs and indels located downstream of the SSR at positions 84–107 formed a disequilibrium block, itself in weaker disequilibrium with upstream polymorphic sites at positions 23, 45 and 46 and another SNP downstream the SSR (position 60).

**Figure 4:**
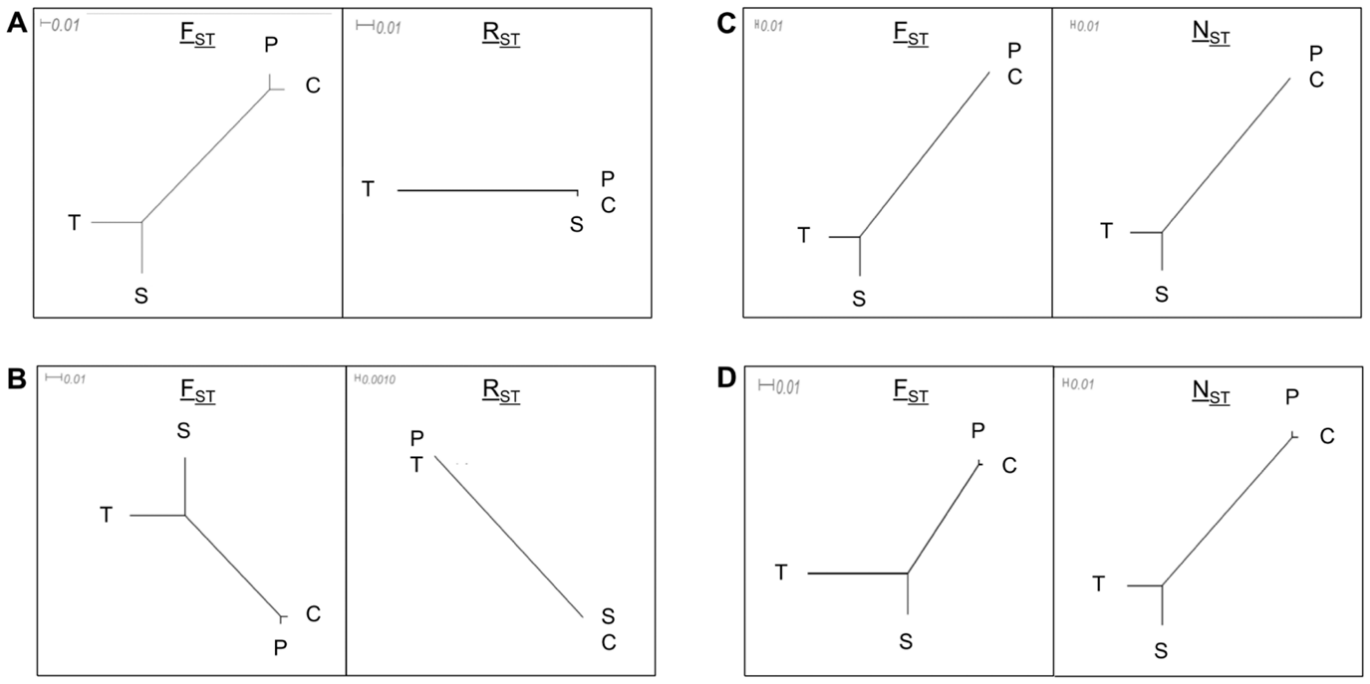
Phylogenetic trees of *Jacaranda* populations based on different components of simple sequence repeat (SSR) data. SSR variation (A), amplicon size variation (B), flanking region (FR) sequence variation (C) and amplicon sequence variation (D). Each type of data was analysed according to a pair of suitable genetic distance estimators: F_ST_, suitable for loci following the infinite allele model (IAM); R_ST_, for loci following the stepwise mutation model (SMM); and N_ST_, for loci following the infinite site model (ISM) model. There are four geographic populations: Counami (*C*), Paracou (*P*) and Saint-Laurent (*S*) in French Guiana; Tapajos (*T*) in Brazil. Note that scales are not the same among trees.

### Correlation between Genetic Distance Matrices

We tested whether different sources of variation conveyed the same information on relatedness among alleles. To do so, genetic distances were computed for each pair of individuals with each source of variation (SSR variation, amplicon size, FR sequence, and amplicon sequence variation) within each marker, and the correlation between genetic distance matrices was tested by a Mantel test. Among the 36 pairs of matrices, 27 (75%) showed a significant correlation (Figure S2). Genetic distance matrices based on SSR variation and on FR sequence variation were the least correlated (one significant pair out of five). Amplicon sequence variation and amplicon size variation, as well as SSR variation and amplicon size variation, were correlated for all markers. The matrices computed on amplicon sequence variation were correlated to the remaining matrices in 95% of the cases (18 out of 19). Conversely, FR sequence variation alone predicted other matrices in only 53% of the cases.

### Population Differentiation

For the *Jacaranda* data set, we tested population genetic differentiation for each source of variation by computing F_ST_, R_ST_ and N_ST_ as appropriate (see Methods; Figure 4). All F_ST_ values were significant. FR sequence variation showed the largest value (0.42) and SSR variation the smallest (0.05). R_ST_ values were very small (<0.02) and non-significant when computed on both amplicon size variation and SSR variation. On the contrary, N_ST_ values were large and significant, with values of 0.50 for FR sequence variation and 0.36 for amplicon sequence variation. In the phylogenetic trees obtained from each combination of source of variation/genetic distance measures, F_ST_ and N_ST_ provided the same topology for all cases they were applied to, indicating a more or less close link between pairs of populations Tapajos/Saint-Laurent and Counami/Paracou (Figure 4). R_ST_ grouped Paracou with Tapajos and Counami with Saint-Laurent (for amplicon size variation) or separated the three Guiana shield populations from Tapajos (for SSR variation; Figure 4).

## Discussion

### Distribution of Polymorphism

SSR polymorphisms are widely used to infer population history and biogeographic patterns. These inferences rely on assumptions about SSR mutational models, and departures from such models are likely to bias divergence and diversity estimates. SSR alleles have been demonstrated to contain other sources of polymorphism in addition to variation in repeat number. In this study we have characterised the contribution of molecular variation occurring outside SSR repeats to SSR marker variability. Whole allele sequencing from SSR markers was analyzed from three tree genera (*Citrus*, *Jacaranda* and *Quercus*). A total of seven (dinucleotide and trinucleotide) SSR markers were targeted, including compound and imperfect markers. More genetic diversity was found for amplicon sequence variation than for amplicon size variation, suggesting that much of data polymorphism is neglected when SSRs are described through amplicon size alone. On average, 7.7 SNPs and 3.4 indels were detected in the flanking sequences of each marker. Genetic diversity (*H_e_*) ranged between 0.306 and 0.922 for the FRs. LD among SNPs and indels in the FR was generally significant. These results demonstrate that SSR alleles are very often riddled with abundant non-SSR indels, plus vast amounts of SNPs both within and near the repeat itself. Similar genetic variation was previously observed at intra- or inter-specific levels [18], [42]–[44] and does not seem to be exceptional in plants. SSR allele mutations are clearly not restricted to the hypervariable tandem repeat region, and polymorphisms outside SSR motifs are also present in animals [22], [45], [46] and humans [21]. Hidden indels can directly confound the estimation of repeat number from amplicon size (“allele size homoplasy”). While undetected SNPs do not directly influencing repeat number estimates, they do cause haplotypes with different evolutionary histories to be treated as if they were identical. Both forms of homoplasy can have profound consequences on the way data should be interpreted.

### Correlation of Polymorphism at the Individual-allele Level

Our results show that pieces of genetic information carried by the different portions of an amplicon are only partially correlated. In particular, blocks of linkage disequilibrium never cover the whole amplicon, and matrices of allele-level pairwise genetic distances built on each source of variation are not always well correlated. In other terms, the different sources of variation are not all equally capable to represent the “true” allele genealogy. Not surprisingly, whole-amplicon sequences were the most parsimonious predictors of relatedness described by the different parts of the amplicons in all our data sets; conversely, FR sequences alone did not adequately synthesize the information from the whole amplicon sequence. Correlation between genetic distances as obtained from FR sequence variation and from SSR variation was globally weak (Figure S2). This recalls the finding of Payseur and Cutter [25] which stated that coalescence times for SSRs and linked SNPs were uncorrelated. Thus, analysing all parts of SSR amplicons increases the amount of available, phylogenetically independent information, with the additional advantage of providing data from DNA regions with different mutation rates. Nevertheless, the correlation analysis shows that the best predictor of amplicon size is SSR length. This indicates that amplicon size is a reliable first-approximation proxy for SSR repeat length, even though extensive homoplasy blurs the correlation.

### Correlation of Polymorphism at the Population Level

The consequences – and usefulness – of hidden sequence variation are shown when we compare how sources of variation perform in detecting population divergence. This analysis was performed in the *Jacaranda* data set comprising four populations. In this example, two alternative patterns were expected for population relatedness: (a) a strictly geographical clustering (the three Guiana shield populations cluster and are separated from the Amazonian population), and (b) a pattern derived from independent results on chloroplast DNA divergence (Counami and Paracou populations form a group, Tapajos and Saint-Laurent form another group (Caroline Scotti-Saintagne, INRA, UMR « Ecologie des forêts de Guyane », article under second round of revision for *Journal of Biogeography*). Our results support the latter hypothesis, as proven by the convergent topology of trees obtained with F_ST_ and N_ST_ independently of the source of variation. The purely geographical hypothesis, on the other hand, is only supported by SSR variation in combination with R_ST_ (although with a non-significant global R_ST_ value). R_ST_ estimates have larger variance than F_ST_ and N_ST_, particularly when based on small numbers of loci, and R_ST_ values obtained here may be unreliable. Nevertheless, the pattern of R_ST_ differentiation closely follows geographical distance. This may indicate that repeat number variation has arisen locally from small numbers of founding alleles and reflects recent population divergence, while variation in flanking regions may follow larger phylogeographic patterns and reflect deeper divergence. If R_ST_ estimated can be trusted, all sources of variation converge with independent, chloroplast-based results, except SSR variation itself. Therefore, information derived from the “true” nature of SSR data (SSR variation) is actually not representative of the information conveyed by alleles as a whole. This finding strongly hints that other sources of variability, more correctly described by mutation models that apply to DNA sequences, actually provide the bulk of information carried by SSRs. In other words, phylogeographic information carried by amplicon length is not provided by the SSR (which amplicon length is intended to represent) but by variation in flanking regions. Note that this contrasts with analyses carried out at the individual level (see above), where information on genetic distance match when measured as amplicon size and as SSR size. This issue raises the question of the application of the SMM, or other step-based mutational models, to SSRs, as already discussed by Colson and Goldstein [47] and Ellegren [12]. At the same time, these results substantiate the hypothesis of Cornuet *et al.* [48], who postulated that not all SSRs would evolve under the same mutational model; additional mutational models are required to appropriately handle variation in SSR data produced from different genera. Moreover, variation in the FRs contributed significantly to the phylogenetic signal and sometimes represented the main source of differentiation among individuals and populations, as shown by *Jacaranda* SSR markers and by the *Citrus* SSR marker GT03 [27].

### Conclusion

Given the complexity of amplicon sequences described here, one may wonder how to properly exploit SSR data. The weight of polymorphisms other than variation in repeat number cannot and should not be overlooked: identity in allele size does not necessarily indicate identity in sequence content or the number of repeats within same sized alleles. When considering individual alleles, amplicon size correlates well with SSR repeat number. Nevertheless, even if repeat number could be obtained directly, it may provide only weak and inconsistent signals of population genetic differentiation, as shown by the *Jacaranda* data set (but larger numbers of markers may reduce estimation variance and mitigate this problem). Taking sequence variation into account actually adds a significant piece of information to phylogenetic or phylogeographic reconstruction. Population structuring emerges more clearly on the basis of sequence data, and the combination of sequences and SSR variation provides higher resolution. Consequently, genetic distance measures assuming the SMM or related models should be restricted to perfect SSRs with invariable FRs. On the other hand, measures such as F_ST_ (for amplicon size data) and N_ST_ (for amplicon sequences) seem to be the tool of choice for the analysis of the average, not-so-ideal SSR markers that a population geneticist meets in his or her everyday work.

## Supporting Information

Figure S1 **Complete alignments of DNA fragments for all SSR markers studied**. Marker names are shown above each alignment.
(PDF)

Figure S2 **Z-statistics and** **summary of Mantel tests for the correlations of pairs of genetic distance matrices within species.** Colouring (shading) indicates the level of significance of the test: green (light grey*), *P*>0.05; orange (grey*), 0.05<*P*>0.001; red (dark grey*), *P*<0.001. Identity of DNA fragment sections is reported: simple sequence repeat (SSR) variation; amplicon size variation, flanking region (FR) sequence variation, and amplicon sequence variation. Marker names are displayed above each panel and data sets refer to *Citrus* (C), *Jacaranda* (J) and *Quercus* (Q). Sequences with single nucleotide polymorphisms (SNPs) within the repeat were excluded from subsequent analyses because of the complexity to describe the mutation model of SSR repeats bracketing SNPs.
(PDF)

Table S1 **List and details of the sampled sites for the** ***Jacaranda*** **(J) data set.**
(DOC)

Table S2 **Description, amplification conditions and polymorphism of the nuclear simple sequence repeats (SSRs) analysed in** ***Jacaranda copaia*** **.**
(DOC)

Table S3 **List and details of alleles for the three data sets.** Amplicon sizes, frequencies and number of associated haplotypes are indicated for each allele.
(DOC)
