# Supplementary material for: Always Look on Both Sides: Phylogenetic Information Conveyed by Simple Sequence Repeat Allele Sequences
Source: PLoS One. 2012 Jul 13;7(7):e40699. doi: 10.1371/journal.pone.0040699 (PMC3396589; doi:10.1371/journal.pone.0040699)
Supplement: Figure S1 — Complete alignments of DNA fragments for all SSR markers studied. Marker names are shown above each alignment. (PDF) [file pone.0040699.s001.pdf]

CCT01

```
1225F_Cmax_pum_Hun     TCAACACCTCGAACAGAAGGTTCTCGTCCAGATTTGTGAAGCCAGTTTCTTCGGATTGCTCTCCCTCTTCCTCCTCCACATCCACCTCCTCCTCCTCTTGTTTGACTAACTTCCAAGTGAAAATTTTCAAAAACTCAT
1225R_Cmax_pum_Hun     TCAACACCTCGAACAGAAGGTTCTCGTCCAGATTTGTGAAGCCAGTTTCTTCGGATTGCTCTCCCTCTTCCTCCTCCACATCCACCTCCTCCTCCTCTTGTTTGACTAACTTCCAAGTGAAAATTTTCAAAAACTCAT
2341F_Cmax_pum_KLY     TCAACACCTCGAACAGAAGGTTCTCGTCCAGATTTGTGAAGCCAGTTTCTTCGGATTGCTCTCCCTCTTCCTCCTCCACCTCCACCTCCTCCTCCTCTTGTTTGACTAACTTCCAAGTGAAAATTTTCAAAAACTCAT
2341R_Cmax_pum_KLY     TCAACACCTCGAACAGAAGGTTCTCGTCCAGATTTGTGAAGCCAGTTTCTTCGGATTGCTCTCCCTCTTCCTCCTCCACCTCC---TCCTCCTCCTCTTGTTTGACTAACTTCCAAGTGAAAATTTTCAAAAACCCAT
2355F_Cmax_pum_KaP     TCAACACCTCGAACAGAAGGTTCTCGTCCAGATTTGTGAAGCCAGTTTCTTCGGATTGCTCTCCCTCTTCCTCCTCCACCTCCACCTCCTCCTCCTCTTGTTTGACTAACTTCCAAGTGAAAATTTTCAAAAACCCAT
2355R_Cmax_pum_KaP     TCAACACCTCGAACAGAAGGTTCTCGTCCAGATTTGTGAAGCCAGTTTCTTCGGATTGCTCTCCCTCTTCCTCCTCCACCTCC---TCCTCC---TCTTGTTTGACTAACTTCCAAGTGAAAATTTTCAAAAACTCAC
2554F_Ptri_tri_Bar     TCAACACCTCGAACAGAAGGTCCTCGTCCAGACTTGTGAAGCCAGTTTCTTCGGATTGCTCTCTCTCTTCCTCCTCCACCTCC---TCCTCC---TCTTGTTTGACTAACTTCCAAGTGAGAATTTTCAAAAACTCAT
2554R_Ptri_tri_Bar     TCAACACCTCGAACAGAAGGTCCTCGTCCAGACTTGTGAAGCCAGTTTCTTCGGATTGCTCTCTCTCTTCCTCCTCCACCTCC---TCCTCC---TCTTGTTTGACTAACTTCCAAGTGAGAATTTTCAAAAACTCAT
300F_Cret_man_Par      TCAACACCTCGAACAGAAGGTTCTCGTCCAGATTTGTGAAGCCAGTTTCTTCGGATTGCTCTCCCTCTTCCTCCTCCACCTCC---TCCTCC---TCTTGTTTGACTAACTTCCAAGTGAAAATTTTCAAAAACTCAC
300R_Cret_man_Par      TCAACACCTCGAACAGAAGGTTCTCGTCCAGATTTGTGAAGCCAGTTTCTTCGGATTGCTCTCCCTCTTCCTCCTCCACCTCC---TCCTCC---TCTTGTTTGACTAACTTCCAAGTGAAAATTTTCAAAAACTCAC
3055F_Cmed_citr_Be     TCAACACCTCGAACAGAAGGTTCTCGTCCAGATTTGTGAAGCCAGTTTCTTCGGATTGCTCTCCCTCTTCCTCCTCCACCTCC---TCCTCCTCCTCTTGTTTGACTAACTTCCAAGTGGAAATTTTCAAAAACTCAT
3055R_Cmed_citr_Be     TCAACACCTCGAACAGAAGGTTCTCGTCCAGATTTGTGAAGCCAGTTTCTTCGGATTGCTCTCCCTCTTCCTCCTCCACCTCC---TCCTCC---TCTTGTTTGACTAACTTCCAAGTAAAAATTTTCAAAAACTCAT
3056F_Csp_pap_Unn      TCAACACCTCGAACAGAAGGTTCTCGTCCAGATTTGTGAAGCCAGTTTCTTCGGATTGCTCTCCCTCTTCCTCCTCCACCTCC---TCC------TCTTGTTTGACTAACTTCCAAGTGAAAATTTTCAAAAACTCAT
3056R_Csp_pap_Unn      TCAACACCTCGAACAGAAGGTTCTCGTCCAGATTTGTGAAGCCAGTTTCTTCGGATTGCTCTCCCTCTTCCTCCTCCACCTCC---TCC------TCTTGTTTGACTAACTTCCAAGTGAAAATTTTCAAAAACTCAT
3066F_Cmax_pum_Sou     TCAACACCTCGAACAGAAGGTTCTCGTCCAGATTTGTGAAGCCAGTTTCTTCGGATTGCTCTCCCTCTTCCTCCTCCACCTCCACCTCCTCCTCCTCTTGTTTGACTAACTTCCAAGTGAAAATTTTCAAAAACTCAT
3066R_Cmax_pum_Sou     TCAACACATCGAACAGAAGGTTCTCGTCCAGATTTGTGAAGCCAGTTTCTTCGGATTGCTCTCCCTCTTCCTCCTCCACCTCC---TCCTCC---TCTTGTTTGACTAACTTCCAAGTAAAAATTTTCAAAAACTCAT
3237F_Fjap_kum_Unn     TCAACACCTCGAACAGAAGGCTCTCGTCCAGATTTGTGAAGCCAGTTTCTTCGGATTGCTCTCCCTCTTCCTCCTCCACCTCC---TCCTCCTCCTCTTGTTTGACTAACTTCCAAGTGAAAAATTTCAAAAACTCAT
3237R_Fjap_kum_Unn     TCAACACCTCGAACAGAAGGTTCTCGTCCAGATTTGTGAAGCCAGTTTCTTCGGATTGCTCTCCCTCTTCCTCCTCCATCTCC---TCCTTC---TCTTGTTTGACTAACTTCCAAGTGAAAAATTTCAAAAACTCAT
3780F_Chal_cithyb_U    TCAACACCTCGAACAGAAGGTTCTCGTCCAGATTTGTGAAGCCAGTTTCTTCGGATTGCCCTCCCTCTTCCTCCTCCATCTCC---TCC------TCTTGTTTGACTAACTTCCAAGTGAAAAATTTCAAAAACTCAT
3780R_Chal_cithyb_U    TCAACACCTCGAACAGAAGGTTCTCGTCCAGATTTGTGAAGCCAGTTTCTTCGGATTGCCCTCCCTCTTCCTCCTCCATCTCC---TCC------TCTTGTTTGACTAACTTCCAAGTGAAAAATTTCAAAAACTCAT
3793F_Csp_pap_Unn      TCAACACCTCGAACAGAAGGTTCTCGTCCAGATTTGTGAAGCCAGTTTCTTCGGATTGCTCTCCCTCTTCCTCCTCCACCTCC---TCCTCCTCCTCTTGTTTGACTAACTTCCAAGTGAAAATTTTCAAAAACCCAT
3793R_Csp_pap_Unn      TCAACACCTCGAACAGAAGGTTCTCGTCCAGATTTGTGAAGCCAGTTTCTTCGGATTGCTCTCCCTCTTCCTCCTCCATCTCC---TCC------TCTTGTTTGACTAACTTCCAAGTGAAAAATTTCAAAAACCCAT
3797F_Csp_pap_Unn      TCAACACCTCGAACAGAAGGTTCTCGTCCAGATTTGTGAAGCCAGTTTCTTCGGATTGCTCTCCCTCTTCCTCCTCCATCTCC---TCC------TCTTGTTTGACTAACTTCCAAGTGAAAAATTTCAAAAACCCAT
3797R_Csp_pap_Unn      TCAACACCTCGAACAGAAGGTTCTCGTCCAGATTTGTGAAGCCAGTTTCTTCGGATTGCTCTCCCTCTTCCTCCTCCATCTCC---TCC------TCTTGTTTGACTAACTTCCAAGTGAAAAATTTCAAAAACCCAT
3816F_Cret_man_KiU     TCAACACCTCGAACAGAAGGTTCTCGTCCAGATTTGTGAAGCCAGTTTCTTCGGATTGCTCTCCCTCTTCCTCCTCCACCTCC---TCCTCCTCCTCTTGTTTGACTAACTTCCAAGTGAAAATTTTCAAAAACTCAT
3816R_Cret_man_KiU     TCAACACCTCGAACAGAAGGTTCTCGTCCAGGTTTGTGAAGCCAGTTTCTTCGGATTGCTCTCCCTCTTCCTCCTCCACCTCC---TCCTCC---TCTTGTTTGACTAACTTCCAAGTGAAAATTTTCAAAAACTCAT
448F_Cmax_pum_Moa      TCAACACCTCGAACAGAAGGTTCCCGTCCAGATTTGTGAAGCCAGTTTCTTCGGATTGCTCTCCCTCTTCCTCCTCCACCTCC---TCCTCCTCCTCTTGTTTGACTAACTTCCAAGTGAAAATTTTCAAAAACTCAT
448R_Cmax_pum_Moa      TCAACACCTCGAACAGAAGGTTCCCGTCCAGATTTGTGAAGCCAGTTTCTTCGGATTGCTCTCCCTCTTCCTCCTCCACCTCC---TCCTCCTCCTCTTGTTTGACTAACTTCCAAGTGAAAATTTTCAAAAACTCAT
578F_Cmax_pum_FlS      TCAACACCTCGAACAGAAGGTTCTCGTCCAGATTTGTGAAGCCAGTTTCTTCGGATTGCTCTCCCTCTTCCTCCTCCACCTCCACCTCCTCCTCCTCTTGTTTGACTAACTTCCAAGTGAAAATTTTCAAAAACTCAT
578R_Cmax_pum_FlS      TCAACACCTCGAACAGAAGGTTCTCGTCCAGATTTGTGAAGCCAGTTTCTTCGGATTGCTCTCCCTCTTCCTCCTCCACCTCCACCTCCTCCTCCTCTTGTTTGACTAACTTCCAAGTGAAAATTTTCAAAAACTCAT
644F_Cmax_pum_Phi      TCAACACCTCGAACAGAAGGTTCTCGTCCAGATTTGTGAAGCCAGTCTCTTTGGATTGCTCTCCCTCTTCCTCCTCCACCTCC---TCCTCCTCTTCTTGTTTGACTAACTTCCAAGTGAAAATTTTCAAAAACTCAT
644R_Cmax_pum_Phi      TCAACACCTCGAACAGAAGGTTCTCGTCCAGATTTGTGAAGCCAGTTTCTTCGGATTGCTCTCCCTCTTCCTCCTCCACC---ACCTCCTCC---TCTTGTTTGACTAACTTCCAAGTGAAAATTTTCAAAAACTCAT
661F_Cmed_cit_Ind      TCAACACCTCGAACAGAAGGTTCTCGTCCAGATTTGTGAAGCCAGTTTCTTCGGATTGCTCTCCCTCTTCCTCCTCCACCTCC---TCCTCC---TCTTGTTTGACTAACTTCCAAGTGAAAATTTTCAAAAACTCAT
661R_Cmed_cit_Ind      TCAACACCTCGAACAGAAGGTTCTCGTCCAGATTTGTGAAGCCAGTTTCTTCGGATTGCTCTCCCTCTTCCTCCTCCACCTCC---TCCTCC---TCTTGTTTGACTAACTTCCAAGTGAAAATTTTCAAAAACTCAT

TGCTGCAATTCTTTGTGCTAGCATGTGGG
TGCTGCAATTCTTTGTGCTAGCATGTGGG
TGCTGCAATTCTTTGTGCTAGCATGTGGG
TGCTGCAATTCTTTGTGCTAGCATGTGGG
TGCTGCAATTCTTTGTGCTAGCATGTGGG
TGCTGCAATTCTTTGTGCTAGCATGTGGG
TGCTGCAATTCTTTGTGCTAGCATGTGGG
TGCTGCAATTCTTTGTGCTAGCATGTGGG
TGCTGCAATTCTTTGTGCTAGCATGTGGG
TGCTGCAATTCTTTGTGCTAGCATGTGGG
TGCTGCAATTCTTTGTGCTAGCATGTGGG
TGCTGCAATTCTTTGTGCTAGCATGTGGG
TGCTGCAATTCTTTGTGCTAGCATGTGGG
TGCTGCAATTCTTTGTGCTAGCATGTGGG
TGCTGCAATTCTTTGTGCTAGCATGTGGG
TGCCGCAATTCTTTGTGCTAGCATGTGGG
TGCTGCAATTCTTTGTGCTAGCATGTGGG
TGCTGCAATTCTTTGTGCTAGCATGTGGG
TGCTGCAATTCTTTGTGCTAGCATGTGGG
TGCTGCAATTCTTTGTGCTAGCATGTGGG
```

```
TGCTGCAATTCTTTGTGCTAGCATGTGGG
TGCTGCAATTCTTTGTGCTAGCATGTGGG
TGCTGCAATTCTTTGTGCTAGCATGTGGG
TGCTGCAATTCTTTGTGCTAGCATGTGGG
TGCTGCAATTCTTTGTGCTAGCATGTGGG
TGCTGCAATTCTTTGTGCTAGCATGTGGG
TGCTGCAATTCTTTGTGCTAGCATGTGGG
TGCTGCAATTCTTTGTGCTAGCATGTGGG
TGCTGCAATTCTTTGTGCTAGCATGTGGG
TGCTGCAATTCTTTGTGCTAGCATGTGGG
TGCTGCAATTCTTTGTGCTAGCATGTGGG
TGCTGCAATTCTTTGTGCTAGCATGTGGG
TGCTGCAATTCTTTGTGCTAGCATGTGGG
TGCTGCAATTCTTTGTGCTAGCATGTGGG
```

cAGG9

```
2554F_Ptri_tri_Bar     AATGCTGAAGATAATCCGCGAAATGAGTATCCAGATGAGATTTCTGATGAGCAAGAGGAGGAGGAGGAGGAGGAAGAAGAAGAAGAA---GAGGTAGATGAAGATGGAGTGGAGAGCAAGGCA
2554R_Ptri_tri_Bar     AATGCTGAAGATAATCCGCGAAATGAGTATCCAGATGAGATTTCTGATGAGCAAGAGGAGGAGGAGGAGGAGGAAGAAGAAGAAGAA---GAGGTAGATGAAGATGGAGTGGAGAGCAAGGCA
4008F_Ptri_tri_Seed    AATGCTGAAGATAATCCGCGAAATGAGTATCCAGATGAGATTTCTGATGAGCAAGAGGAGGAGGAGGAGGAGGAAGAAGAAGAAGAA---GAGGTAGATGAAGATGGAGTGGAGAGCAAGGCA
4008R_Ptri_tri_Seed    AATGCTGAAGATAATCCGCGAAATGAGTATCCAGATGAGATTTCTGATGAGCAAGAGGAGGAGGAGGAGGAGGAAGAAGAAGAAGAA---GAGGTAGATGAAGATGGAGTGGAGAGCAAGGCA
2240F_Cmax_pum_SiA     AATGCTGAAGATAATCCGCGAAATGAGTATCCAGATGAGATTTCTGATGAGCAAGAGGAGGAGGAGGAGGAGGAAGAAGAAGAAGAA---GAGGTAGATGAAGATGGAGTGGAGAGCAAGGCA
2240R_Cmax_pum_SiA     AATGCTGAAGATAATCCGCGAAATGAGTATCCAGATGAGATTTCTGATGAGCAAGAGGAGGAGGAGGAGGAGGAAGAAGAAGAAGAA---GAGGTAGATGAAGATGGAGTGGAGAGCAAGGCA
3816F_Cret_man_KiU     AATGCTGAAGATAATCCGCGAAATGAGTATCCAGATGAGATTTCTGATGAGCAAGAGGAGGAGGAGGAGGAGGAAGAAGAAGAAGAA---GAGGTAGATGAAGATGGAGTGGAGAGCAAGGCA
3816R_Cret_man_KiU     AATGCTGAAGATAATCCGCGAAATGAGTATCCAGATGAGATTTCTGATGAGCAAGAGGAGGAGGAGGAG---GAAGAAGAAGAAGAA---GAGGTAGATGAAGATGGAGTGGAGAGCAAGGCA
1208F_Cmax_pum_RoP     AATGCTGAAGATAATCCGCGAAATGAGTATCCAGATGAGATTTCTGATGAGCAAGAGGAGGAGGAGGAGGAGGAAGAAGAAGAAGAA---GAGGTAGATGAAGATGGAGTGGAGAGCAAGGCA
1208R_Cmax_pum_RoP     AATGCTGAAGATAATCCGCGAAATGAGTATCCAGATGAGATTTCTGATGAGCAAGAGGAGGAGGAGGAG---GAAGAAGAAGAAGAA---GAGGTAGGTGAAGATGGAGTGGAGAGCAAGGCA
3845F_Cret_man_Kin     AATGCTGAAGATAATCCGCGAAATGAGTATCCAGATGAGATTCCTGATGAGCAAGAGGAGGAGGAG------GAAGAAGAAGAAGAAGAAGAGGTAGATGAAGATGGAGTGGAGAGCAAGGCA
3845R_Cret_man_Kin     AATGCTGAAGATAATCCGCGAAATGAGTATCCAGATGAGATTTCTGATGAGCAAGAGGAGGAGGAGGAG---GAAGAAGAAGAA------GAGGTAGATGAAGATGGAGTGGAGAGCAAGGCA
3055F_Cmed_cit_Ben     AATGCTGAAGATAATCCGCGAAATGAGTATCCAGATGAGATTTCTGATGAGCAAGAGGAGGAGGAGGAG---GAAGAAGAAGAAGAA---GAGGTAGATGAAGATGGAGTGGAGAGCAAGGCA
3055R_Cmed_cit_Ben     AATGCTGAAGATAATCCGCGAAATGAGTATCCAGATGAGATTTCTGATGAGCAAGAGGAGGAGGAGGAG---GAAGAAGAAGAAGAA---GAGGTAGATGAAGATGGAGTGGAGAGCAAGGCA
279F_Cret_man_Clem     AATGCTGAAGATAATCCGCGAAATGAGTATCCAGATGAGATTTCTGATGAGCAAGAGGAGGAGGAGGAG---GAAGAAGAAGAAGAA---GAGGTAGATGAAGATGGAGTGGAGAGCAAGGCA
279R_Cret_man_Clem     AATGCTGAAGATAATCCGCGAAATGAGTATCCAGATGAGATTTCTGATGAGCAAGAGGAGGAGGAGGAG---GAAGAAGAAGAAGAA---GAGGTAGATGAAGATGGAGTGGAGAGCAAGGCA
3326F_Cret_man_ScE     AATGCTGAAGATAATCCGCGAAATGAGTATCCAGATGAGATTTCTGATGAGCAAGAGGAGGAGGAGGAG---GAAGAAGAAGAAGAA---GAGGTAGATGAAGATGGAGTGGAGAGCAAGGCA
3326R_Cret_man_ScE     AATGCTGAAGATAATCCGCGAAATGAGTATCCAGATGAGATTTCTGATGAGCAAGAGGAGGAGGAGGAG---GAAGAAGAAGAAGAA---GAGGTAGATGAAGATGGAGTGGAGAGCAAGGCA
3147F_Cret_man_Unn     AATGCTGAAGATAATCCGCGAAATGAGTATCCAGATGAGATTTCTGATGAGCAAGAGGAGGAGGAG------GAAGAAGAAGAAGAA---GAGGTAGATGAAGATGGAGTGGAGAGCAAGGCA
3147R_Cret_man_Unn     AATGCTGAAGATAATCCGCGAAATGAGTATCCAGATGAGATTTCTGATGAGCAAGAGGAGGAGGAG------GAAGAAGAAGAAGAA---GAGGTAGATGAAGATGGAGTGGAGAGCAAGGCA
3469F_Chan_pap_Han     AATGCTGAAGATAATCCGCGAAATGAGTATCCAGATGAGATTTCTGATGAGCAAGAGGAGGAGGAG------GAAGAAGAAGAAGAA---GAGGTAGATGAAGATGGAGTGGAGAGCAAGGCA
3469R_Chan_pap_Han     AATGCTGAAGATAATCCGCGAAATGAGTATCCAGATGAGATTTCTGATGAGCAAGAGGAGGAGGAG------GAAGAAGAAGAAGAA---GAGGTAGATGAAGATGGAGTGGAGAGCAAGGCA
3150F_Cret_man_Unn     AATGCTGAAGATAATCCGCGAAATGAGTATCCAGATGAGATTTCTGATGAGCAAGAGGAGGAGGAG------GAAGAAGAAGAAGAA---GAGGTAGATGAAGATGGAGTGGAGAGCAAGGCA
3150R_Cret_man_Unn     AATGCTGAAGATAATCCGCGAAATGAGTATCCAGATGAGATTTCTGATGAGCAAGAGGAGGAGGAG------GAAGAAGAAGAAGAA---GAGGTAGATGAAGATGGAGTGGAGAGCAAGGCA
131F_Clim_ran_SaB      AATGCTGAAGATAATCCGCGAAATGAGTATCCAGATGAGATTTCTGATGAGCAAGAGGAGGAGGAG------GAAGAAGAAGAAGAA---GAGGTAGATGAAGATGGAGTGGAGAGCAAGGCA
131R_Clim_ran_SaB      AATGCTGAACATAATCCGCGAAATGAGTATCCAGATGAGATTTCGGATGAGCAAGAGGAGGAG---------GAAGAAGAA---------GAGGTAGATGAAGATGGAGTGGAGAGCAAGGCA
3163F_Cret_man_IWO     AATGCTGAAGATAATCCGCGAAATGAGTATCTAGATGAGATTTCGGATGAGCAAGAGGAGGAG---------GAAGAAGAA---------GAGGTAGATGAAGATGGAGTGGAGAGCAAGGCA
3163R_Cret_man_IWO     AATGCTGAAGATAATCCGCGAAATGAGTATCTAGATGAGATTTCGGATGAGCAAGAGGAGGAG---------GAAGAAGAA---------GAGGTAGATGAAGATGGAGTGGAGAGCAAGGCA
138F_Cmed_cit_Ind      AATGCTGAAGATAATCCGCGAAATGAGTATCCAGATGAGATTTCGGATGAGCAAGAGGAGGAG---------GAAGAAGAA---------GAGGTAGATGAA-ATGGAGTGGAGAGCAAGGCA
138R_Cmed_cit_Ind      AATGCTGAAGATAATCCGCGAAATGAGTATCCAGATGAGATTTCGGATGAGCAAGAGGAGGAG---------GAAGAAGAA---------GAGGTAGATGAA-ATGGAGTGGAGAGCAAGGCA
2875F_Cmed_cit_Jap     AATGCTGAAGATAATCCGCGAAATGAGTATCCAGATGAGATTTCGGATGAGCAAGAGGAGGAG---------GAAGAAGAA---------GAGGTAGATGAAGATGGAGTGGAGAGCAAGGCA
2875R_Cmed_cit_Jap     AATGCTGAAGATAATCCGCGAAATGAGTATCCAGATGAGATTTCGGATGAGCAAGAGGAGGAG---------GAAGAAGAA---------GAGGTAGATGAAGATGGAGTGGAGAGCAAGGCA
3527F_Cmed_cit_Hia     AATGCTGAAGATAATCCGCGAAATGAGTATCCAGATGAGATTTCAGATGAGCAAGAGGAGGAG---------GAAGAAGAA---------GAGGTAGATGAAGATGGAGTGGAGAGCAAGGCA
3527R_Cmed_cit_Hia     AATGCTGAAGATAATCCGCGAAATGAGTATCCAGATGAGATTTCAGATGAGCAAGAGGAGGAG---------GAAGAAGAA---------GAGGTAGATGAAGATGGAGTGGAGAGCAAGGCA
```

GT03

```
1224F_Cmax_pum_Unn    GCCTTCTTGATTTAACCGGACAAAGAATGGTAAATATATCTTACTAGTAGTGTGTTTCTGTGTGTGTGTGTGTGTGTGTGT----------AATCTTTGGAGAACTCTCTCATTGATTAAGACCCATTTTATCTAC
1224R_Cmax_pum_Unn    GCCTTCTTGATTTAACCGGACAAAGAATGGTAAATATATCTTACTAGTAGTGTGTTTCTGTGTGTGTGTGTGTGTGTGTGT----------AATCTTTGGAGAACTCTCTCATTGATTAAGACCCATTTTATCTAC
1471F_Fcra_kum_Mei    GCCTTCTTGATTTAACCGGACAAAGAATGGTAAATATATCTTACTAGTAGTGTGTTTCTGTGTGTATGTGTGTGTGTGTGTGT----------AATCTTTGGAGAACTCTCTCATTGATTAGGACCCATTTTATCTAC
1471R_Fcra_kum_Mei    GCCTTCTTGATTTAACCGGACAAAGAATGGTAAATATATCTTACTAGTAGTGTGTTTCTGTGTGTATGTGTGTGTGTGTGTGT----------AATCTTTGGAGAACTCTCTCATTGATTAGGACCCATTTTATCTAC
2554F_Ptri_tri_Bar    GCCTTCTTGATTTAACCGGACAAAGAATGGTAAATATATCTTACTAGTAGTGTGTTTCTGTGTGTGTGTGTGTGTGTGTGTGTGTGT----AATCTTTGGAGAACTCTCTCATTGATTAAGACCCATTTTATTTAC
2554R_Ptri_tri_Bar    GCCTTCTTGATTTAACCGGACAAAGAATGGTAAATATATCTTACTAGTAGTGTGTTTCTGTGTGTGTGTGTGTGTGTGTGTGTGTGT----AATCTTTGGAGAACTCTCTCATTGATTAAGACCCATTTTATTTAC
2867F_Cret_man_Cal    GCCTTCTTGATTTAACCGGACAAAGAATGGTAAATATAAGTTACTAGTCGTGTGTATCTGTGTGT------------------------ATATAATCTTTGGAGAACTCTCTCCTTGATTAAGACCCATTTTATCTAC
2867R_Cret_man_Cal    GCCTTCTTGATTTAACCGGACAAAGAATGGTAAATATAAGTTACTAGTCGTGTGTATCTGTGTGT------------------------ATATAATCTTTGGAGAACTCTCTCCTTGATTAAGACCCATTTTATCTAC
2875F_Cmed_cit_Jap    GCCTTCTTGATTTAACCGGACAAAGAATGGTAAATATATCTTACTAGTCGTGTGT----GTGTGTGTGT------------------------GATCTTTGGAGAACTCTCTCATTGATTAAGACCCATTTTATCTAC
2875R_Cmed_cit_Jap    GCCTTCTTGATTTAACCGGACAAAGAATGGTAAATATATCTTACTAGTCGTGTGT----GTGTGTGTGT------------------------GATCTTTGGAGAACTCTCTCATTGATTAAGACCCATTTTATCTAC
3151F_Ptri_tri_Aus    GCCTTCTTGATTTAACCGGACAAAGAATGGTAAATATATCTTACTAGTAGTGTGTTTCTGTGTGTGTGTGTGTGTGTGTGTGTGT------AATCTTTGGAGAACTCTCTCATTGATTAAGACCCATTTTATTTAC
3151R_Ptri_tri_Aus    GCCTTCTTGATTTAACCGGACAAAGAATGGTAAATATATCTTACTAGTAGTGTGTTTCTGTGTGTGTGTGTGTGTGTGTGTGTGT------AATCTTTGGAGAACTCTCTCATTGATTAAGACCCATTTTATTTAC
3351F_Ptri_tri_Fai    GCCTTCTTGATTTAACCGGACAAAGAATGGTAAATATATCTTACTAGTAGTGTGTTTCTGTGTGTGTGTGTGTGTGTGTGTGTGTGT----AATCTTTGGAGAACTCTCTCATTGATTAAGACCCATTTTATTTAC
3351R_Ptri_tri_Fai    GCCTTCTTGATTTAACCGGACAAAGAATGGTAAATATATCTTACTAGTAGTGTGTTTCTGTGTGTGTGTGTGTGTGTGTGTGTGTGT----AATCTTTGGAGAACTCTCTCATTGATTAAGACCCATTTTATTTAC
3549F_Ptri_tri_Sim    GCCTTCTTGATTTAACCGGACAAAGAATGGTAAATATATCTTACTAGTAGTGTGTTTCTGTGTGTGTGTGTGTGTGTGTGTGTGTGT----AATCTTTGGAGAACTCTCTCATTGATTAAGACCCATTTTATTTAC
3549R_Ptri_tri_Sim    GCCTTCTTGATTTAACCGGACAAAGAATGGTAAATATATCTTACTAGTAGTGTGTTTCTGTGTGTGTGTGTGTGTGTGTGTGTGTGT----AATCTTTGGAGAACTCTCTCATTGATTAAGACCCATTTTATTTAC
3789F_Fhin_kum_Unn    GCCTTCTTGATTTAACCGGACAAAGAATGGTAAATATATCTTACTAGTAGTGTGTTTCTGTGTGTATGTGTGTGTGTGTGTGT----------AATCTTTGGAGAACTCTCTCATTGATTAGGACCCATTTTATCTAC
3789R_Fhin_kum_Unn    GCCTTCTTGATTTAACCGGACAAAGAATGGTAAATATATGTTACTAGTCGTGTGTTTCTGTGTGTGT-----------------------ATAATCTTTGGAGAACTCTCTCCTTGATTAAGACCCATTTTATCTAC
3790F_Fhin_kum_BB     GCCTTCTTGATTTAACCGGACAAAGAATGGTAAATATATGTTACTAGTCGTGTGTTTCTGTGTGT------------------------ATATAATCTTTGGAGAACTCTCTCCTTGATTAAGACCCATTTTATCTAC
3790R_Fhin_kum_BB     GCCTTCTTGATTTAACCGGACAAAGAATGGTAAATATATGTTACTAGTCGTGTGTTTCTGTGTGT------------------------ATATAATCTTTGGAGAACTCTCTCCTTGATTAAGACCCATTTTATCTAC
3818F_Fcra_kum_Mei    GCCTTCTTGATTTAACCGGACAAAGAATGGTAAATATATCTTACTAGTAGTGTGTTTCTGTGTGTATGTGTGTGTGTGTGTGT----------AATCTTTGGAGAACTCTCTCATTGATTAGGACCCATTTTATCTAC
3818R_Fcra_kum_Mei    GCCTTCTTGATTTAACCGGACAAAGAATGGTAAATATATCTTACTAGTAGTGTGTTTCTGTGTGTATGTGTGTGTGTGTGTGT----------AATCTTTGGAGAACTCTCTCATTGATTAGGACCCATTTTATCTAC
3833F_Fcra_kum_Mei    GCCTTCTTGATTTAACCGGACAAAGAATGGTAAATATATCTTACTAGTAGTGTGTTTCTGTGTGTATGTGTGTGTGTGTGTGT----------AATCTTTGGAGAACTCTCTCATTGATTAGGACCCATTTTATCTAC
3833R_Fcra_kum_Mei    GCCTTCTTGATTTAACCGGACAAAGAATGGTAAATATATCTTACTAGTAGTGTGTTTCTGTGTGTATGTGTGTGTGTGTGTGT----------AATCTTTGGAGAACTCTCTCATTGATTAGGACCCATTTTATCTAC
3876F_Ptri_tri_EnD    GCCTTCTTGATTTAACCGGACAAAGAATGGTAAATATATCTTACTAGTAGTGTGTTTCTGTGTGTGTGTGTGTGTGTGTGTGTGTGT----AATCTTTGGAGAACTCTCTCATTGATTAAGACCCATTTTATTTAC
3876R_Ptri_tri_EnD    GCCTTCTTGATTTAACCGGACAAAGAATGGTAAATATATCTTACTAGTAGTGTGTTTCTGTGTGTGTGTGTGTGTGTGTGTGTGTGT----AATCTTTGGAGAACTCTCTCATTGATTAAGACCCATTTTATTTAC
3878F_Cmed_cit_cul    GCCTTCTTGATTTAACCGGACAAAGAATGGTAAATATATCTTACTAGTAGTGTGTTTCTCTGTGTGTGTGTGTGTGTGTGTGTGT------AATCTTTGGAGAACTCTCTCATTGATTAAGACCCATTTTATCTAC
3878R_Cmed_cit_cul    GCCTTCTTGATTTAACCGGACAAAGAATGGTAAATATATCTTACTAGTAGTGTGTTTCTCTGTGTGTGTGTGTGTGTGTGTGTGT------AATCTTTGGAGAACTCTCTCATTGATTAAGACCCATTTTATCTAC
3888F_Ptri_tri_Unn    GCCTTCTTGATTTAACCGGACAAAGAATGGTAAATATATCTTACTAGTAGTGTGTTTCTGTGTGTGTGTGTGTGTGTGTGTGTGT------AATCTTTGGAGAACTCTCTCATTGATTAAGACCCATTTTATTTAC
3888R_Ptri_tri_Unn    GCCTTCTTGATTTAACCGGACAAAGAATGGTAAATATATCTTACTAGTAGTGTGTTTCTGTGTGTGTGTGTGTGTGTGTGTGTGT------AATCTTTGGAGAACTCTCTCATTGATTAAGACCCATTTTATTTAC
3947F_Cmax_pum_SuB    GCCTTCTTGATTTAACCGGACAAAGAATGGTAAATATATCTTACTAGTAGTGTGTTTCTCTGTGTGTGTGTGTGTGTGTGTGTGT------AATCTTTGGAGAACTCTCTCATTGATTAAGACCCATTTTATCTAC
3947R_Cmax_pum_SuB    GCCTTCTTGATTTAACCGGACAAAGAATGGTAAATATATCTTACTAGTAGTGTGTTTCTCTGTGTGTGTGTGTGTGTGTGTGTGT------AATCTTTGGAGAACTCTCTCATTGATTAAGACCCATTTTATCTAC
4006R_Ptri_tri_Seed   GCCTTCTTGATTTAACCGGACAAAGAATGGTAAATATATCTTACTAGTAGTGTGTTTCTGTGTGTGTGTGTGCGTGTGTGTGTGTGT----AATCTTTGGAGAACTCTCTCATTGATTAAGACCCATTTTATTTAC
4006F_Ptri_tri_Seed   GCCTTCTTGATTTAACCGGACAAAGAATGGTAAATATATCTTACTAGTAGTGTGTTTCTGTGTGTGTGTGTGCGTGTGTGTGTGTGT----AATCTTTGGAGAACTCTCTCATTGATTAAGACCCATTTTATTTAC
644F_Cmax_pum_Phi     GCCTTCTTGATTTAACCGGACAAAGAATGGTGAATATATCTTACTAGTAGTGTGTTTCTCTGTGTGTGTGTGTGTGTGTGTGTGT------AATCTTTGGAGAACTCTCTCATTGATTAAGACCCATTTTATCTAC
644R_Cmax_pum_Phi     GCCTTCTTGATTTAACCGGACAAAGAATGGTGAATATATCTTACTAGTAGTGTGTTTCTCTGTGTGTGTGTGTGTGTGTGTGTGT------AATCTTTGGAGAACTCTCTCATTGATTAAGACCCATTTTATCTAC

TGATCTGATTTTCAGCCAACAATGATGAAGTTCGGAGCA
TGATCTGATTTTCAGCCAACAATGATGAAGTTCGGAGCA
TGATCTGATTTTCAGCCAACAATGATGAAGTTCGGAGCA
TGATCTGATTTTCAGCCAACAATGATGAAGTTCGGAGCA
TGATCTGATTTTCAGCCAACAATGATGAAGTTCGGAGCA
TGATCTGATTTTCAGCCAACAATGATGAAGTTCGGAGCA
TGATCTGATTTTCAGCCAACAATGATGAAGTTCGGAGCA
TGATCTGATTTTCAGCCAACAATGATGAAGTTCGGAGCA
TGATCTGATTTTCAGCCAACAATGATGAAGTACGGAGCA
TGATCTGATTTTCAGCCAACAATGATGAAGTACGGAGCA
TGATCTGATTTTCAGCCAACAATGATGAAGTTCGGAGCA
TGATCTGATTTTCAGCCAACAATGATGAAGTTCGGAGCA
TGATCTGATTTTCAGCCAACAATGATGAAGTTCGGAGCA
TGATCTGATTTTCAGCCAACAATGATGAAGTTCGGAGCA
TGATCTGATTTTCAGCCAACAATGATGAAGTTCGGAGCA
TGATCTGATTTTCAGCCAACAATGATGAAGTTCGGAGCA
TGATCTGATTTTCAGCCAACAATGATGAAGTTCGGAGCA
TGATCTGATTTTCAGCCAACAATGATGAAGTTCGGAGCA
```

```
TGATCTGATTTTCAGCCAACAATGATGAAGTTCGGAGCA
TGATCTGATTTTCAGCCAACAATGATGAAGTTCGGAGCA
TGATCTGATTTTCAGCCAACAATGATGAAGTTCGGAGCA
TGATCTGATTTTCAGCCAACAATGATGAAGTTCGGAGCA
TGATCTGATTTTCAGCCAACAATGATGAAGTTCGGAGCA
TGATCTGATTTTCAGCCAACAATGATGAAGTTCGGAGCA
TGATCTGATTTTCAGCCAACAATGATGAAGTTCGGAGCA
TGATCTGATTTTCAGCCAACAATGATGAAGTTCGGAGCA
TGATCTGATTTTCAGCCAACAATGATGGAGTTCGGAGCA
TGATCTGATTTTCAGCCAACAATGATGGAGTTCGGAGCA
TGATCTGATTTTCAGCCAACAATGATGAAGTTCGGAGCA
TGATCTGATTTTCAGCCAACAATGATGAAGTTCGGAGCA
TGATCTGATTTTCAGCCAACAATGATGAAGTTCGGAGCA
TGATCTGATTTTCAGCCAACAATGATGAAGTTCGGAGCA
TGATCTGATTTTCAGCCAACAATGATGAAGTTCGGAGCA
TGATCTGATTTTCAGCCAACAATGATGAAGTTCGGAGCA
TGATCTGATTTTCAGCCAACAATGATGAAGTTCGGAGCA
TGATCTGATTTTCAGCCAACAATGATGAAGTTCGGAGCA
```

Jc3A10

```
p123_Coun_1    GCTCTGCTTTCCATTTCTCAGTTCTCACACACCACACACGCTGACCGCCAGAGAGAGATCTGGTAATTGTTCCACCAAACAATAAGCTCCACAAACACAAAACACACCCGCA--CTCTCTCTCTCTCTCTCTCTCTCTCT----
p123_Coun_2    GCTCTGCTTTCCATTTCTCAGTTCTCACACACCACACACGCTGACCGCCAGAGAGAGATCTGGTAATTGTTCCACCAAACAATAAGCTCCACAAACACAAAACACACCCGCA--CTCTCTCTCTCTCTCTCTCTCTCTCT----
p186_Coun_1    GCTCTGCTTTCCATTTCTCAGTTCTCACACACCACACACGCTGACCGCCAGAGAGAGATCTGGTAATTGTTCCACCAAACAATAAGCTCCACAAACACAAAACACACCCGCA--CTCTCTCTCTCTCTCT--------------
p186_Coun_2    GCTCTGCTTTCCATTTCTCAGTTCTCACACACCACACACGCTGACCGCCAGAGAGAGATCTGGTAATTGTTCCACCAAACAATAAGCTCCACAAACACAAAACACACCCGCA—CTCTCTCTCTCTCTCTCTCTCTCTCTC--
p188_Coun_1    GCTCTGCTTTCCATTTCTCAGTTCTCACACACCACACACGCTGACCGCCAGAGAGAGATCTGGTAATTGTTCCACCAAACAATAAGCTCCACAAACACAAAACACACCCGCA--CTCTCTCTCTCTCTCT--------------
p188_Coun_2    GCTCTGCTTTCCATTTCTCAGTTCTCACACACCACACACGCTGACCGCCAGAGAGAGATCTGGTAATTGTTCCACCAAACAATAAGCTCCACAAACACAAAACACACCCGCA--CTCTCTCTCT--------------------
p236_Coun_1    GCTCTGCTTTCCATTTCTCAGTTCTCACCCACCACACACGCTGACCGCCAGAGAGAGATCTGGTAATTGTTCCACCAAACAATAAGCTCCACAAACACAAAACACACCCGCA--CTCTCTCTCTCTCTCTCTCT----------
p236_Coun_2    GCTCTGCTTTCCATTTCTCAGTTCTCACCCACCACACACGCTGACCGCCAGAGAGAGATCTGGTAATTGTTCCACCAAACAATAAGCTCCACAAACACAAAACACACCCGCA--CTCTCTCTCTCTCTCTCTCTCT--------
p269_Coun_1    GCTCTGCTTTCCATTTCTCAGTTCTCACACACCACACACGCTGACCGCCAGAGAGAGATCTGGTAATTGTTCCACCAAACAATAAGCTCCACAAACACAAAACACACCCGCA--CTCTCTCTCTCTCTCTCT------------
p269_Coun_2    GCTCTGCTTTCCATTTCTCAGTTCTCACACACCACACACGCTGACCGCCAGAGAGAGATCTGGTAATTGTTCCACCAAACAATAAGCTCCACAAACACAAAACACACCCGCA--CTCTCTCTCTCTCTCTCTCTCT--------
p277_Coun_1    GCTCTGCTTTCCATTTCTCAGTTCTCACACACCACACACGCTGACCGCCAGAGAGAGATCTGGTAATTGTTCCACCAAACAATAAGCTCCACAAACACAAAACACACCCGCA--CTCTCTCTCTCTCTCT--------------
p277_Coun_2    GCTCTGCTTTCCATTTCTCAGTTCTCACACACCACACACGCTGACCGCCAGAGAGAGATCTGGTAATTGTTCCACCAAACAATAAGCTCCACAAACACAAAACACACCCGCA--CTCTCTCTCTCTCTCTCTCT----------
p305_Coun_1    GCTCTGCTTTCCATTTCTCAGTTCTCACACACCACACACGCTGACCGCCAGAGAGAGATCTGGTAATTGTTCCACCAAACAATAAGCTCCACAAACACAAAACACACCCGCA--CTCTCTCTCTCTCTCT--------------
p305_Coun_2    GCTCTGCTTTCCATTTCTCAGTTCTCACACACCACACACGCTGACCGCCAGAGAGAGATCTGGTAATTGTTCCACCAAACAATAAGCTCCACAAACACAAAACACACCCGCA--CTCTCTCTCTCTCTCT--------------
p307_Coun_1    GCTCTGCTTTCCATTTCTCAGTTCTCACACACCACACACGCTGACCGCCAGAGAGAGATCTGGTAATTGTTCCACCAAACAATAAGCTCCACAAACACAAAACACACCCGCA--CTCTCTCTCTCTCTCTCT------------
p307_Coun_2    GCTCTGCTTTCCATTTCTCAGTTCTCACACACCACACACGCTGACCGCCAGAGAGAGATCTGGTAATTGTTCCACCAAACAATAAGCTCCACAAACACAAAACACACCCGCA--CTCTCTCTCTCTCTCTCTCTCTCT------
p337_Coun_1    GCTCTGCTTTCCATTTCTCAGTTCTCACACACCACACACGCTGACCGCCAGAGAGAGATCTGGTAATTGTTCCACCAAACAATAAGCTCCACAAACACAAAACACACCCGCA--CTCTCTCTCTCTCTCTCT------------
p337_Coun_2    GCTCTGCTTTCCATTTCTCAGTTCTCACACACCACACACGCTGACCGCCAGAGAGAGATCTGGTAATTGTTCCACCAAACAATAAGCTCCACAAACACAAAACACACCCGCA--CTCTCTCTCTCTCTCTCTCTCT--------
p339_Coun_1    GCTCTGCTTTCCATTTCTCAGTTCTCACACACCACACACGCTGACCGCCAGAGAGAGATCTGGTAATTGTTCCACCAAACAATAAGCTCCACAAACACAAAACACACCCGCA--CTCTCTCTCTCTCT----------------
p339_Coun_2    GCTCTGCTTTCCATTTCTCAGTTCTCACACACCACACACGCTGACCGCCAGAGAGAGATCTGGTAATTGTTCCACCAAACAATAAGCTCCACAAACACAAAACACACCCGCA--CTCTCTCTCTCTCTCT--------------
p340_Coun_1    GCTCTGCTTTCCATTTCTCAGTTCTCACACACCACACACGCTGACCGCCAGAGAGAGATCTGGTAATTGTTCCACCAAACAATAAGCTCCACAAACACAAAACACACCCGCA--CTCTCTCTCTCTCTCTCT------------
p340_Coun_2    GCTCTGCTTTCCATTTCTCAGTTCTCACACACCACACACGCTGACCGCCAGAGAGAGATCTGGTAATTGTTCCACCAAACAATAAGCTCCACAAACACAAAACACACCCGCA--CTCTCTCTCTCTCTCTCTCTCTCTCT----
p345_Coun_1    GCTCTGCTTTCCATTTCTCAGTTCTCACACACCACACACGCTGACCGCCAGAGAGAGATCTGGTAATTGTTCCACCAAACAATAAGCTCCACAAACACAAAACACACCCGCA--CTCTCTCTCTCTCTCT--------------
p345_Coun_2    GCTCTGCTTTCCATTTCTCAGTTCTCACACACCACACACGCTGACCGCCAGAGAGAGATCTGGTAATTGTTCCACCAAACAATAAGCTCCACAAACACAAAACACACCCGCA--CTCTCTCTCTCTCTCT--------------
p441_Coun_1    GCTCTGCTTTCCATTTCTCAGTTCTCACACACCACACACGCTGACCGCCAGAGAGAGATCTGGTAATTGTTCCACCAAACAATAAGCTCCACAAACACAAAACACACCCGCA--CTCTCTCTCTCTCTCT--------------
p441_Coun_2    GCTCTGCTTTCCATTTCTCAGTTCTCACACACCACACACGCTGACCGCCAGAGAGAGATCTGGTAATTGTTCCACCAAACAATAAGCTCCACAAACACAAAACACACCCGCA--CTCTCTCTCTCTCTCTCTCTCTCTCT----
p470_Coun_1    GCTCTGCTTTCCATTTCTCAGTTCTCACACACCACACACGCTGACCGCCAGAGAGAGATCTGGTAATTGTTCCACCAAACAATAAGCTCCACAAACACAAAACACACCCGCA--CTCTCTCTCTCTCTCTCTCT----------
p470_Coun_2    GCTCTGCTTTCCATTTCTCAGTTCTCACACACCACACACGCTGACCGCCAGAGAGAGATCTGGTAATTGTTCCACCAAACAATAAGCTCCACAAACACAAAACACACCCGCA--CTCTCTCTCTCTCTCT--------------
p495_Coun_1    GCTCTGCTTTCCATTTCTCAGTTCTCACACACCACACACGCTGACCGCCAGAGAGAGATCTGGTAATTGTTCCACCAAACAATAAGCTCCACAAACACAAAACACACCCGCA--CTCTCTCTCTCTCTCT--------------
p495_Coun_2    GCTCTGCTTTCCATTTCTCAGTTCTCACACACCACACACGCTGACCGCCAGAGAGAGATCTGGTAATTGTTCCACCAAACAATAAGCTCCACAAACACAAAACACACCCGCA--CTCTCTCTCTCTCTCT--------------
F1032_Para_1   GCTCTGCTTTCCATTTCTCAGTTCTCACACACCACACACGCTGACCGCCAGAGAGAGATCTGGTAATTGTTCCACCAAACAATAAGCTCCACAAACACAAAACACACCCGCA--CTCTCTCTCTCTCTCT--------------
F1032_Para_2   GCTCTGCTTTCCATTTCTCAGTTCTCACACACCACACACGCTGACCGCCAGAGAGAGATCTGGTAATTGTTCCACCAAACAATAAGCTCCACAAACACAAAACACACCCGCA--CTCTCTCTCTCTCTCTCTCTCTCTCTCTCT
F1038_Para_1   GCTCTGCTTTCCATTTCTCAGTTCTCACACACCACACACGCTGACCGCCAGAGAGAGATCTGGTAATTGTTCCACCAAACAATAAGCTCCACAAACACAAAACACACCCGCA--CTCTCTCTCTCTCTCT--------------
F1038_Para_2   GCTCTGCTTTCCATTTCTCAGTTCTCACACACCACACACGCTGACCGCCAGAGAGAGATCTGGTAATTGTTCCACCAAACAATAAGCTCCACAAACACAAAACACACCCGCA--CTCTCTCTCTCTCTCTCTCTCTCTCTCTCT
F810_Para_1    GCTCTGCTTTCCATTTCTCAGTTCTCACACACCACACACGCTGACCGCCAGAGAGAGATCTGGTAATTGTTCCACCAAACAATAAGCTCCACAAACACAAAACACACCCGCA--CTCTCTCTCTCTCTCTCT------------
```

```
F810_Para_2    GCTCTGCTTTCCATTTCTCAGTTCTCACACACCACACACGCTGACCGCCAGAGAGAGATCTGGTAATTGTTCCACCAAACAATAAGCTCCACAAACACAAAACACACCCGCA--CTCTCTCTCTCTCTCT--------------
F826_Para_1    GCTCTGCTTTCCATTTCTCAGTTCTCACACACCACACACGCTGACCGCCAGAGAGAGATCTGGTAATTGTTCCACCAAACAATAAGCTCCACAAACACAAAACACACCCGCA--CTCTCTCTCTCTCTCT--------------
F826_Para_2    GCTCTGCTTTCCATTTCTCAGTTCTCACACACCACACACGCTGACCGCCAGAGAGAGATCTGGTAATTGTTCCACCAAACAATAAGCTCCACAAACACAAAACACACCCGCA--CTCTCTCTCTCTCTCT--------------
F850_Para_1    GCTCTGCTTTCCATTTCTCAGTTCTCACACACCACACACGCTGACCGCCAGAGAGAGATCTGGTAATTGTTCCACCAAACAATAAGCTCCACAAACACAAAACACACCCGCA--CTCTCTCTCTCTCTCTCTCTCTCTCTCTCT
F850_Para_2    GCTCTGCTTTCCATTTCTCAGTTCTCACACACCACACACGCTGACCGCCAGAGAGAGATCTGGTAATTGTTCCACCAAACAATAAGCTCCACAAACACAAAACACACCCGCA--CTCTCTCTCTCTCTCTCTCTCTCTCTCT—
F852_Para_1    GCTCTGCTTTCCATTTCTCAGTTCTCACACACCACACACGCTGACCGCCAGAGAGAGATCTGGTAATTGTTCCACCAAACAATAAGCTCCACAAACACAAAACACACCCGCA--CTCTCTCTCTCTCTCTCTCTCT--------
F852_Para_2    GCTCTGCTTTCCATTTCTCAGTTCTCACACACCACACACGCTGACCGCCAGAGAGAGATCTGGTAATTGTTCCACCAAACAATAAGCTCCACAAACACAAAACACACCCGCA--CTCTCTCTCTCTCTCTCTCTCTCTCTCTCT
F885_Para_1    GCTCTGCTTTCCATTTCTCAGTTCTCACACACCACACACGCTGACCGCCAGAGAGAGATCTGGTAATTGTTCCACCAAACAATAAGCTCCACAAACACAAAACACACCCGCA--CTCTCTCTCTCTCTCTCT------------
F885_Para_2    GCTCTGCTTTCCATTTCTCAGTTCTCACACACCACACACGCTGACCGCCAGAGAGAGATCTGGTAATTGTTCCACCAAACAATAAGCTCCACAAACACAAAACACACCCGCA--CTCTCTCTCTCTCTCT--------------
F896_Para_1    GCTCTGCTTTCCATTTCTCAGTTCTCACACACCACACACGCTGACCGCCAGAGAGAGATCTGGTAATTGTTCCACCAAACAATAAGCTCCACAAACACAAAACACACCCGCA--CTCTCTCTCTCTCTCTCT------------
F896_Para_2    GCTCTGCTTTCCATTTCTCAGTTCTCACACACCACACACGCTGACCGCCAGAGAGAGATCTGGTAATTGTTCCACCAAACAATAAGCTCCACAAACACAAAACACACCCGCA--CTCTCTCTCTCTCTCT--------------
F946_Para_1    GCTCTGCTTTCCATTTCTCAGTTCTCACACACCACACACGCTGACCGCCAGAGAGAGATCTGGTAATTGTTCCACCAAACAATAAGCTCCACAAACACAAAACACACCCGCA--CTCTCTCTCTCTCTCTCTCTCTCTCT----
F946_Para_2    GCTCTGCTTTCCATTTCTCAGTTCTCACACACCACACACGCTGACCGCCAGAGAGAGATCTGGTAATTGTTCCACCAAACAATAAGCTCCACAAACACAAAACACACCCGCA--CTCTCTCTCTCTCTCTCT------------
F960_Para_1    GCTCTGCTTTCCATTTCTCAGTTCTCACACACCACACACGCTGACCGCCAGAGAGAGATCTGGTAATTGTTCCACCAAACAATAAGCTCCACAAACACAAAACACACCCGCA--CTCTCTCTCTCTCTCT--------------
F960_Para_2    GCTCTGCTTTCCATTTCTCAGTTCTCACACACCACACACGCTGACCGCCAGAGAGAGATCTGGTAATTGTTCCACCAAACAATAAGCTCCACAAACACAAAACACACCCGCA--CTCTCTCTCTCTCTCT--------------
T308_Para_1    GCTCTGCTTTCCATTTCTCAGTTCTCACACACCACACACGCTGACCGCCAGAGAGAGATCTGGTAATTGTTCCACCAAACAATAAGCTCCACAAACACAAAACACACCCGCA--CTCTCTCTCTCTCTCTCTCT----------
T308_Para_2    GCTCTGCTTTCCATTTCTCAGTTCTCACACACCACACACGCTGACCGCCAGAGAGAGATCTGGTAATTGTTCCACCAAACAATAAGCTCCACAAACACAAAACACACCCGCA--CTCTCTCTCTCTCTCTCT------------
T316_Para_1    GCTCTGCTTTCCATTTCTCAGTTCTCACACACCACACACGCTGACCGCCAGAGAGAGATCTGGTAATTGTTCCACCAAACAATAAGCTCCACAAACACAAAACACACCCGCA--CTCTCTCTCTCTCTCT--------------
T316_Para_2    GCTCTGCTTTCCATTTCTCAGTTCTCACACACCACACACGCTGACCGCCAGAGAGAGATCTGGTAATTGTTCCACCAAACAATAAGCTCCACAAACACAAAACACACCCGCA--CTCTCTCTCTCTCTCT--------------
T348_Para_1    GCTCTGCTTTCCATTTCTCAGTTCTCACACACCACACACGCTGACCGCCAGAGAGAGATCTGGTAATTGTTCCACCAAACAATAAGCTCCACAAACACAAAACACACCCGCA--CTCTCTCTCTCTCTCTCT------------
T348_Para_2    GCTCTGCTTTCCATTTCTCAGTTCTCACACACCACACACGCTGACCGCCAGAGAGAGATCTGGTAATTGTTCCACCAAACAATAAGCTCCACAAACACAAAACACACCCGCA--CTCTCTCTCTCTCTCT--------------
T350_Para_1    GCTCTGCTTTCCATTTCTCAGTTCTCACACACCACACACGCTGACCGCCAGAGAGAGATCTGGTAATTGTTCCACCAAACAATAAGCTCCACAAACACAAAACACACCCGCA--CTCTCTCTCTCTCTCTCT------------
T350_Para_2    GCTCTGCTTTCCATTTCTCAGTTCTCACACACCACACACGCTGACCGCCAGAGAGAGATCTGGTAATTGTTCCACCAAACAATAAGCTCCACAAACACAAAACACACCCGCA--CTCTCTCTCTCTCTCTCTCT----------
T352_Para_1    GCTCTGCTTTCCATTTCTCAGTTCTCACACACCACACACGCTGACCGCCAGAGAGAGATCTGGTAATTGTTCCACCAAACAATAAGCTCCACAAACACAAAACACACCCGCA--CTCTCTCTCTCTCTCT--------------
T352_Para_2    GCTCTGCTTTCCATTTCTCAGTTCTCACACACCACACACGCTGACCGCCAGAGAGAGATCTGGTAATTGTTCCACCAAACAATAAGCTCCACAAACACAAAACACACCCGCA--CTCTCTCTCTCTCTCTTCT-----------
T354_Para_1    GCTCTGCTTTCCATTTCTCAGTTCTCACACACCACACACGCTGACCGCCAGAGAGAGATCTGGTAATTGTTCCACCAAACAATAAGCTCCACAAACACAAAACACACCCGCA--CTCTCTCTCTCTCTCTCT------------
T354_Para_2    GCTCTGCTTTCCATTTCTCAGTTCTCACCCACCACACACGCTGACCGCCAGAGAGAGATCTGGTAATTGTTCCACCAAACAATAAGCTCCACAAACACAAAACACACCCGCA--CTCTCTCTCTCTCTCT--------------
T356_Para_1    GCTCTGCTTTCCATTTCTCAGTTCTCACACACCACACACGCTGACCGCCAGAGAGAGATCTGGTAATTGTTCCACCAAACAATAAGCTCCACAAACACAAAACACACCCGCA--CTCTCTCTCTCTCTCT--------------
T356_Para_2    GCTCTGCTTTCCATTTCTCAGTTCTCACACACCACACACGCTGACCGCCAGAGAGAGATCTGGTAATTGTTCCACCAAACAATAAGCTCCACAAACACAAAACACACCCGCA--CTCTCTCTCTCTCTCT--------------
T358_Para_1    GCTCTGCTTTCCATTTCTCAGTTCTCACACACCACACACGCTGACCGCCAGAGAGAGATCTGGTAATTGTTCCACCAAACAATAAGCTCCACAAACACAAAACACACCCGCA--CTCTCTCTCTCTCT----------------
T358_Para_2    GCTCTGCTTTCCATTTCTCAGTTCTCACACACCACACACGCTGACCGCCAGAGAGAGATCTGGTAATTGTTCCACCAAACAATAAGCTCCACAAACACAAAACACACCCGCA--CTCTCTCTCTCTCTCT--------------
T360_Para_1    GCTCTGCTTTCCATTTCTCAGTTCTCACACACCACACACGCTGACCGCCAGAGAGAGATCTGGTAATTGTTCCACCAAACAATAAGCTCCACAAACACAAAACACACCCGCA--CTCTCTCTCTCTCTCTCT------------
T360_Para_2    GCTCTGCTTTCCATTTCTCAGTTCTCACACACCACACACGCTGACCGCCAGAGAGAGATCTGGTAATTGTTCCACCAAACAATAAGCTCCACAAACACAAAACACACCCGCA--CTCTCTCTCTCTCTCTCT------------
T366_Para_1    GCTCTGCTTTCCATTTCTCAGTTCTCACACACCACACACGCTGACCGCCAGAGAGAGATCTGGTAATTGTTCCACCAAACAATAAGCTCCACAAACACAAAACACACCCGCA--CTCTCTCTCTCTCTCTCTCTCTCTCTCTCT
T366_Para_2    GCTCTGCTTTCCATTTCTCAGTTCTCACACACCACACACGCTGACCGCCAGAGAGAGATCTGGTAATTGTTCCACCAAACAATAAGCTCCACAAACACAAAACACACCCGCA--CTCTCTCTCTCTCTCTCT------------
T372_Para_1    GCTCTGCTTTCCATTTCTCAGTTCTCACACACCACACACGCTGACCGCCAGAGAGAGATCTGGTAATTGTTCCACCAAACAATAAGCTCCACAAACACAAAACACACCCGCA--CTCTCTCTCTCTCTCTCT------------
T372_Para_2    GCTCTGCTTTCCATTTCTCAGTTCTCACACACCACACACGCTGACCGCCAGAGAGAGATCTGGTAATTGTTCCACCAAACAATAAGCTCCACAAACACAAAACACACCCGCA--CTCTCTCTCTCTCTCT--------------
T386_Para_1    GCTCTGCTTTCCATTTCTCAGTTCTCACACACCACACACGCTGACCGCCAGAGAGAGATCTGGTAATTGTTCCACCAAACAATAAGCTCCACAAACACAAAACACACCCGCA--CTCTCTCTCTCTCTCT--------------
T386_Para_2    GCTCTGCTTTCCATTTCTCAGTTCTCACACACCACACACGCTGACCGCCAGAGAGAGATCTGGTAATTGTTCCACCAAACAATAAGCTCCACAAACACAAAACACACCCGCA--CTCTCTCTCTCTCTCT--------------
T430_Para_1    GCTCTGCTTTCCATTTCTCAGTTCTCACACACCACACACGCTGACCGCCAGAGAGAGATCTGGTAATTGTTCCACCAAACAATAAGCTCCACAAACACAAAACACACCCGCA--CTCTCTCTCTCTCTCTCTCT----------
T430_Para_2    GCTCTGCTTTCCATTTCTCAGTTCTCACACACCACACACGCTGACCGCCAGAGAGAGATCTGGTAATTGTTCCACCAAACAATAAGCTCCACAAACACAAAACACACCCGCA--CTCTCTCTCTCTCTCTCT------------
T432_Para_1    GCTCTGCTTTCCATTTCTCAGTTCTCACACACCACACACGCTGACCGCCAGAGAGAGATCTGGTAATTGTTCCACCAAACAATAAGCTCCACAAACACAAAACACACCCGCA--CTCTCTCTTTCTCTCTCT------------
T432_Para_2    GCTCTGCTTTCCATTTCTCAGTTCTCACACACCACACACGCTGACCGCCAGAGAGAGATCTGGTAATTGTTCCACCAAACAATAAGCTCCACAAACACAAAACACACCCGCA--CTCTCTCTCTCTCTCTCT------------
C733_Ouest_1   GCTCTGCTTTCCATTTCTCAGTTCTCACACACCACACACGCTGACCGCCAGAGAGAGATCTGGTAATTGTTCCACCAAACAATAAGCTCCACAAACACAAAACACACCCGCA--CTCTCTCTCTCTCTCT--------------
C733_Ouest_2   GCTCTGCTTTCCATTTCTCAGTTCTCACACACCACACACGCTGACCGCCAGAGAGAGATCTGGTAATTGTTCCACCAAACAAGAAGCTCCACAAACACAAAACACACCCGCA--CTCTCTCTCTCTCTCT--------------
C735_Ouest_1   GCTCTGCTTTCCATTTCTCAGTTCTCACACACCACACACGCTGACCGCCAGAGAGAGATCTGGTAATTGTTCCACCAAACAAGAAGCTCCACAAACACAAAACACACCCGCA--CTCTCTCTCTCTCTCT--------------
C735_Ouest_2   GCTCTGCTTTCCATTTCTCAGTTCTCACACACCACACACGCTGACCGCCAGAGAGAGATCTGGTAATTGTTCCACCAAACAATAAGCTCCACAAACACAAAACACACCCGCA--CTCTCTCTCTCTCTCTCT------------
C737_Ouest_1   GCTCTGCTTTCCATTTCTCAGTTCTCACACACCACACACGCTGACCGCCAGAGAGAGATCTGGTAATTGTTCCACCAAACAAGAAGCTCCACAAACACAAAACACACCCGCA--CTCTCTCTCTCTCTCTCT------------
C737_Ouest_2   GCTCTGCTTTCCATTTCTCAGTTCTCACACACCACACACGCTGACCGCCAGAGAGAGATCTGGTAATTGTTCCACCAAACAAGAAGCTCCACAAACACAAAACACACCCGCA--CTCTCTCTCTCTCTCT--------------
C913_Ouest_1   GCTCTGCTTTCCATTTCTCAGTTCTCACACACCACACACGCTGACCGCCAGAGAGAGATCTGGTAATTGTTCCACCAAACAATAAGCTCCACAAACACAAAACACACCCGCA--CTCTCTCTCTCTCTCTCTCT----------
C913_Ouest_2   GCTCTGCTTTCCATTTCTCAGTTCTCACACACCACACACGCTGACCGCCAGAGAGAGATATGGTAATTGTTCCACCAAACAATAAGCTCCACAAACACAAAACACACCCGCA--CTCTCTCTCTCTCTCTCTCTCT--------
C916_Ouest_1   GCTCTGCTTTCCATTTCTCAGTTCTCACACACCACACACGCTGACCGCCAGAGAGAGATCTGGTAATTGTTCCACCAAACAATAAGCTCCACAAACACAAAACACACCCGCACACTCTCTCTCTCTCTCTCTCT----------
C916_Ouest_2   GCTCTGCTTTCCATTTCTCAGTTCTCACACACCACACACGCTGACCGCCAGAGAGAGATCTGGTAATTGTTCCACCAAACAATAAGCTCCACAAACACAAAACACACCCGCACACTCTCTCTCTCTCTCTCTCT----------
S263_Ouest_1   GCTCTGCTTTCCATTTCTCAGTTCTCACACACCACACACGCTGACCGCCAGAGAGAGATATGGTAATTGTTCCACCAAACAATAAGCTCCACAAACACAAAACACACCCGCACACTCTCTCTCTCTCTCTCTCT----------
S263_Ouest_2   GCTCTGCTTTCCATTTCTCAGTTCTCACACACCACACACGCTGACCGCCAGAGAGAGATCTGGTAATTGTTCCACCAAACAATAAGCTCCACAAACACAAAACACACCCGCACACTCTCTCTCTCTCTCTCTCT----------
S264_Ouest_1   GCTCTGCTTTCCATTTCTCAGTTCTCACACACCACACACGCTGACCGCCAGAGAGAGATCTGGTAATTGTTCCACCAAACAAGAAGCTCCACAAACACAAAACACACCCGCACACTCTCTCTCTCTCTCTCT------------
S264_Ouest_2   GCTCTGCTTTCCATTTCTCAGTTCTCACACACCACACACGCTGACCGCCAGAGAGAGATCTGGTAATTGTTCCACCAAACAATAAGCTCCACAAACACAAAACACACCCGCACACTCTCTCTCTCTCTCTCTCT----------
```

```
S274_Ouest_1   GCTCTGCTTTCCATTTCTCAGTTCTCACACACCACACACGCTGACCGCCAGAGAGAGATCTGGTAATTGTTCCACCAAACAATAAGCTCCACAAACACAAAACACACCCGCA--CTCTCTCTCTCTCTCTCT------------
S274_Ouest_2   GCTCTGCTTTCCATTTCTCAGTTCTCACACACCACACACGCTGACCGCCAGAGAGAGATCTGGTAATTGTTCCACCAAACAATAAGCTCCACAAACACAAAACACACCCGCA--CTCTCTCTCTCTCTCTCT------------
OUT198_Bre_1   GCTCTGCTTTCCATTTCTCAGTTCTCACACACCACACACGCTGACCGCCAGAGAGAGATCTGGTAATTGTTCCACCAAACAATAAGCTCCACAAACACAAAACACACCCGCACACTCTCTCTCTCTCTCTCT------------
OUT198_Bre_2   GCTCTGCTTTCCATTTCTCAGTTCTCACACACCACACACGCTGACCGCCAGAGAGAGATCTGGTAATTGTTCCACCAAACAAGAAGCTCCACAAACACAAAACACACCCGCACACTCTCTCTCTCTCTCTCTCT----------
OUT204_Bre_1   GCTCTGCTTTCCATTTCTCAGTTCTCACACACCACACACGCTGACCGCCAGAGAGAGATCTGGTAATTGTTCCACCAAACAATAAGCTCCACAAACACAAAACACACCCGCA--CTCTCTCTCTCTCTCTCT------------
OUT204_Bre_2   GCTCTGCTTTCCATTTCTCAGTTCTCACACACCACACACGCTGACCGCCAGAGAGAGATCTGGTAATTGTTCCACCAAACAAGAAGCTCCACAAACACAAAACACACCCGCACACTCTCTCTCTCTCTCTCTCTCT--------
OUT213_Bre_1   GCTCTGCTTTCCATTTCTCAGTTCTCACACACCACACACGCTGACCGCCAGAGAGAGATCTGGTAATTGTTCCACCAAACAATAAGCTCCACAAACACAAAACACACCCGCA--CTCTCTCTCTCTCTCTCT------------
OUT213_Bre_2   GCTCTGCTTTCCATTTCTCAGTTCTCACACACCACACACGCTGACCGCCAGAGAGAGATCTGGTAATTGTTCCACCAAACAATAAGCTCCACAAACACAAAACACACCCGCACACTCTCTCTCTCTCTCTCTCT----------

----------CACACACACACACA----------TAG
----------CACACACACACACACACA------TAG
----------CACACACACACACACA--------TAG
----------CACACACACACACA----------TAG
----------CACACACACACACACACA------TAG
----------CACACACACACACACA--------TAG
----------CACACACACACACACA--------TAG
----------CACACACACACACA----------TAG
----------CACACACACACACACA--------TAG
----------CACACACACACACA----------TAG
----------CACACACACACACA----------TAG
----------CACACACACACACACA--------TAG
----------CACACACACACACACACA------TAG
----------CACACACACACACACACA------TAG
----------CACACACACACACACACACA----TAG
----------CACACACACACACACA--------TAG
----------CACACACACACACACACA------TAG
----------CACACACACACACACA--------TAG
----------CACACACACACACACA--------TAG
----------CACACACACACACACA--------TAG
----------CACACACACACACACACA------TAG
----------CACACACACACACA----------TAG
----------CACACACACACACACA--------TAG
----------CACACACACACACACA--------TAG
----------CACACACACACACACA--------TAG
----------CACACACACACACACA--------TAG
----------CACACACACACACACA--------TAG
----------CACACACACACACA----------TAG
----------CACACACACACACACACA------TAG
----------CACACACACACACACACA------TAG
----------CACACACACACACACA--------TAG
----------CACACACACACACACACACA----TAG
----------CACACACACACACACA--------TAG
CTCTCTCTCTCACACACACACACACACA------TAG
----------CACACACACACACACACA------TAG
----------CACACACACACACACA--------TAG
----------CACACACACACACACA--------TAG
----------CACACACACACACACACACACA—TAG
----------CACACACACACACACACA------TAG
----------CACACACACACACACA--------TAG
----------CACACACACACACACA--------TAG
----------CACACACACACACA----------TAG
----------CACACACACACACACACACA----TAG
----------CACACACACACACACA--------TAG
----------CACACACACACACACA--------TAG
----------CACACACACACACACA--------TAG
----------CACACACACACACA----------TAG
----------CACACACACACACACA--------TAG
```

----------CACACACACACACACACA------TAG
----------CACACACACACACACACA------TAG
----------CACACACACACACACA--------TAG
----------CACACACACACACACACA------TAG
----------CACACACACACACACA--------TAG
----------CACACACACACACACA--------TAG
----------CACACACACACACACACACA----TAG
----------CACACACACACACACA--------TAG
----------CACACACACACACACA--------TAG
----------CACACACACACACACACA------TAG
----------CACACACACACACACA--------TAG
----------CACACACACACACACA--------TAG
----------CACACACACACACACA--------TAG
----------CACACACACACACACACACA----TAG
----------CACACACACACACACA--------TAG
----------CACACACACACACACA--------TAG
----------CACACACACACACACACA------TAG
----------CACACACACACACACA--------TAG
----------CACACACACACACACACACA----TAG
----------CACACACACACACACACACA----TAG
----------CACACACACACACACACA------TAG
----------CACACACACACACACA--------TAG
----------CACACACACACACACA--------TAG
----------CACACACACACACACA--------TAG
----------CACACACACACACACACA------TAG
----------CACACACACACACACA--------TAG
----------CACACACACACACACA--------TAG
----------CACACACACACACACACACA----TAG
----------CACACACACACACACACA------TAG
----------CACACACACACACACA--------TAG
----------CACACACACACACA----------TAG
----------CACACACACACACACA--------TAG
----------CACACACACACACACA--------TAG
----------CACACACACACACACACACA----TAG
----------CACACACACACACACACA------TAG
----------CACACACACACACACA--------TAG
----------CACACACACACACACACACACACACATAG
----------CACACACTCACACACACACACA--TAG
----------CACACACACACACACACACACACACATAG
----------CACACACACACACACA--------TAG
----------CACACACACACACACACA------TAG
----------CACACACACACACACACA------TAG
----------CACACACACACACACACA------TAG
----------CACACACACACACA----------TAG
----------CACACACACACACACACACA----TAG
----------CACACACACACACACACACA----TAG
----------CACACACACACACACA--------TAG
----------CACACACACACACACA--------TAG
----------CACACACACACACACACACACA--TAG
----------CACACACACACACACACACACA--TAG
----------CACACACACACACACACA------TAG
----------CACACACACACACACACA------TAG

Jc3F4

```
p186_Coun_1   ACAC-GTAACCTCCTCCTTCCATTACTCCTCTAACTCTTCCCCCACACCTCACTGCACTTAAAATGCGCAGTTGCGCACTCGCTGTGTGTGCTTATATGT--GAGAGAGAGAGAGAGAGA----------------------TTG
p186_Coun_2   ACAC-GTAACCTCCTCCTTCCATTACTCCTCTAACTCTTCCCCCACACCTCACTGCACTTAAAATGCGCAGTTGCGCACTCGCTGTGTGTGCTTATATGT--GAGAGAGAGAGAGAGAGAGAGAGAGAGAGAGAGAGAGAGATTG
p188_Coun_1   ACAC-GTAACCTCCTCCTTCCATTACTCCTCTAACTCTTCCCCCACACCTCACTGCACTTAAAATGCGCAGTTGCGCACTCGCTGTGTGTGCTTATATGT--GAGAGAGAGAGA----------------------------TTG
p188_Coun_2   ACAC-GTAACCTCCTCCTTCCATTACTCCTCTAACTCTTCCCCCAAACCTCACTGCACTTAAAATGCGCAGTTGCGCACTCGCTGTGTGTGCTTATATGT--GAGAGAGAGAGA----------------------------TTG
p216_Coun_1   ACAC-GTAACCTCCTCCTTCCATTACTCCTCTAACTCTTCCCCCACACCTCACTGCACTTAAAATGCGCAGTTGCGCACTCGCTGTGTGTGCTTATATGT--GAGAGAGAGAGA----------------------------TTG
p216_Coun_2   ACAC-GTAACCTCCTCCTTCCATTACTCCTCTAACTCTTCCCCCACACCTCACTGCACTTAAAATGCGCAGTTGCGCACTCGCTGTGTGTGCTTATATGT--GAGAGAGAGAGAGAGAGAGAGAGAGAGAGAGAGAGAGA--TTG
p236_Coun_1   ACAC-GTAACCTCCTCCTTCCATTACTCCTCTAATTCTTCCCCCACACCTCACTGCACTTAAAATGCGCAGTTGCGCACTCACTGTGTGTGCTTATATGT--GAGAGAGAGAGAGAGAGA----------------------TTG
p236_Coun_2   ACAC-GTAACCTCCTCCTTCCATTACTCCTCTAATTCTTCCCCCACACCTCACTGCACTTAAAATGCGCAGTTGCGCACTCACTGTGTGTGCTTATATGT--GAGAGAGAGAGAGAGAGAGA--------------------TTG
p277_Coun_1   ACAC-GTAACCTCCTCCTTCCAT-ACTCCTCTAATTCTTCCCCCACACCTCACTGCACTTAAAATGCGCAGTTGCGCACTCACTGTGTGTGCTTATATGT--GAGAGAGAGAGA----------------------------TTG
p277_Coun_2   ACAC-GTAACCTCCTCCTTCCATTACTCCTCTAATTCT-CCCCCACACCTCACTGCACTTAAAATGCGCAGTTGCGCACTCACTGTGTGTGCTTATATGT--GAGAGAGAGAGAGAGAGAGAGAGAGAGA------------TTG
p305_Coun_1   ACACGGTAACCTCCTCCTTCCATTACTCCTCTAACTCTTCCCCCACACCTCACTGCACTTAAAATGCGCAGTTGCGCACTCGCTGTGTGTGCTTATATGT--GAGAGAGAGAGAGAGA------------------------TTG
p305_Coun_2   ACACGGTAACCTCCTCCTTCCATTACTCCTCTAACTCTTCCCCCACACCTCACTGCACTTAAAATGCGCAGTTGCGCACTCGCTGTGTGTGCTTATATGT--GAGAGAGAGAGAGAGAGAGAGAGA----------------TTG
p337_Coun_1   ACAC-GTAACCTCCTCCTTCCATTACTCCTCTAACTCTTCCCCCACACCTCACTGCACTTAAAATGCGCAGTTGCGCACTCGCTGTGTGTGCTTATATGT--GAGAGAGAGAGA----------------------------TTG
p337_Coun_2   ACAC-GTAACCTCCTCCTTCCATTACTCCTCTAACTCTTCCCCCACACCTCACTGCACTTAAAATGCGCAGTTGCGCACTCGCTGTGTGTGCTTATATGT--GAGAGAGAGAGAGAGAGAGAGAGAGAGAGAGAGAGAGA--TTG
p340_Coun_1   ACAC-GTAACCTCCTCCTTCCATTACTCCTCTAACTCTTCCCCCACACCTCACTGCACTTAAAATGCGCAGTTGCGCACTCGCTGTGTGTGCTTATATGT--GAGAGAGAGAGA----------------------------TTG
p340_Coun_2   ACAC-GTAACCTCCTCCTTCCATTACTCCTCTAACTCTTCCCCCACACCTCACTGCACTTAAAATGCGCAGTTGCGCACTCGCTGTGTGTGCTTATATGT--GAGAGAGAGAGAGAGAGAGAGAGAGAGAGAGAGA------TTG
p345_Coun_1   ACAC-GTAACCTCCTCCTTCCATTACTCCTCTAATTCT-CCCCCACACCTCACTGCACTTAAAATGCGCAGTTGCGCACTCGCTGTGTGTGCTTATATGT--GAGAGAGAGAGA----------------------------TTG
p345_Coun_2   ACAC-GTAACCTCCTCCTTCCATTACTCCTCTAATTCT-CCCCCACACCTCACTGCACTTAAAATGCGCAGTTGCGCACTCACTGTGTGTGCTTATATGT--GAGAGAGAGAGAGAGAGAGAGA------------------TTG
p346_Coun_1   ACAC-GTAACCTCCTCCTTCCATTACTCCTCTAACTCTTCCCCCACACCTCACTGCACTTAAAATGCGCAGTTGCGCACTCGCTGTGTGTGCTTATATGT--GAGAGAGAGAGAGAGAGA----------------------TTG
p346_Coun_2   ACAC-GTAACCTCCTCCTTCCATTACTCCTCTAACTCTTCCCCCACACCTCACTGCACTTAAAATGCGCAGTTGCGCACTCGCTGTGTGTGCTTATATGT--GAGAGAGAGAGA----------------------------TTG
p349_Coun_1   ACAC-GTAACCTCCTCCTTCCATTACTCCTCTAACTCTTCCCCCACACCTCACTGCACTTAAAATGCGCAGTTGCGCACTCGCTGTGTGTGCTTATATGT--GAGAGAGAGAGAGAGA------------------------TTG
p349_Coun_2   ACAC-GTAACCTCCTCCTTCCATTACTCCTCTAACTCTTCCCCCACACCTCACTGCACTTAAAATGCGCAGTTGCGCACTCGCTGTGTGTGCTTATATGT--GAGAGAGAGAGAGA--------------------------TTG
p390_Coun_1   ACAC-GTAACCTCCTCCTTCCATTACTCCTCTAACTCTTCCCCCACACCTCACTGCACTTAAAATGCGCAGTTGCGCACTCGCTGTGTGTGCTTATATGT--GAGAGAGAGAGAGAGAGAGAGAGAGAGAGAGA--------TTG
p390_Coun_2   ACAC-GTAACCTCCTCCTTCCATTACTCCTCTAACTCTTCCCCCACACCTCACTGCACTTAAAATGCGCAGTTGCGCACTCGCTGTGTGTGCTTATATGT--GAGAGAGAGAGAGAGAGAGAGAGAGAGA------------TTG
p470_Coun_1   ACAC-GTAACCTCCTCCTTCCAT-ACTCCTCTAATTCTTCCCCCACACCTCACTGCACTTAAAATGCGCAGTTGCGCACTCACTGTGTGTGCTTATATGT--GAGAGAGAGAGA----------------------------TTG
p470_Coun_2   ACAC-GTAACCTCCTCCTTCCATTACTCCTCTAACTCTTCCCCCACACCTCACTGCACTTAAAATGCGCAGTTGCGCACTCGCTGTGTGTGCTTATATGT--GAGAGAGAGAGAGAGAGAGA--------------------TTG
p495_Coun_1   ACAC-GTAACCTCCTCCTTCCATTACTCCTCTAACTCTTCCCCCACACCTCACTGCACTTAAAATGCGCAGTTGCGCACTCGCTGTGTGTGCTTATATGTGTGAGAGAGAGAGAGAGAGA----------------------TTG
p575_Coun_1   ACAC-GTAACCTCCTCCTTCCATTACTCCTCTAACTCTTCCCCCACACCTCACTGCACTTAAAATGCGCAGTTGCGCACTCGCTGTGTGTGCTTATATGT--GAGAGAGAGA------------------------------TTG
p575_Coun_2   ACAC-GTAACCTCCTCCTTCCATTACTCCTCTAACTCTTCCCCCACACCTCACTGCACTTAAAATGCGCAGTTGCGCACTCGCTGTGTGTGCTTATATGT--GAGAGAGAGAGA----------------------------TTG
F810_Para_1   ACAC-GTAACCTCCTCCTTCCATTACTCCTCTAACTCTTCCCCCACACCTCACTGCACTTAAAATGCGCAGTTGCGCACTCGCTGTGTGTGCTTATATGT--GAGAGAGAGAGAGAGAGA----------------------TTG
F810_Para_2   ACAC-GTAACCTCCTCCTTCCATTACTCCTCTAACTCTTCCCCCACACCTCACTGCACTTAAAATGCGCAGTTGCGCACTCGCTGTGTGTGCTTATATGT--GAGAGAGAGAGA----------------------------TTG
F885_Para_1   ACAC-GTAACCTCCTCCTTCCATTACTCCTCTAACTCTTCCCCCACACCTCACTGCACTTAAAATGCGCAGTTGCGCACTCGCTGTGTGTGCTTATATGT--GAGAGAGAGAGAGAGAGA----------------------TTG
F885_Para_2   ACAC-GTAACCTCCTCCTTCCATTACTCCTCTAACTCTTCCCCCAAACCTCACTGCACTTAAAATGCGCAGTTGCGCACTCGCTGTGTGTGCTTATATGT--GAGAGAGAGAGAGA--------------------------TT?
T308_Para_1   ACAC-GTAACCTCCTCCTTCCATTACTCCTCTAACTCTTCCCCCACACCTCACTGCACTTAAAATGCGCAGTTGCGCACTCGCTGTGTGTGCTTATATGT--GAGAGAGAGAGAGAGA------------------------TTG
T308_Para_2   ACAC-GTAACCTCCTCCTTCCATTACTCCTCTAACTCTTCCCCCACACCTCACTGCACTTAAAATGCGCAGTTGCGCACTCGCTGTGTGTGCTTATATGT--GAGAGAGAGAGA----------------------------TTG
T348_Para_1   ACAC-GTAACCTCCTCCTTCCATTACTCCTCTAACTCTTCCCCCACACCTCACTGCACTTAAAATGCGCAGTTGCGCACTCGCTGTGTGTGCTTATATGT--GAGAGAGAGAGAGAGAGA----------------------TTG
T348_Para_2   ACAC-GTAACCTCCTCCTTCCATTACTCCTCTAACTCTTCCCCCACACCTCACTGCACTTAAAATGCGCAGTTGCGCACTCGCTGTGTGTGCTTATATGT--GAGAGAGAGAGA----------------------------TTG
T350_Para_1   ACAC-GTAACCTCCTCCTTCCATTACTCCTCTAATTCT-CCCCCACACCTCACTGCACTTAAAATGCGCAGTTGCGCACTCGCTGTGTGTGCTTATATGT--GAGAGAGAGAGA----------------------------TTG
T350_Para_2   ACAC-GTAACCTCCTCCTTCCATTACTCCTCTAACTCTTCCCCCAAACCTCACTGCACTTAAAATGCGCAGTTGCGCACTCGCTGTGTGTGCTTATATGT--GAGAGAGAGAGA----------------------------TTG
T352_Para_1   ACAC-GTAACCTCCTCCTTCCATTACTCCTCTAACTCTTCCCCCACACCTCACTGCACTTAAAATGCGCAGTTGCGCACTCGCTGTGTGTGCTTATATGT--GAGAGAGAGAGAGAGAGAGAGAGAGAGAGA----------TTG
T352_Para_2   ACAC-GTAACCTCCTCCTTCCATTACTCCTCTAACTCTTCCCCCACACCTCACTGCACTTAAAATGCGCAGTTGCGCACTCGCTGTGTGTGCTTATATGT--GAGAGAGAGAGA----------------------------TTG
T354_Para_1   ACAC-GTAACCTCCTCCTTCCATTACTCCTCTAACTCTTCCCCCACACCTCACTGCACTTAAAATGCGCAGTTGCGCACTCGCTGTGTGTGCTTATATGT--GAGAGAGAGAGAGAGAGAGAGAGAGAGAGAGAGA------TTG
T354_Para_2   ACAC-GTAACCTCCTCCTTCCATTACTCCTCTAACTCTTCCCCCACACCTCACTGCACTTAAAATGCGCAGTTGCGCACTCGCTGTGTGTGCTTATATGT--GAGAGAGAGAGAGAGAGAGAGAGAGAGAGAGAGA------TTG
T358_Para_1   ACAC-GTAACCTCCTCCTTCCATTACTCCTCTAACTCTTCCCCCACACCTCACTGCACTTAAAATGCGCAGTTGCGCACTCGCTGTGTGTGCTTATATGT--GAGAGAGAGAGAGAGA------------------------TTG
T358_Para_2   ACAC-GTAACCTCCTCCTTCCATTACTCCTCTAACTCTTCCCCCACACCTCACTGCACTTAAAATGCGCAGTTGCGCACTCGCTGTGTGTGCTTATATGT--GAGAGAGAGAGA----------------------------TTG
T386_Para_1   ACAC-GTAACCTCCTCCTTCCATTACTCCTCTAATTCTTCCCCCACACCTCACTGCACTTAAAATGCGCAGTTGCGCACTCGCTGTGTGTGCTTATATGT--GAGAGAGAGAGAGAGAGAGAGAGAGA--------------TTG
T386_Para_2   ACAC-GTAACCTCCTCCTTCCATTACTCCTCTAACTCTTCCCCCACACCTCACTGCACTTAAAATGCGCAGTTGCGCACTCGCTGTGTGTGCTTATATGT--GAGAGAGAGAGAGAGAGAGA--------------------TTG
C731_Ouest_1  ACAC-GTAACCTCCTCCTTCCATTACTCCTCTAATTCTTCCCCCACACCTCACTGCACTTAAAATGCGCAGTTGCGCACTCACTGTGTGTGCTTATATGT--GAGAGAGAGAGAGAGAGAGA--------------------TTG
C731_Ouest_2  ACAC-GTAACCTCCTCCTTCCATTACTCCTCTAATTCTTCCCCCACACCTCACTGCACTTAAAATGCGCAGTTGCGCACTCACTGTGTGTGCTTATATGT--GAGAGAGAGAGAGAGAGAGA--------------------TTG
C733_Ouest_1  ACAC-GTAACCTCCTCCTTCCATTACTCCTCTAACTCTTCCCCCACACCTCACTGCACTTAAAATGCGCAGTTGCGCACTCGCTGTGTGTGCTTATATGT--GAGAGAGAGAGAGAGAGAGAGA------------------TTG
C733_Ouest_2  ACAC-GTAACCTCCTCCTTCCATTACTCCTCTAATTCTTCCCCCACACCTCACTGCACTTAAAATGCGCAGTTGCGCACTCACTGTGTGTGCTTATATGT--GAGAGAGAGAGA----------------------------TTG
C735_Ouest_1  ACAC-GTAACCTCCTCCTTCCATTACTCCTCTAACTCTTCCCCCACACCTCACTGCACTTAAAATGCGCAGTTGCGCACTCGCTGTGTGTGCTTATATGT--GAGAGAGAGAGAGAGAGAGA--------------------TTG
C735_Ouest_2  ACAC-GTAACCTCCTCCTTCCATTACTCCTCTAACTCTTCCCCCACACCTCACTGCACTTAAAATGCGCAGTTGCGCACTCGCTGTGTGTGCTTATATGT--GAGAGAGAGAGAGAGAGA----------------------TTG
C737_Ouest_1  ACAC-GTAACCTCCTCCTTCCATTACTCCTCTAACTCTTCCCCCACACCTCACTGCACTTAAAATGCGCAGTTGCGCACTCGCTGTGTGTGCTTATATGT--GAGAGAGAGAGAGAGAGAGAGAGAGAGAGAGAGAGAGA--TTG
C737_Ouest_2  ACAC-GTAACCTCCTCCTTCCATTACTCCTCTAACTCTTCCCCCACACCTCACTGCACTTAAAATGCGCAGTTGCGCACTCGCTGTGTGTGCTTATATGT--GAGAGAGAGAGAGAGA------------------------TTG
```

```
C758_Ouest_1   ACAC-GTAACCTCCTCCTTCCATTACTCCTCTAACTCTTCCCCCACACCTCACTGCACTTAAAATGCGCAGTTGCGCACTCGCTGTGTGTGCTTATATGT--GAGAGAGAGAGA----------------------------TTG
C758_Ouest_2   ACAC-GTAACCTCCTCCTTCCATTACTCCTCTAACTCTTCCCCCACACCTCACTGCACTTAAAATGCGCAGTTGCGCACTCGCTGTGTGTGCTTATATGT--GAGAGAGAGAGA----------------------------TTG
C784_Ouest_1   ACAC-GTAACCTCCTCCTTCCATTACTCCTCTAACTCTTCCCCCACACCTCACTGCACTTAAAATGCGCAGTTGCGCACTCGCTGTGTGTGCTTATATGT--GAGAGAGAGAGAGAGAGA----------------------TTG
C784_Ouest_2   ACAC-GTAACCTCCTCCTTCCATTACTCCTCTAACTCTTCCCCCACACCTCACTGCACTTAAAATGCGCAGTTGCGCACTCGCTGTGTGTGCTTATATGT--GAGAGAGAGAGA----------------------------TTG
C793_Ouest_1   ACAC-GTAACCTCCTCCTTCCATTACTCCTCTAACTCTTCCCCCACACCTCACTGCACTTAAAATGCGCAGTTGCGCACTCGCTGTGTGTGCTTATATGT--GAGAGAGAGAGAGAGAGA----------------------TTG
C793_Ouest_2   ACAC-GTAACCTCCTCCTTCCATTACTCCTCTAACTCTTCCCCCACACCTCACTGCACTTAAAATGCGCAGTTGCGCACTCGCTGTGTGTGCTTATATGT--GAGAGAGAGAGA----------------------------TTG
C913_Ouest_1   ACAC-GTAACCTCCTCCTTCCATTACTCCTCTAATTCT-CCCCCACACCTCACTGCACTTAAAATGCGCAGTTGCGCACTCACTGTGTGTGCTTATATGT--GAGAGAGAGAGAGAGAGAGAGA------------------TTG
C913_Ouest_2   ACAC-GTAACCTCCTCCTTCCATTACTCCTCTAATTCT-CCCCCACACCTCACTGCACTTAAAATGCGCAGTTGCGCACTCACTGTGTGTGCTTATATGT--GAGAGAGAGAGAGAGA------------------------TTG
C916_Ouest_1   ACAC-GTAACCTCCTCCTTCCATTACTCCTCTAATTCT-CCCCCACACCTCACTGCACTTAAAATGCGCAGTTGCGCACTCACTGTGTGTGCTTATATGT--GAGAGAGAGAGAGAGAGAGAGA------------------TTG
C916_Ouest_2   ACAC-GTAACCTCCTCCTTCCATTACTCCTCTAATTCT-CCCCCACACCTCACTGCACTTAAAATGCGCAGTTGCGCACTCACTGTGTGTGCTTATATGT--GAGAGAGAGAGAGAGAGAGA--------------------TTG
C921_Ouest_1   ACAC-GTAACCTCCTCCTTCCATTACTCCTCTAATTCT-CCCCCACACCTCACTGCACTTAAAATGCGCAGTTGCGCACTCACTGTGTGTGCTTATATGT--GAGAGAGAGAGAGAGAGAGAGA------------------TTG
S252_Ouest_1   ACAC-GTAACCTCCTCCTTCCATTACTCCTCTAATTCT-CCCCCACACCTCACTGCACTTAAAATGCGCAGTTGCGCACTCACTGTGTGTGCTTATATGT--GAGAGAGAGAGAGAGA------------------------TTG
S252_Ouest_2   ACAC-GTAACCTCCTCCTTCCATTACTCCTCTAATTCT-CCCCCACACCTCACTGCACTTAAAATGCGCAGTTGCGCACTCACTGTGTGTGCTTATATGT--GAGAGAGAGAGAGAGA------------------------TTG
S256_Ouest_1   ACAC-GTAACCTCCTCCTTCCATTACTCCTCTAATTCT-CCCCCACACCTCACTGCACTTAAAATGCGCAGTTGCGCACTCACTGTGTGTGCTTATATGT--GAGAGAGAGAGAGAGA------------------------TTG
S256_Ouest_2   ACAC-GTAACCTCCTCCTTCCATTACTCCTCTAATTCT-CCCCCACACCTCACTGCACTTAAAATGCGCAGTTGCGCACTCACTGTGTGTGCTTATATGT--GAGAGAGAGAGAGAGA------------------------TTG
S258_Ouest_1   ACAC-GTAACCTCCTCCTTCCATTACTCCTCTAATTCT-CCCCCACACCTCACTGCACTTAAAATGCGCAGTTGCGCACTCACTGTGTGTGCTTATATGT--GAGAGAGAGAGAGAGAGAGA--------------------TTG
S258_Ouest_2   ACAC-GTAACCTCCTCCTTCCATTACTCCTCTAATTCT-CCCCCACACCTCACTGCACTTAAAATGCGCAGTTGCGCACTCACTGTGTGTGCTTATATGT--GAGAGAGAGAGAGAGA------------------------TTG
S260_Ouest_1   ACAC-GTAACCTCCTCCTTCCATTACTCCTCTAATTCT-CCCCCACACCTCACTGCACTTAAAATGCGCAGTTGCGCACTCACTGTGTGTGCTTATATGT--GAGAGAGAGAGAGAGA------------------------TTG
S260_Ouest_2   ACAC-GTAACCTCCTCCTTCCATTACTCCTCTAATTCT-CCCCCACACCTCACTGCACTTAAAATGCGCAGTTGCGCACTCACTGTGTGTGCTTATATGT--GAGAGAGAGAGAGAGA------------------------TTG
S262_Ouest_1   ACAC-GTAACCTCCTCCTTCCATTACTCCTCTAATTCT-CCCCCACACCTCACTGCACTTAAAATGCGCAGTTGCGCACTCACTGTGTGTGCTTATATGT--GAGAGAGAGAGAGAGAGAGA--------------------TTG
S262_Ouest_2   ACAC-GTAACCTCCTCCTTCCATTACTCCTCTAATTCT-CCCCCACACCTCACTGCACTTAAAATGCGCAGTTGCGCACTCACTGTGTGTGCTTATATGT--GAGAGAGAGAGAGAGA------------------------TTG
S263_Ouest_1   ACAC-GTAACCTCCTCCTTCCATTACTCCTCTAATTCT-CCCCCACACCTCACTGCACTTAAAATGCGCAGTTGCGCACTCACTGTGTGTGCTTATATGT--GAGAGAGAGAGAGAGA------------------------TTG
S263_Ouest_2   ACAC-GTAACCTCCTCCTTCCATTACTCCTCTAATTCT-CCCCCACACCTCACTGCACTTAAAATGCGCAGTTGCGCACTCACTGTGTGTGCTTATATGT--GAGAGAGAGAGAGAGA------------------------TTG
S264_Ouest_1   ACAC-GTAACCTCCTCCTTCCATTACTCCTCTAATTCT-CCCCCACACCTCACTGCACTTAAAATGCGCAGTTGCGCACTCACTGTGTGTGCTTATATGT--GAGAGAGAGAGAGAGAGAGA--------------------TTG
S264_Ouest_2   ACAC-GTAACCTCCTCCTTCCATTACTCCTCTAATTCT-CCCCCACACCTCACTGCACTTAAAATGCGCAGTTGCGCACTCACTGTGTGTGCTTATATGT--GAGAGAGAGAGAGAGA------------------------TTG
S265_Ouest_1   ACAC-GTAACCTCCTCCTTCCATTACTCCTCTAATTCT-CCCCCACACCTCACTGCACTTAAAATGCGCAGTTGCGCACTCACTGTGTGTGCTTATATGT--GAGAGAGAGAGAGAGA------------------------TTG
S265_Ouest_2   ACAC-GTAACCTCCTCCTTCCATTACTCCTCTAATTCT-CCCCCACACCTCACTGCACTTAAAATGCGCAGTTGCGCACTCACTGTGTGTGCTTATATGT--GAGAGAGAGAGAGAGA------------------------TTG
S266_Ouest_1   ACAC-GTAACCTCCTCCTTCCATTACTCCTCTAATTCT-CCCCCACACCTCACTGCACTTAAAATGCGCAGTTGCGCACTCACTGTGTGTGCTTATATGT--GAGAGAGAGAGAGAGAGAGAGAGA----------------TTG
S266_Ouest_2   ACAC-GTAACCTCCTCCTTCCATTACTCCTCTAATTCT-CCCCCACACCTCACTGCACTTAAAATGCGCAGTTGCGCACTCACTGTGTGTGCTTATATGT--GAGAGAGAGAGAGAGAGAGA--------------------TTG
S268_Ouest_1   ACAC-GTAACCTCCTCCTTCCATTACTCCTCTAATTCT-CCCCCACACCTCACTGCACTTAAAATGCGCAGTTGCGCACTCACTGTGTGTGCTTATATGT--GAGAGAGAGAGAGAGA------------------------TTG
S268_Ouest_2   ACAC-GTAACCTCCTCCTTCCATTACTCCTCTAATTCT-CCCCCACACCTCACTGCACTTAAAATGCGCAGTTGCGCACTCACTGTGTGTGCTTATATGT--GAGAGAGAGAGAGAGA------------------------TTG
S270_Ouest_1   ACAC-GTAACCTCCTCCTTCCATTACTCCTCTAATTCT-CCCCCACACCTCACTGCACTTAAAATGCGCAGTTGCGCACTCACTGTGTGTGCTTATATGT--GAGAGAGAGAGAGAGAGAGAGAGA----------------TTG
S270_Ouest_2   ACAC-GTAACCTCCTCCTTCCATTACTCCTCTAATTCT-CCCCCACACCTCACTGCACTTAAAATGCGCAGTTGCGCACTCACTGTGTGTGCTTATATGT--GAGAGAGAGAGAGAGA------------------------TTG
S274_Ouest_1   ACAC-GTAACCTCCTCCTTCCATTACTCCTCTAACTCTTCCCCCACACCTCACTGCACTTAAAATGCGCAGTTGCGCACTCGCTGTGTGTGCTTATATGT--GAGAGAGAGAGAGAGAGAGA--------------------TTG
S274_Ouest_2   ACAC-GTAACCTCCTCCTTCCATTACTCCTCTAATTCTTCCCCCACACCTCACTGCACTTAAAATGCGCAGTTGCGCACTCACTGTGTGTGCTTATATGT--GAGAGAGAGAGAGAGAGAGAGAGAGAGA------------TTG
S295_Ouest_1   ACAC-GTAACCTCCTCCTTCCATTACTCCTCTAACTCTTCCCCCACACCTCACTGCACTTAAAATGCGCAGTTGCGCACTCACTGTGTGTGCTTATATGT--GAGAGAGAGAGAGAGAGA----------------------TTG
OUT192_Bre_1   ACAC-GTAACCTCCTCCTTCCATTACTCCTCTAATTCT-CCCCCACACCTCACTGCACTTAAAATGCGCAGTTGCGCACTCACTGTGTGTGCTTATATGT--GAGAGAGAGAGAGAGA------------------------TTG
OUT192_Bre_2   ACAC-GTAACCTCCTCCTTCCATTACTCCTCTAATTCT-CCCCCACACCTCACTGCACTTAAAATGCGCAGTTGCGCACTCACTGTGTGTGCTTATATGT--GAGAGAGAGAGAGAGAGAGAGA------------------TTG
OUT194_Bre_1   ACAC-GTAACCTCCTCCTTCCATTACTCCTCTAATTCT-CCCCCACACCTCACTGCACTTAAAATGCGCAGTTGCGCACTCACTGTGTGTGCTTATATGT--GAGAGAGAGAGAGAGA------------------------TTG
OUT194_Bre_2   ACAC-GTAACCTCCTCCTTCCATTACCCCTCTAATTCT-CCCCCACACCTCACTGCACTTAAAATGCGCAGTTGCGCACTCACTGTGTGTGCTTATATGT--GAGAGAGAGAGAGAGAGAGAGA------------------TTG
OUT198_Bre_1   ACAC-GTAACCTCCTCCTTCCATTACTCCTCTAATTCT-CCCCCACACCTCACTGCACTTAAAATGCGCAGTTGCGCACTCACTGTGTGTGCTTATATGT--GAGAGAGAGAGAGAGA------------------------TTG
OUT198_Bre_2   ACAC-GTAACCTCCTCCTTCCATTACTCCTCTAATTCT-CCCCCACACCTCACTGCACTTAAAATGCGCAGTTGCGCACTCACTGTGTGTGCTTATATGT--GAGAGAGAGAGAGAGAGAGA--------------------TTG
OUT204_Bre_1   ACAC-GTAACCTCCTCCTTCCATTACTCCTCTAATTCT-CCCCCACACCTCACTGCACTTAAAATGCGCAGTTGCGCACTCACTGTGTGTGCTTATATGT--GAGAGAGAGAGAGAGA------------------------TTG
OUT204_Bre_2   ACAC-GTAACCTCCTCCTTCCATTACTCCTCTAATTCT-CCCCCACACCTCACTGCACTTAAAATGCGCAGTTGCGCACTCACTGTGTGTGCTTATATGT--GAGAGAGAGAGAGAGAGAGAGAGA----------------TTG
OUT213_Bre_1   ACAC-GTAACCTCCTCCTTCCATTACTCCTCTAATTCT-CCCCCACACCTCACTGCACTTAAAATGCGCAGTTGCGCACTCACTGTGTGTGCTTATATGT--GAGAGAGAGAGAGAGAGAGAGA------------------TTG
OUT213_Bre_2   ACAC-GTAACCTCCTCCTTCCATTACTCCTCTAATTCT-CCCCCACACCTCACTGCACTTAAAATGCGCAGTTGCGCACTCACTGTGTGTGCTTATATGT--GAGAGAGAGAGAGAGAGAGAGA------------------TTG
```

Jc3H10

```
p186_Coun_1    TTAGACTCTTAGTGCAGCATGACTCTCTTCCTGTCT---------------------------------------------CTCTCTCTCTCTCTCTCTCTCTCTCTCTCTCTCTC------ATACACACACACACGCACGCACG
p186_Coun_2    TTAGACTCTTAGTGCAGCATGACTCTCTTCCTGTCT-----------------------------------------CTCTCTCTCTCTCTCTCTCTCTCTCTCTCTCTCTCTCTC------ATACACACACACACGCACGCACG
p216_Coun_1    TTAGACTCTTAGTGCAGCATGACTCTCTTCCTGTCT---------------------------------------------------------CTCTCTCTCTCTCTCTCTCTCTC------ATACACACACACACGCACGCACG
p216_Coun_2    TTAGACTCTTAGTGCAGCATGACTCTCTTCCTGTCT---------------------------------------------------------CTCTCTCTCTCTCTCTCTCTCTC------ATACACACACACACGCACGCACG
p236_Coun_1    TTAGACTCTTAGTGCAGCATGACTCTCTTCCTGTCT-----------------------------------------------------------------CTCTCTCTCTCTCTC------ATACACACACACACGCACGCACG
p236_Coun_2    TTAGACTCTTAGTGCAGCATGACTCTCTTCCTGTCT---------------------------------------------------CTCTCTCTCTCTCTCTCTCTCTCTCTCTC------ATACACACACACACGCACGCACG
p277_Coun_1    TTAGACTCTTAGTGCAGCATGACTCTCTTCCTGTCT-------------------------------------------------------------CTCTCTCTCTCTCTCTCTC------ATACACACACACACGCACG----
```

```
p277_Coun_2     TTAGACTCTTAGTGCAGCATGACTCTCTTCCTGTCT-----------------------------------------------------------CTCTCTCTCTCTCTCTCTCTC------ATACACACACACACGCACGCACG
p305_Coun_1     TTAGACTCTTAGTGCAGCATGACTCTCTTCCTGTCT-----------------------------------------------------------CTCTCTCTCTCTCTCTCTCTC------ATACACACACACACGCACG----
p305_Coun_2     TTAGACTCTTAGTGCAGCATGACTCTCTTCCTGTCT---------------------------------------------------CTCTCTCTCTCTCTCTCTCTCTCTCTCTC------ATACACACACACACGCACGCACG
p307_Coun_1     TTAGACTCTTAGTGCAGCATGACTCTCTTCCTGTCT---------------------------------CTCTCTCTCTCTCTCTCTCTCTCTCTCTCTCTCTCTCTCTCTCTCTC------ATACACACACACACGCACGCACG
p307_Coun_2     TTAGACTCTTAGTGCAGCATGACTCTCTTCCTGTCT-------------------------------CTCTCTCTCTCTCTCTCTCTCTCTCTCTCTCTCTCTCTCTCTCTCTCTC------ATACACACACACACGCACGCACG
p337_Coun_1     TTAGACTCTTAGTGCAGCATGACTCTCTTCCTGTCT-------------------------------------------CTCTCTCTCTCTCTCTCTCTCTCTCTCTCTCTCTCTC------ATACACACACACACGCACG----
p337_Coun_2     TTAGACTCTTAGTGCAGCATGACTCTCTTCCTGTCT-----------------------------------------------CTCTCTCTCTCTCTCTCTCTCTCTCTCTCTCTC------ATACACACACACACGCACGCACG
p339_Coun_1     TTAGACTCTTAGTGCAGCATGACTCTCTTCCTGTCT-----------------------------------------------------------CTCTCTCTCTCTCTCTCTCTC------ATACACACACACACGCACG----
p339_Coun_2     TTAGACTCTTAGTGCAGCATGACTCTCTTCCTGTCT-----------------------------------------------------------CTCTCTCTCTCTCTCTCTCTC------ATACACACACACACGCACG----
p345_Coun_1     TTAGACTCTTAGTGCAGCATGACTCTCTTCCTGTCT-----------------------------------------------------------CTCTCTCTCTCTCTCTCTCTC------ATACACACACACACGCACGCACG
p345_Coun_2     TTAGACTCTTAGTGCAGCATGACTCTCTTCCTGTCT-----------------------------------------------------------CTCTCTCTCTCTCTCTCTCTC------ATACACACACACACGCACG----
p349_Coun_1     TTAGACTCTTAGTGCAGCATGACTCTCTTCCTGTCT-----------------------------------------------------------CTCTCTCTCTCTCTCTCTCTC------ATACACACACACACGCACG----
p349_Coun_2     TTAGACTCTTAGTGCAGCATGACTCTCTTCCTGTCT-----------------------------------------------------------CTCTCTCTCTCTCTCTCTCTC------ATACACACACACACGCACG----
p374_Coun_1     TTAGACTCTTAGTGCAGCATGACTCTCTTCCTGTCT-----------------------------------------------------------CTCTCTCTCTCTCTCTCTCTC------ATACACACACACACGCACG----
p374_Coun_2     TTAGACTCTTAGTGCAGCATGACTCTCTTCCTGTCT-----------------------------------------------------------CTCTCTCTCTCTCTCTCTCTC------ATACACACACACACGCACGCACG
p390_Coun_1     TTAGACTCTTAGTGCAGCATGACTCTCTTCCTGTCT---------------------------------------------------------CTCTCTCTCTCTCTCTCTCTCTC------ATACACACACACACGCACG----
p390_Coun_2     TTAGACTCTTAGTGCAGCATGACTCTCTTCCTGTCT-----------------------------------------------CTCTCTCTCTCTCTCTCTCTCTCTCTCTCTCTC------ATACACACACACACGCACGCACG
p441_Coun_1     TTAGACTCTTAGTGCAGCATGACTCTCTTCCTGTCT-----------------------------------------CTCTCTCTCTCTCTCTCTCTCTCTCTCTCTCTCTCTCTC------ATACACACACACACGCACGCACG
p441_Coun_2     TTAGACTCTTAGTGCAGCATGACTCTCTTCCTGTCT---------------------------------------CTCTCTCTCTCTCTCTCTCTCTCTCTCTCTCTCTCTCTCTC------ATACACACACACACGCACGCACG
p470_Coun_1     TTAGACTCTTAGTGCAGCATGACTCTCTTCCTGTCT-----------------------------------------CTCTCTCTCTCTCTCTCTCTCTCTCTCTCTCTCTCTCTC------ATACACACACACACGCACG----
p470_Coun_2     TTAGACTCTTAGTGCAGCATGACTCTCTTCCTGTCT-----------------------------------------CTCTCTCTCTCTCTCTCTCTCTCTCTCTCTCTCTCTCTC------ATACACACACACACGCACGCACG
p495_Coun_1     TTAGACTCTTAGTGCAGCATGACTCTCTTCCTGTCT-----------------------------------------------------------CTCTCTCTCTCTCTCTCTCTC------ATACACACACACACGCACG----
p495_Coun_2     TTAGACTCTTAGTGCAGCATGACTCTCTTCCTGTCT-----------------------------------------------------------CTCTCTCTCTCTCTCTCTCTC------ATACACACACACACGCACG----
p575_Coun_1     TTAGACTCTTAGTGCAGCATGACTCTCTTCCTGTCT-----------------------------------------CTCTCTCTCTCTCTCTCTCTCTCTCTCTCTCTCTCTCTC------ATACACACACACACGCACGCACG
p575_Coun_2     TTAGACTCTTAGTGCAGCATGACTCTCTTCCTGTCT---------------------------------------------CTCTCTCTCTCTCTCTCTCTCTCTCTCTCTCTCTC------ATACACACACACACGCACG----
F1038_Para_1    TTAGACTCTTAGTGCAGCATGACTCTCTTCCTGTCT---------------------------------------------CTCTCTCTCTCTCTCTCTCTCTCTCTCTCTCTCTC------AT------------GCACG----
F1038_Para_2    TTAGACTCTTAGTGCAGCATGACTCTCTTCCTGTCT-----------------------------------------------------------CTCTCTCTCTCTCTCTCTCTC------ATACACACACACACGCACG----
F810_Para_1     TTAGACTCTTAGTGCAGCATGACTCTCTTCCTGTCT-----------------------------------------------------------CTCTCTCTCTCTCTCTCTCTC------ATACACACACACACGCACG----
F810_Para_2     TTAGACTCTTAGTGCAGCATGACTCTCTTCCTGTCT---------------------------------------------------------CTCTCTCTCTCTCTCTCTCTCTC------ATACACACACACACGCACGCACG
T308_Para_1     TTAGACTCTTAGTGCAGCATGACTCTCTTCCTGTCT-----------------------------------------------------------CTCTCTCTCTCTCTCTCTCTC------ATACACACACACACGCACG----
T308_Para_2     TTAGACTCTTAGTGCAGCATGACTCTCTTCCTGTCT-----------------------------------------------------------CTCTCTCTCTCTCTCTCTCTC------ATACACACACACACGCACG----
T352_Para_1     TTAGACTCTTAGTGCAGCATGACTCTCTTCCTGTCT-----------------------------------------------------------CTCTCTCTCTCTCTCTCTCTC------ATACACACACACACGCACG----
T352_Para_2     TTAGACTCTTAGTGCAGCATGACTCTCTTCCTGTCT---------------------------------------CTCTCTCTCTCTCTCTCTCTCTCTCTCTCTCTCTCTCTCTC------ATACACACACACACGCACG----
T354_Para_1     TTAGACTCTTAGTGCAGCATGACTCTCTTCCTGTCT-----------------------------------------------------------CTCTCTCTCTCTCTCTCTCTC------ATACACACACACACGCACGCACG
T354_Para_2     TTAGACTCTTAGTGCAGCATGACTCTCTTCCTGTCT---------------------------------------------------------CTCTCTCTCTCTCTCTCTCTCTC------ATACACACACACACGCACG----
T360_Para_1     TTAGACTCTTAGTGCAGCATGACTCTCTTCCTGTCT-----------------------------CTCTCTCTCTCTCTCTCTCTCTCTCTCTCTCTCTCTCTCTCTCTCTCTCTC------ATACACACACACACGCACG----
T360_Para_2     TTAGACTCTTAGTGCAGCATGACTCTCTTCCTGTCT-------------------------------CTCTCTCTCTCTCTCTCTCTCTCTCTCTCTCTCTCTCTCTCTCTCTCTC------ATACACACACACACGCACG----
T372_Para_1     TTAGACTCTTAGTGCAGCATGACTCTCTTCCTGTCT-------------------------CTCTCTCTCTCTCTCTCTCTCTCTCTCTCTCTCTCTCTCTCTCTCTCTCTCTCTCTC------ATACACACACACACGCACGCACG
T372_Para_2     TTAGACTCTTAGTGCAGCATGACTCTCTTCCTGTCT-----------------------------------CTCTCTCTCTCTCTCTCTCTCTCTCTCTCTCTCTCTCTCTCTCTC------ATACACACACACACGCACG----
T386_Para_1     TTAGACTCTTAGTGCAGCATGACTCTCTTCCTGTCT-----------------------------------------------------------CTCTCTCTCTCTCTCTCTCTC------ATACACACACACACGCACG----
T386_Para_2     TTAGACTCTTAGTGCAGCATGACTCTCTTCCTGTCT-----------------------------------------------------------CTCTCTCTCTCTCTCTCTCTC------ATACACACACACACGCACG----
T430_Para_1     TTAGACTCTTAGTGCAGCATGACTCTCTTCCTGTCT-----------------------------------------------------------CTCTCTCTCTCTCTCTCTCTC------ATACACACACACACGCACG----
T430_Para_2     TTAGACTCTTAGTGCAGCATGACTCTCTTCCTGTCT-----------------------------------------------------------CTCTCTCTCTCTCTCTCTCTC------ATACACACACACACGCACG----
T432_Para_1     TTAGACTCTTAGTGCAGCATGACTCTCTTCCTGTCT-----------------------------------------------------------CTCTCTCTCTCTCTCTCTCTC------ATACACACACACACGCACG----
T432_Para_2     TTAGACTCTTAGTGCAGCATGACTCTCTTCCTGTCT---------------------------------------------------CTCTCTCTCTCTCTCTCTCCCTCTCTCTC------ATACACACACACACGCACGCACG
C733_Ouest_1    TTAGACTCTTAGTGCAGCATGACTCTCTTCCTGTCT---------------------------------CTCTCTCTCTCTCTCTCTCTCTCTCTCTCTCTCTCTCTCTCTCTCTC------ATACACACACACACGCACGCACG
C733_Ouest_2    TTAGACTCTTAGTGCAGCATGACTCTCTTCCTGTCT-----------------------CTCTCTCTCTCTCTCTCTCTCTCTCTCTCTCTCTCTCTCTCTCTCTCTCTCTCTCTCTC------ATACACACACACACGCACGCACG
C735_Ouest_1    TTAGACTCTTAGTGCAGCATGACTCTCTTCCTGTCT-------------------------------------------------------CTCTCTCTCTCTCTCTCTCTCTCTC------ATACACACACACACGCACGCACG
C735_Ouest_2    TTAGACTCTTAGTGCAGCATGACTCTCTTCCTGTCT-------------------------------------------CTCTCTCTCTCTCTCTCTCTCTCTCTCTCTCTCTCTC------ATACACACACACACGCACGCACG
C737_Ouest_1    TTAGACTCTTAGTGCAGCATGACTCTCTTCCTGTCT-------------------------------------------CTCTCTCTCTCTCTCTCTCTCTCTCTCTCTCTCTCTC------ATACACACACACACGCACGCACG
C737_Ouest_2    TTAGACTCTTAGTGCAGCATGACTCTCTTCCTGTCT-----------------------------CTCTCTCTCTCTCTCTCTCTCTCTCTCTCTCTCTCTCTCTCCCTCTCTCTC------ATACACACACACACGCACGCACG
C758_Ouest_1    TTAGACTCTTAGTGCAGCATGACTCTCTTCCTGTCT---------------------------------------------------------CTCTCTCTCTCTCTCTCTCTCTC------ATACACACACACACGCACGCACG
C758_Ouest_2    TTAGACTCTTAGTGCAGCATGACTCTCTTCCTGTCT---------------------------------------------CTCTCTCTCTCTCTCTCTCTCTCTCTCTCTCTCTC------ATACACACACACACGCACG----
C793_Ouest_1    TTAGACTCTTAGTGCAGCATGACTCTCTTCCTGTCT---------------------------------------------CTCTCTCTCTCTCTCTCTCTCTCTCTCTCTCTCTC------ATACACACACACACGCACGCACG
C793_Ouest_2    TTAGACTCTTAGTGCAGCATGACTCTCTTCCTGTCT-----------------------------------------------------------CTCTCTCTCTCCCTCTCTCTC------ATACACACACACACGCACG----
C913_Ouest_1    TTAGACTCTTAGTGCAGCGTGACTCTCTTCCTGTCTCACACATACCCTCTG--------------------------------------------CTCTCTCTCTCTCTCTCTCT------------------CACGCACGCACG
C913_Ouest_2    TTAGACTCTTAGTGCAGCATGACTCTCTTCCTGTCT---------------------------------------------------CTCTCTCTCTCTCTCTCTCTCTCTCTCTC------ATACACACACACACGCACGCACG
```

```
C916_Ouest_1   TTAGACTCTTAGTGCAGCGTGACTCTCTTCCTGTCTCACACATACCCTCTG--------------------------------------------CTCTCTCTCTCTCTCTCTCT------------------CACGCACGCACG
C916_Ouest_2   TTAGACTCTTAGTGCAGCGTGACTCTCTTCCTGTCTCACACATACCCTCTG------------------------------------------CTCTCTCTCTCTCTCTCTCTCT------------------CACGCACGCACG
C921_Ouest_1   TTAGACTCTTAGTGCAGCATGACTCTCTTCCTGTCT-----------------------------------------------------------CTCTCTCTCTCTCTCTCTCTC------ATACACACACACACGCACG----
C921_Ouest_2   TTAGACTCTTAGTGCAGCATGACTCTCTTCCTGTCT-----------------------------------------------------------CTCTCTCTCTCTCTCTCTCTC------ATACACACACACACGCACG----
S258_Ouest_1   TTAGACTCTTAGTGCAGCGTGACTCTCTTCCTGTCTCACACATACCCTCTG--------------------------------------------CTCTCTCTCTCTCTCTCTCT------------------CACGCACGCACG
S258_Ouest_2   TTAGACTCTTAGTGCAGCGTGACTCTCTTCCTGTCTCACACATACCCTCTG------------------------------------------CTCTCTCTCTCTCTCTCTCTCT------------------CACGCACGCACG
S260_Ouest_1   TTAGACTCTTAGTGCAGCGTGACTCTCTTCCTGTCTCACACATACCCTCTG--------------------------------------------CTCTCTCTCTCTCTCTCTCT------------------CACGCACGCACG
S260_Ouest_2   TTAGACTCTTAGTGCAGCGTGACTCTCTTCCTGTCTCACACATACCCTCTG------------------------------------------CTCTCTCTCTCTCTCTCTCTCT------------------CACGCACGCACG
S262_Ouest_1   TTAGACTCTTAGTGCAGCGTGACTCTCTTCCTGTCTCACACATACCCTCTG--------------------------------------------CTCTCTCTCTCTCTCTCTCT------------------CACGCACGCACG
S262_Ouest_2   TTAGACTCTTAGTGCAGCGTGACTCTCTTCCTGTCTCACACATACCCTCTG--------------------------------------------CTCTCTCTCTCTCTCTCTCT------------------CACGCACGCACG
S263_Ouest_1   TTAGACTCTTAGTGCAGCGTGACTCTCTTCCTGTCTCACACATACCCTCTG--------------------------------------------CTCTCTCTCTCTCTCTCTCT------------------CACGCACGCACG
S263_Ouest_2   TTAGACTCTTAGTGCAGCGTGACTCTCTTCCTGTCTCACACATACCCTCTG------------------------------------------CTCTCTCTCTCTCTCTCTCTCT------------------CACGCACGCACG
S264_Ouest_1   TTAGACTCTTAGTGCAGCGTGACTCTCTTCCTGTCTCACACATACCCTCTG--------------------------------------------CTCTCTCTCTCTCTCTCTCT------------------CACGCACGCACG
S264_Ouest_2   TTAGACTCTTAGTGCAGCGTGACTCTCTTCCTGTCTCACACATACCCTCTG--------------------------------------------CTCTCTCTCTCTCTCTCTCT------------------CACGCACGCACG
S265_Ouest_1   TTAGACTCTTAGTGCAGCGTGACTCTCTTCCTGTCTCACACATACCCTCTG------------------------------------------CTCTCTCTCTCTCTCTCTCTCT------------------CACGCACGCACG
S265_Ouest_2   TTAGACTCTTAGTGCAGCGTGACTCTCTTCCTGTCTCACACATACCCTCTG------------------------------------------CTCTCTCTCTCTCTCTCTCTCT------------------CACGCACGCACG
S266_Ouest_1   TTAGACTCTTAGTGCAGCGTGACTCTCTTCCTGTCTCACACATACCCTCTG--------------------------------------------CTCTCTCTCTCTCTCTCTCT------------------CACGCACGCACG
S266_Ouest_2   TTAGACTCTTAGTGCAGCGTGACTCTCTTCCTGTCTCACACATACCCTCTG--------------------------------------------CTCTCTCTCTCTCTCTCTCT------------------CACGCACGCACG
S274_Ouest_1   TTAGACTCTTAGTGCAGCATGACTCTCTTCCTGTCT---------------------------------CTCTCTCTCTCTCTCTCTCTCTCTCTCTCTCTCTCTCTCTCTCTCTC------ATACACACACACACGCACG----
S274_Ouest_2   TTAGACTCTTAGTGCAGCATGACTCTCTTCCTGTCT-------------------------------CTCTCTCTCTCTCTCTCTCTCTCTCTCTCTCTCTCTCTCTCTCTCTCTC------ATACACACACACACGCACGCACG
S295_Ouest_1   TTAGACTCTTAGTGCAGCATGACTCTCTTCCTGTCT---------------------CTCTCTCTCTCTCTCTCTCTCTCTCTCTCTCTCTCTCTCTCTCTCTCTCCCTCTCTCTC------ATACACACACACACGCACGCACG
S295_Ouest_2   TTAGACTCTTAGTGCAGCATGACTCTCTTCCTGTCT-----------------------CTCTCTCTCTCTCTCTCTCTCTCTCTCTCTCTCTCTCTCTCTCTCTCCCTCTCTCTC------ATACACACACACACGCACGCACG
OUT192_Bre_1   TTAGACTCTTAGTGCAGCGTGACTCTCTTCCTGTCTCACACATACCCTCTG--------------------------------------------CTCTCTCTCTCTCTCTCTCT------------------CACGCACGCACG
OUT192_Bre_2   TTAGACTCTTAGTGCAGCGTGACTCTCTTCCTGTCTCACACATACCCTCTG--------------------------------------------CTCTCTCTCTCTCTCTCTCT------------------CACGCACGCACG
OUT194_Bre_1   TTAGACTCTTAGTGCAGCGTGACTCTCTTCCTGTCTCACACATACCCTCTG--------------------------------------------CTCTCTCTCTCTCTCTCTCT------------------CACGCACGCACG
OUT194_Bre_2   TTAGACTCTTAGTGCAGCGTGACTCTCTTCCTGTCTCACACATACCCTCTG--------------------------------------------CTCTCTCTCTCTCTCTCTCT------------------CACGCACGCACG
OUT198_Bre_1   TTAGACTCTTAGTGCAGCGTGACTCTCTTCCTGTCTCACACATACCCTCTG--------------------------------------------CTCTCTCTCTCTCTCTCTCT------------------CACGCACGCACG
OUT198_Bre_2   TTAGACTCTTAGTGCAGCGTGACTCTCTTCCTGTCTCACACATACCCTCTG------------------------------------------CTCTCTCTCTCTCTCTCTCTCT------------------CACGCACGCACG
OUT204_Bre_1   TTAGACTCTTAGTGCAGCGTGACTCTCTTCCTGTCTCACACATACCCTCTG--------------------------------------------CTCTCTCTCTCTCTCTCTCT------------------CACGCACGCACG
OUT204_Bre_2   TTAGACTCTTAGTGCAGCGTGACTCTCTTCCTGTCTCACACATACCTTCTG--------------------------------------------CTCTCTCTCTCTCTCTCTCT------------------CACGCACGCACG
OUT213_Bre_1   TTAGACTCTTAGTGCAGCGTGACTCTCTTCCTGTCTCACACATACCCTCTG--------------------------------------------CTCTCTCTCTCTCTCTCTCT------------------CACGCACGCACG
OUT213_Bre_2   TTAGACTCTTAGTGCAGCGTGACTCTCTTCCTGTCTCACACATACCCTCTG--------------------------------------------CTCTCTCTCTCTCTCTCTCT------------------CACGCACGCACG

TACACGCACACGCACA------------------------TATGGAGTTTGTCTTTTTAATTTCATTTTC
TACACGCACACGCACACGCACA------------------TATGGAGTTTGTCTTTTTAATTTCATTTTC
TACACGCACACGCACACGCACA------------------TATGGAGTTTGTCTTTTTAATTTCATTTTC
TACACGCACACGCACACGCACA------------------TATGGAGTTTGTCTTTTTAATTTCATTTTC
TACACGCACACGCACA------------------------TATGGAGTTTGTCTTTTTAATTTCATTTTC
TACACGCACACGCACACGCACA------------------TATGGAGTTTGTCTTTTTAATTTCATTTTC
TACACGCACACGCACACGCACA------------------TATGGAGTTTGTCTTTTTAATTTCATTTTC
TACACGCACACGCACACGCACA------------------TATGGAGTTTGTCTTTTTAATTTCATTTTC
TACACGCACACGCACACGCACA------------------TATGGAGTTTGTCTTTTTAATTTCATTTTC
TACACGCACACGCACACGCACA------------------TATGGAGTTTGTCTTTTTAATTTCATTTTC
TACACGCACACGCACACGCACA------------------TATGGAGTTTGTCTTTTTAATTTCATTTTC
TACACGCACACGCACACGCACA------------------TATGGAGTTTGTCTTTTTAATTTCATTTTC
TACACGCACACGCACACGCACA------------------TATGGAGTTTGTCTTTTTAATTTCATTTTC
TACACGCACACGCACACGCACA------------------TATGGAGTTTGTCTTTTTAATTTCATTTTC
TACACGCACACGCACACGCACA------------------TATGGAGTTTGTCTTTTTAATTTCATTTTC
TACACGCACACGCACACGCACA------------------TATGGAGTTTGTCTTTTTAATTTCATTTTC
TACACGCACACGCACACGCACA------------------TATGGAGTTTGTCTTTTTAATTTCATTTTC
TACACGCACACGCACACGCACA------------------TATGGAGTTTGTCTTTTTAATTTCATTTTC
TACACGCACACGCACACGCACA------------------TATGGAGTTTGTCTTTTTAATTTCATTTTC
TACACGCACACGCACACGCACA------------------TATGGAGTTTGTCTTTTTAATTTCATTTTC
TACACGCACACGCACACGCACA------------------TATGGAGTTTGTCTTTTTAATTTCATTTTC
TACACGCACACGCACACGCACA------------------TATGGAGTTTGTCTTTTTAATTTCATTTTC
TACACGCACACGCACACGCACA------------------TATGGAGTTTGTCTTTTTAATTTCATTTTC
TACACGCACACGCACACGCACACGCACA------------TATGGAGTTTGTCTTTTTAATTTCATTTTC
```

```
TACACGCACACGCACACGCACA------------------TATGGAGTTTGTCTTTTTAATTTCATTTTC
TACACGCACACGCACACGCACA------------------TATGGAGTTTGTCTTTTTAATTTCATTTTC
TACACGCACACGCACACGCACA------------------TATGGAGTTTGTCTTTTTAATTTCATTTTC
TACACGCACACGCACACGCACA------------------TATGGAGTTTGTCTTTTTAATTTCATTTTC
TACACGCACA------------------------------TATGGAGTTTGTCTTTTTAATTTCATTTTC
TACACGCACACGCACACGCACA------------------TATGGAGTTTGTCTTTTTAATTTCATTTTC
TACACGCACACGCACACGCACA------------------TATGGAGTTTGTCTTTTTAATTTCATTTTC
TACACGCACACGCACA------------------------TATGGAGTTTGTCTTTTTAATTTCATTTTC
TACACGCACACGCACACGCACA------------------TATGGAGTTTGTCTTTTTAATTTCATTTTC
TACACGCACACGCACACGCACA------------------TATGGAGTTTGTCTTTTTAATTTCATTTTC
TACACGCACACGCACACGCACA------------------TATGGAGTTTGTCTTTTTAATTTCATTTTC
TACACGCACACGCACACGCACA------------------TATGGAGTTTGTCTTTTTAATTTCATTTTC
TACACGCACACGCACACGCACA------------------TATGGAGTTTGTCTTTTTAATTTCATTTTC
TACACGCACACGCACACGCACA------------------TATGGAGTTTGTCTTTTTAATTTCATTTTC
TACACGCACACGCACACGCACA------------------TATGGAGTTTGTCTTTTTAATTTCATTTTC
TACACGCACACGCACACGCACA------------------TATGGAGTTTGTCTTTTTAATTTCATTTTC
TACACGCACACGCACACGCACA------------------TATGGAGTTTGTCTTTTTAATTTCATTTTC
TACACGCACA------------------------------TATGGAGTTTGTCTTTTTAATTTCATTTTC
TACACGCACACGCACACGCACA------------------TATGGAGTTTGTCTTTTTAATTTCATTTTC
TACACGCACACGCACACGCACA------------------TATGGAGTTTGTCTTTTTAATTTCATTTTC
TACACGCACACGCACACGCACA------------------TATGGAGTTTGTCTTTTTAATTTCATTTTC
TACACGCACACGCACACGCACA------------------TATGGAGTTTGTCTTTTTAATTTCATTTTC
TACACGCACA------------------------------TATGGAGTTTGTCTTTTTAATTTCATTTTC
TACACGCACACGCACACGCACA------------------TATGGAGTTTGTCTTTTTAATTTCATTTTC
TACACGCACACGCACACGCACACGCACA------------TATGGAGTTTGTCTTTTTAATTTCATTTTC
TACACGCACACGCACACGCACACGCACA------------TATGGAGTTTGTCTTTTTAATTTCATTTTC
TACACGCACACGCACACGCACA------------------TATGGAGTTTGTCTTTTTAATTTCATTTTC
TACACGCACACGCACACGCACA------------------TATGGAGTTTGTCTTTTTAATTTCATTTTC
TACACGCACACGCACACGCACA------------------TATGGAGTTTGTCTTTTTAATTTCATTTTC
TACACGCACACGCACACGCACA------------------TATGGAGTTTGTCTTTTTAATTTCATTTTC
TACACGCACACGCACACGCACA------------------TATGGAGTTTGTCTTTTTAATTTCATTTTC
TACACGCACACGCACACGCACA------------------TATGGAGTTTGTCTTTTTAATTTCATTTTC
TACACGCACACGCACACGCACA------------------TATGGAGTTTGTCTTTTTAATTTCATTTTC
TACACGCACACGCACACGCACA------------------TATGGAGTTTGTCTTTTTAATTTCATTTTC
TACACGCACACGCACACGCACA------------------TATGGAGTTTGTCTTTTTAATTTCATTTTC
TACACGCACACGCACACGCACACGCACA------------TATGGAGTTTGTCTTTTTAATTTCATTTTC
TACACGCACACGCACACGCACA------------------TATGGAGTTTGTCTTTTTAATTTCATTTTC
TACACGCACACGCACACGCACA------------------TATGGAGTTTGTCTTTTTAATTTCATTTTC
TACACGCACACGCACA------------TGCACATGCACATATGGAGTTTGTCTTTTTAATTTCATTTTC
TACACGCACACGCACACGCACA------------------TATGGAGTTTGTCTTTTTAATTTCATTTTC
TACACGCACACGCACA------------TGCACATGCACATATGGAGTTTGTCTTTTTAATTTCATTTTC
TACACGCACACGCACA------------TGCACATGCACATATGGAGTTTGTCTTTTTAATTTCATTTTC
TACACGCACACGCACACGCACA------------------TATGGAGTTTGTCTTTTTAATTTCATTTTC
TACACGCACACGCACACGCACA------------------TATGGAGTTTGTCTTTTTAATTTCATTTTC
TACACGCACACGCACA------------TGCACATGCACATATGGAGTTTGTCTTTTTAATTTCATTTTC
TACACGCACACGCACA------------TGCACATGCACATATGGAGTTTGTCTTTTTAATTTCATTTTC
TACACGCACACGCACA------------TGCACATGCACATATGGAGTTTGTCTTTTTAATTTCATTTTC
TACACGCACACGCACA------------TGCACATGCACATATGGAGTTTGTCTTTTTAATTTCATTTTC
TACACGCACACGCACA------------TGCACATGCACATATGGAGTTTGTCTTTTTAATTTCATTTTC
TACACGCACACGCACA------------TGCACATGCACATATGGAGTTTGTCTTTTTAATTTCATTTTC
TACACGCACACGCACA------------TGCACATGCACATATGGAGTTTGTCTTTTTAATTTCATTTTC
TACACGCACACGCACA------------TGCACATGCACATATGGAGTTTGTCTTTTTAATTTCATTTTC
TACACGCACACGCACA------------TGCACATGCACATATGGAGTTTGTCTTTTTAATTTCATTTTC
TACACGCACACGCACA------------TGCACATGCACATATGGAGTTTGTCTTTTTAATTTCATTTTC
TACACGCACACGCACA------------TGCACATGCACATATGGAGTTTGTCTTTTTAATTTCATTTTC
TACACGCACACGCACA------------TGCACATGCACATATGGAGTTTGTCTTTTTAATTTCATTTTC
TACACGCACACGCACA------------TGCACATGCACATATGGAGTTTGTCTTTTTAATTTCATTTTC
```

```
TACACGCACACGCACA------------TGCACATGCACATATGGAGTTTGTCTTTTTAATTTCATTTTC
TACACGCACACGCACACGCACA------------------TATGGAGTTTGTCTTTTTAATTTCATTTTC
TACACGCACACGCACACGCACA------------------TATGGAGTTTGTCTTTTTAATTTCATTTTC
TACACGCACACGCACACGCACA------------------TATGGAGTTTGTCTTTTTAATTTCATTTTC
TACACGCACACGCACACGCACA------------------TATGGAGTTTGTCTTTTTAATTTCATTTTC
TACACGCACACGCACA------------TGCACATGCACATATGGAGTTTGTCTTTTTAATTTCATTTTC
TACACGCACACGCACA------------TGCACATGCACATATGGAGTTTGTCTTTTTAATTTCATTTTC
TACACGCACACGCACA------------TGCACATGCACATATGGAGTTTGTCTTTTTAATTTCATTTTC
TACACGCACACGCACA------------TGCACATGCACATATGGAGTTTGTCTTTTTAATTTCATTTTC
TACACGCACACGCACA------------TGCACATGCACATATGGAGTTTGTCTTTTTAATTTCATTTTC
TACACGCACACGCACA------------TGCACATGCACATATGGAGTTTGTCTTTTTAATTTCATTTTC
TACACGCACACGCACA------------TGCACATGCACATATGGAGTTTGTCTTTTTAATTTCATTTTC
TACACGCACACGCACA------------TGCACATGCACATATGGAGTTTGTCTTTTTAATTTCATTTTC
TACACGCACACGCACA------------TGCACATGCACATATGGAGTTTGTCTTTTTAATTTCATTTTC
TACACGCACACGCACA------------TGCACATGCACATATGGAGTTTGTCTTTTTAATTTCATTTTC
```

QrZAG30

```
BUE020Qp_171   CTGAACCCTTATAAAATGACATTGCTTTTTAAATTAAAATATACACGAGAGAGAGAGAGA--------------------------------------------------------AAAGCAAAAGAAAACATGATGTTACTTTA
BUE023Qr_197   CTGAACCCTTATAAAATGACATTGCTTTTTAAATTAAAATATACACGAGAGAGAGAGAGAGAGAGAGAGAGAGAGAGAGAGAGAGAGAGAGA------------------------------AAAGCAAAAGAAAACATGATGTTGCTTTA
BUE023Qr_257   CTGAACCCTTATAAAATGACATTGCTTTTTAAATTAAAATATACACGAGAGAGAGAGAGAGAGAGAGAGAGAGAGAGAGAGAGAGAGAGAGA--------------------------------AGCAAAAGAAAACGTGATGTTACTTTA
BUE028Qr_206_1 CTGAACCCTTATAAAATGACATTGCTTTTTAAATTAAAATATACAAGAGAGAGAGAGAGAGAGAGAGAGAGAGAGAGAGAGAGAGAGAGAGAGAGAGAGAGAGA--------------------AAAGCAAAAGAAAACATGATGCTACTTTA
BUE028Qr_206_2 CTGAACCCTTATAAAATGACATTGCTTTTTAAATTAAAATATACAAGAGAGAGAGAGAGAGAGAGAGAGAGAGAGAGAGAGAGAGAGAGAGAGAGAGAGAGAGA--------------------AAAGCAAAAGAAAACATGATGCTACTTTA
BUE031Qr_224_1 CTGAACCCTTATAAAATGACATTGCTTTTTAAATTAAAATATACAAGAGAGAGAGAGAGAGAGAGAGAGAGAGAGAGAGAGAGAGAGAGAGAGAGAGAGAGAGAGAGAGAGAGAGAGAGAGAGA--AGCAAAAGAAAACATGATGTTACTTTA
BUE031Qr_224_2 CTGAACCCTTATAAAATGACATTGCTTTTTAAATTAAAATATACAAGAGAGAGAGAGAGAGAGAGAGAGAGAGAGAGAGAGAGAGAGAGAGAGAGAGAGAGAGAGAGAGAGAGAGAGAGAGAGA--AGCAAAAGAAAACATGATGTTACTTTA
BUE034Qr_201_1 CTGAACCCTTATAAAATGACATTGCTTTTTAAATTAAAATATACACGAGAGAGAGAGAGA----------------------------------------------------------AGCAAAAGAAAACGTGATGTTACTTTA
BUE034Qr_201_2 CTGAACCCTTATAAAATGACATTGCTTTTTAAATTAAAATATACACGAGAGAGAGAGAGA----------------------------------------------------------AGCAAAAGAAAACGTGATGTTACTTTA
BUE041Qr_208_1 CTGAACCCTTATAAAATGACATTGCTTTTTAAATTAAAATATACAAGAGAGAGAGAGAGAGAGAGAGAGAGAGAGAGAGAGAGAGAGAGAGAGAGAGAGAGAGAGAGA------------------AAAGCAAAAGAAAACATGATGTTACTTTA
BUE041Qr_208_2 CTGAACCCTTATAAAATGACATTGCTTTTTAAATTAAAATATACAAGAGAGAGAGAGAGAGAGAGAGAGAGAGAGAGAGAGAGAGAGAGAGAGAGAGAGAGAGAGAGA------------------AAAGCAAAAGAAAACATGATGTTACTTTA
BUE049Qr_210_1 CTGAACCCTTATAAAATGACATTGCTTTTTAAATTAAAATATACAAGAGAGAGAGAGAGAGAGAGAGAGAGAGAGAGAGAGAGAGAGAGAGAGAGAGAGAGAGAGAGAGAGAGA----------------AGCAAAAGAAAACATGATGTTACTTTA
BUE049Qr_210_2 CTGAACCCTTATAAAATGACATTGCTTTTTAAATTAAAATATACAAGAGAGAGAGAGAGAGAGAGAGAGAGAGAGAGAGAGAGAGAGAGAGAGAGAGAGAGAGAGAGAGAGAGA----------------AGCAAAAGAAAACATGATGTTACTTTA
BUE069Qr_201_1 CTGAACCCTTATAAAATGACATTGCTTTTTAAATTAAAATATACACGAGAGAGAGAGAGA----------------------------------------------------------AGCAAAAGAAAACGTGATGTTACTTTA
BUE069Qr_201_2 CTGAACCCTTATAAAATGACATTGCTTTTTAAATTAAAATATACACGAGAGAGAGAGAGA----------------------------------------------------------AGCAAAAGAAAACGTGATGTTACTTTA
BUE075Qr_220   CTGAACCCTTATAAAATGACATTGCTTTTTAAATTAAAATATACACGAGAGAGAGAGAGAGAGAGAGAGAGAGAGAGAGAGAGAGAGAGAGAGAGAGAGAGAGAGAGAGAGAGAGAGAGAGA------AAAGCAAAAGAAAACATGATGTTACTTTA
BUE080uncl_171 CTGAACCCTTATAAAATGACATTGCTTTTTAAATTAAAATATACACGAGAGAGAGAGAGA--------------------------------------------------------AAAGCAAAAGAAAACATGATGTTACTTTA
BUE080uncl_227 CTGAACCCTTATAAAATGACATTTCTTTTTAAATTAAAATATACACGAGAGAGAGAGAGAGAGAGAGAGAGAGAGAGAGAGAGAGAGAGAGA--------------------------------AGCAAAAGAAAACGTGATGTTACTTTA
BUE081Qr_199_1 CTGAACCCTTATAAAATGACATTGCTTTTTAAATTAAAATATACACGAGAGAGAGAGAGAGAGAGAGAGAGAGAGAGAGAGAGAGAGAGAGAGAGAGA----------------------------AGCAAAAGAAAACATGATGTTACTTTA
BUE081Qr_199_2 CTGAACCCTTATAAAATGACATTGCTTTTTAAATTAAAATATACACGAGAGAGAGAGAGAGAGAGAGAGAGAGAGAGAGAGAGAGAGAGAGAGAGAGA----------------------------AGCAAAAGAAAACATGATGTTACTTTA
BUE099Qp_197   CTGAACCCTTATAAAATGACATTGCTTTTTAAATTAAAATATACAAGAGAGAGAGAGAGAGAGAGAGAGAGAGAGAGAGAGAGAGAGAGAGA------------------------------AAAGCAAAAGGAAACATGATGTTACTTTA
BUE099Qp_197   CTGAACCCTTATAAAATGACATTGCTTTTTAAATTAAAATATACAAGAGAGAGAGAGAGAGAGAGAGAGAGAGAGAGAGAGAGAGAGAGAGA------------------------------AAAGCAAAAGGAAACATGATGTTACTTTA
BUE104Qp_171   CTGAACCCTTATAAAATGACATTGCTTTTTAAATTAAAATATACACGAGAGAGAGAGAGA--------------------------------------------------------AAAGCAAAAGAAAACATGATGTTACTTTA
BUE104Qp_209   CTGAACCCTTATAAAATGACATTGCTTTTTAAATTAAAATATACACGAGAGAGAGAGAGAGAGAGAGA--------------------------------------------------AGCAAAAGAAAACGTGATGTTACTTTA
BUE116Qr_187   CTGAACCCTTATAAAATGACATTGCTTTTTAAATTAAAATATACACGAGAGAGAGAGAGAGAGAGAGAGAGAGAGAGA----------------------------------------AGCAAAAGAAAACATGATGTTACTTTA
BUE116Qr_238   CTGAACCCTTATAAAATGACATTGCTTTTTAAATTAAAATATACACGAGAGAGAGAGAGAGAGAGAGAGAGAGAGAGAGAGAGAGAGAGAGAGAGAGAGAGAGAGA--------------------AGCAAAAGAAAACGTGATGTTACTTTA
BUE141Qr_212   CTGAACCCTTATAAAATGACATTGCTTTTTAAATTAAAATATACAAGAGAGAGAGAGAGAGAGAGAGAGAGAGAGAGAGAGAGAGAGAGAGAGAGAGAGAGAGAGAGAGAGAGAGA--------------AGCAAAAGAAAACATGATGTTACTTTA
BUE141Qr_251   CTGAACCCTTATAAAATGACATTGCTTTTTAAATTAAAATATACACGAGAGAGAGAGAGAGAGAGAGAGAGAGAGAGAGAGAGAGAGAGAGAGAGAGAGAGAGAGAGAGAGAGAGAGAGAGA--------AGCAAAAGAAAACGTGATGTTACTTTA
BUE150Qp_201   CTGAACCCTTATAAAATGACATTGCTTTTTAAATTAAAATATACACGAGAGAGAGAGAGA----------------------------------------------------------AGCAAAAGAAAACGTGATGTTACTTTA
BUE150Qp_224   CTGAACCCTTATAAAATGACATTGCTTTTTAAATTAAAATATACACGAGAGAGAGAGAGAGAGAGAGAGAGAGAGAGAGAGAGA------------------------------------AGCAAAAGAAAACGTGATGTTACTTTA
BUE161Qr_219   CTGAACCCTTATAAAATGACATTGCTTTTTAAATTAAAATATACACGAGAGAGAGAGAGAGAGAGAGAGAGAGAGAGA----------------------------------------AGCAAAAGAAAACGTGATGTTACTTTA
BUE161Qr_227   CTGAACCCTTATAAAATGACATTTCTTTTTAAATTAAAATATACACGAGAGAGAGAGAGAGAGAGAGAGAGAGAGAGAGAGAGAGAGAGAGA--------------------------------AGCAAAAGAAAACGTGATGTTACTTTA
BUE171Qp_204   CTGAACCCTTATAAAATGACATTGCTTTTTAAATTAAAATATACAAGAGAGAGAGAGAGAGAGAGAGAGAGAGAGAGAGAGAGAGAGAGAGAGAGAGAGA----------------------AAAGCAAAAGAAAACATGATGTTACTTTA
BUE171Qp_212   CTGAACCCTTATAAAATGACATTGCTTTTTAAATTAAAATATACAAGAGAGAGAGAGAGAGAGAGAGAGAGAGAGAGAGAGAGAGAGAGAGAGAGAGAGAGAGAGAGAGAGA--------------AAAGCAAAAGAAAACATGATGTTACTTTA
BUE183Qr_225   CTGAACCCTTATAAAATGACATTGCTTTTTAAATTAAAATATACACGAGAGAGAGAGAGAGAGAGAGAGAGAGAGAGAGAGAGAGAGA----------------------------------AGCAAAAGAAAACGTGATGTTACTTTA
BUE183Qr_238   CTGAACCCTTATAAAATGACATTGCTTTTTAAATTAAAATATACACGAGAGAGAGAGAGAGAGAGAGAGAGAGAGAGAGAGAGAGAGAGAGAGAGAGAGAGAGAGA--------------------AGCAAAAGAAAACGTGATGTTACTTTA
BUE215uncl_183 CTGAACCCTTATAAAATGACATTGCTTTTTAAATTAAAATATACACGAGAGAGAGAGAGAGAGAGAGAGAGAGAGAGAGAGAGAGAGAGAGAGAGAGAGA------------------------AGCAAAAGAAAACGTGATGTTACTTTA
BUE325Qr_211_1 CTGAACCCTTATAAAATGACATTGCTTTTTAAATTAAAATATACACGAGAGAGAGAGAGAGAGAGAGAGA------------------------------------------------AGCAAAAGAAAACGTGATGTTACTTTA
```

```
BUE325Qr_211_2 CTGAACCCTTATAAAATGACATTGCTTTTTAAATTAAAATATACACGAGAGAGAGAGAGAGAGAGAGAGA------------------------------------------------AGCAAAAGAAAACGTGATGTTACTTTA
BUE345Qr_202_1 CTGAACCCTTATAAAATGACATTGCTTTTTAAATTAAAATATACAAGAGAGAGAGAGAGAGAGAGAGAGAGAGAGAGAGAGAGAGAGAGAGAGA------------------------AAAGCAAAAGAAAACATGATGTTACTTTA
BUE345Qr_202_2 CTGAACCCTTATAAAATGACATTGCTTTTTAAATTAAAATATACAAGAGAGAGAGAGAGAGAGAGAGAGAGAGAGAGAGAGAGAGAGAGAGAGA------------------------AAAGCAAAAGAAAACATGATGTTACTTTA
BUE361Qr_232_1 CTGAACCCTTATAAAATGACATTGCTTTTTAAATTAAAATATACACGAGAGAGAGAGAGAGAGAGAGAGAGAGAGAGAGAGAGAGAGAGAGA----------------------------AGCAAAAGAAAACGTGATGTTACTTTA
BUE361Qr_232_2 CTGAACCCTTATAAAATGACATTGCTTTTTAAATTAAAATATACACGAGAGAGAGAGAGAGAGAGAGAGAGAGAGAGAGAGAGAGAGAGAGA----------------------------AGCAAAAGAAAACGTGATGTTACTTTA
BUE393Qr_171   CTGAACCCTTATAAAATGACATTGCTTTTTAAATTAAAATATACACGAGAGAGAGAGAGAGA--------------------------------------------------------AAAGCAAAAGAAAACATGATGTTACTTTA
BUE393Qr_202   CTGAACCCTTATAAAATGACATTGCTTTTTAAATTAAAATATACAAGAGAGAGAGAGAGAGAGAGAGAGAGAGAGAGAGAGAGAGAGAGAGAGA------------------------AAAGCAAAAGAAAACATGATGTTACTTTA
BUE571Qr_257_1 CTGAACCCTTATAAAATGACATTGCTTTTTAAATTAAAATATACACGAGAGAGAGAGAGAGAGAGAGAGAGAGAGAGAGAGAGAGA--------------------------------AGCAAAAGAAAACGTGATGTTACTTTA
BUE571Qr_257_2 CTGAACCCTTATAAAATGACATTGCTTTTTAAATTAAAATATACACGAGAGAGAGAGAGAGAGAGAGAGAGAGAGAGAGAGAGAGA--------------------------------AGCAAAAGAAAACGTGATGTTACTTTA

TCCATAATGCTCATTTGTGGG------------------------------------------------------------------CAATAGCCACATATAGGGATGTG
TCCATAATGCTCATTTGTGGG------------------------------------------------------------------CAATAGCCACGTATAGGGATGTG
TCCATAATGCTCATTTGTGGGATGTTTTTTTTTTTTTGATAAAAAAAACAACACAGAGAACTTT---CATTAAT-CTGATTTGTGGGCAATAGCCACATATAGGGGTGTG
TCCATAATGCTCATTTGTGGG------------------------------------------------------------------CAATAGCCACGTATAGGGATGTG
TCCATAATGCTCATTTGTGGG------------------------------------------------------------------CAATAGCCACGTATAGGGATGTG
TCCATAATGCTCATTTGTGGG------------------------------------------------------------------CAATAGCCACGTATAGGGATGTG
TCCATAATGCTCATTTGTGGG------------------------------------------------------------------CAATAGCCACGTATAGGGATGTG
TCCATAATGCTCATTTGTGGGATGTT---------------------------------ACTTTATCCA-TAATGCTCATTTGTGGGCAATAGCCACATATAGGGGTGTG
TCCATAATGCTCATTTGTGGGATGTT---------------------------------ACTTTATCCA-TAATGCTCATTTGTGGGCAATAGCCACATATAGGGGTGTG
TCCATAATGCTCATTTGTGGG------------------------------------------------------------------CAATAGCCACGTATAGGGATGTG
TCCATAATGCTCATTTGTGGG------------------------------------------------------------------CAATAGCCACGTATAGGGATGTG
TCCATAATGCTCATTTGTGGG------------------------------------------------------------------CAATAGCCACGTATAGGGATGTG
TCCATAATGCTCATTTGTGGG------------------------------------------------------------------CAATAGCCACGTATAGGGATGTG
TCCATAATGCTCATTTGTGGGATGTT---------------------------------ACTTTATCCA-TAATGCTCATTTGTGGGCAATAGCCACATATAGGGATGTG
TCCATAATGCTCATTTGTGGGATGTT---------------------------------ACTTTATCCA-TAATGCTCATTTGTGGGCAATAGCCACATATAGGGATGTG
TCCATAATGCTCATTTGTGGG------------------------------------------------------------------CAATAGCCACGTATAGG-ATGTG
TCCATAATGCTCATTTGTGGG------------------------------------------------------------------CAATAGCCACATACAGGGATGTG
TCCATAATGCTCATTTGTGGGATGTT---------------------------------ACTTTATCCA-TAATGCTCATTTGTGGGCAATAGCCACATATAGGGATGTG
TCCATAATGCTCATTTGTGGG------------------------------------------------------------------CAATAGCCACGTATAGGGATGTG
TCCATAATGCTCATTTGTGGG------------------------------------------------------------------CAATAGCCACGTATAGGGATGTG
TCCATAATGCTCATTTGTGGG------------------------------------------------------------------CAATAGCCACGTATAGGGATGTG
TCCATAATGCTCATTTGTGGG------------------------------------------------------------------CAATAGCCACGTATAGGGATGTG
TCCATAATGCTCATTTGTGGG------------------------------------------------------------------CAATAGCCACATATAGGGATGTG
TCCATAATGCTCATTTGTGGGATGTT---------------------------------ACTTTATCCA-TAATGCTTATTTGTGGGCAATAGCCACATATAGGGATGTG
TCCATAATGCTCATTTGTGGG------------------------------------------------------------------CAATAGCCACGTATAGGGATGTG
TCCATAATGCCCATTTGTGGGATGTT---------------------------------ACTTTATCCA-TAATGCTCATTTGTGGGCAATAGCCACATATAGGGATGTG
TCCATAATGCTCATTTGTGGG------------------------------------------------------------------CAATAGCCACGTATAGGGATGTG
TCCATAATGCCCATTTGTGGGATGTT---------------------------------ACTTTATCCA-TAATGCTCATTTGTGGGCAATAGCCACATATAGGGATGTG
TCCATAATGCTCATTTGTGGGATGTT---------------------------------ACTTTATCCA-TAATGCTCATTTGTGGGCAATAGCCACATATAGGGATGTG
TCCATAATGCTCATTTGTGGGATGTT---------------------------------ACTTTATCCA-TAATGCTCATTTGTGGGCAATAGCCACATATAGGGATGTG
TCCATAATGCTCATTTGTGGGATGTT---------------------------------ACTTTATCCA-TAATGCTCATTTGTGGGCAATAGCCACATATAGGGATGTG
TCCATAATGCTCATTTGTGGGATGTT---------------------------------ACTTTATCCA-TAATGCTCATTTGTGGGCAATAGCCACATATAGGGATGTG
TCCATAATGCTCATTTGTGGG------------------------------------------------------------------CAATAGCCACGTATAGGGATGTG
TCCATAATGCTCATTTGTGGG------------------------------------------------------------------CAATAGCCACGTATAGGGATGTG
TCCATAATGCTCATTTGTGGGATGTT---------------------------------ACTTTATCCA-TAATGCTCATTTGTGGGCAATAGCCACATATAGGGGTGTG
TCCATAATGCTCATTTGTGGGATGTT---------------------------------ACTTTATCCA-TAATGCTCATTTGTGGGCAATAGCCACATACAGGGGTGTG
TCCATAATGCTCATTTGTGGGATGTT---------------------------------ACTTTATCCA-TAATGCTCATTTGTGGGCAATAGCCACATATAGGGATGTG
TCCATAATGCTCATTTGTGGGATGTT---------------------------------ACTTTATCCA-TAATGCTCATTTGTGGGCAATAGCCACATATAGGGATGTG
TCCATAATGCTCATTTGTGGGATGTT---------------------------------ACTTTATCCA-TAATGCTCATTTGTGGGCAATAGCCACATATAGGGATGTG
TCCATAATGCTCATTTGTGGG------------------------------------------------------------------CAATAGCCACGTATAGGGATGTG
TCCATAATGCTCATTTGTGGG------------------------------------------------------------------CAATAGCCACGTATAGGGATGTG
TCCATAATGCTCATTTGTGGGATGTT---------------------------------ACTTTATCCA-TAATGCTCATTTGTGGGCAATAGCCACATATAGGGATGTG
TCCATAATGCTCATTTGTGGGATGTT---------------------------------ACTTTATCCA-TAATGCTCATTTGTGGGCAATAGCCACATATAGGGATGTG
TCCATAATGCTCATTTGTGGG------------------------------------------------------------------CAATAGCCACATATAGGGATGTG
TCTATAATGCTCATTTGTGGG------------------------------------------------------------------CAATAGCCACGTATAGGGATGTG
TCCATAATGCTCATTTGTGGGATGTTTTTTTTTTTTTGATAAAAAAAACAACACAGAGAACTTT---CATTAAT-CTGATTTGTGGGCAATAGCCACATATAGGGATGTG
TCCATAATGCTCATTTGTGGGATGTTTTTTTTTTTTTGATAAAAAAAACAACACAGAGAACTTT---CATTAAT-CTGATTTGTGGGCAATAGCCACATATAGGGATGTG
```
