# Supplementary material for: Always Look on Both Sides: Phylogenetic Information Conveyed by Simple Sequence Repeat Allele Sequences
Source: PLoS One. 2012 Jul 13;7(7):e40699. doi: 10.1371/journal.pone.0040699 (PMC3396589; doi:10.1371/journal.pone.0040699)
Supplement: Figure S2 — Z-statistics and summary of Mantel tests for the correlations of pairs of genetic distance matrices within species. Colouring (shading) indicates the level of significance of the test: green (light grey*), P>0.05; orange (grey*), 0.050.001; red (dark grey*), P<0.001. Identity of DNA fragment sections is reported: simple sequence repeat (SSR) variation; amplicon size variation, flanking region (FR) sequence variation, and amplicon sequence variation. Marker names are displayed above each panel and data sets refer to Citrus (C), Jacaranda (J) and Quercus (Q). Sequences with single nucleotide polymorphisms (SNPs) within the repeat were excluded from subsequent analyses because of the complexity to describe the mutation model of SSR repeats bracketing SNPs. (PDF) [file pone.0040699.s002.pdf]

### cAGG9 (Set C)

| | SSR | Amplicon size | FR sequence | Amplicon sequence |
|---|---|---|---|---|
| SSR | - | - | - | - |
| Amplicon size | 3381 | - | - | - |
| FR sequence | 438 | 1716 | - | - |
| Amplicon sequence | 3976 | 15234 | 2114 | - |

### Jc3A10 (Set J)

| | SSR | Amplicon size | FR sequence | Amplicon sequence |
|---|---|---|---|---|
| SSR | - | - | - | - |
| Amplicon size | 99287 | - | - | - |
| FR sequence | 4723 | 9135 | - | - |
| Amplicon sequence | 140905 | 221320 | 15362 | - |

### CCT01 (Set C)

| | SSR | Amplicon size | FR sequence | Amplicon sequence |
|---|---|---|---|---|
| SSR | - | - | - | - |
| Amplicon size | - | - | - | - |
| FR sequence | - | 1923 | - | - |
| Amplicon sequence | - | 8139 | 4190 | - |

### Jc3F4 (Set J)

| | SSR | Amplicon size | FR sequence | Amplicon sequence |
|---|---|---|---|---|
| SSR | - | - | - | - |
| Amplicon size | 20396 | - | - | - |
| FR sequence | 28857 | 63307 | - | - |
| Amplicon sequence | 208873 | 437446 | 79496 | - |

### GT03 (Set C)

| | SSR | Amplicon size | FR sequence | Amplicon sequence |
|---|---|---|---|---|
| SSR | - | - | - | - |
| Amplicon size | - | - | - | - |
| FR sequence | - | 6670 | - | - |
| Amplicon sequence | - | 32022 | 10612 | - |

### Jc3H10 (Set J)

| | SSR | Amplicon size | FR sequence | Amplicon sequence |
|---|---|---|---|---|
| SSR | - | - | - | - |
| Amplicon size | 536254 | - | - | - |
| FR sequence | 36187 | 78765 | - | - |
| Amplicon sequence | 71197314 | 2418089 | 326451 | - |

### QrZAG30 (Set Q)

| | SSR | Amplicon size | FR sequence | Amplicon sequence |
|---|---|---|---|---|
| SSR | - | - | - | - |
| Amplicon size | 131723 | - | - | - |
| FR sequence | 106440 | 474646 | - | - |
| Amplicon sequence | 232872 | 812074 | 863056 | - |
