# Supplementary material for: Always Look on Both Sides: Phylogenetic Information Conveyed by Simple Sequence Repeat Allele Sequences
Source: PLoS One. 2012 Jul 13;7(7):e40699. doi: 10.1371/journal.pone.0040699 (PMC3396589; doi:10.1371/journal.pone.0040699)
Supplement: Table S1 — List and details of the sampled sites for the Jacaranda (J) data set. (DOC) [file pone.0040699.s003.doc]

| Data Set | Country | Location | Latitude | Longitude |
| --- | --- | --- | --- | --- |
| Data J | French Guiana | Counami | 5.35°N | 52.18°E |
|  | French Guiana | Paracou | 5.25°N | 52.92°E |
|  | French Guiana | Saint-Laurent | 5.49°N | 54.04°E |
|  | Brazil | Tapajos | 3.07°S | 55.58°E |
