# Supplementary material for: Always Look on Both Sides: Phylogenetic Information Conveyed by Simple Sequence Repeat Allele Sequences
Source: PLoS One. 2012 Jul 13;7(7):e40699. doi: 10.1371/journal.pone.0040699 (PMC3396589; doi:10.1371/journal.pone.0040699)
Supplement: Table S2 — Description, amplification conditions and polymorphism of the nuclear simple sequence repeats (SSRs) analysed in Jacaranda copaia . (DOC) [file pone.0040699.s004.doc]

| Name | Ta (°C)^a^ | Repeat Motif | Size (bp)^b^ | Primer sequence | *n*^c^ | GenBank/EMBL accession number |
| --- | --- | --- | --- | --- | --- | --- |
| *Jc3A10* | 57°C | (GA)4(CT)8(CA)11 | 242 | F: AATTTCACAGCGGCTCCTC | 201 | JN661809 |
|  |  |  |  | R: CCCTTCTCTTTCTCGTGTCG |  |  |
| *Jc3F4* | 60°C | (GA)16 | 226 | F:CCAGGCAAAGCATCGTAAAT | 201 | JN661810 |
|  |  |  |  | R: GCACATTAATGGCGTCGTC |  |  |
| *Jc3H10* | 60°C | (TC)18 AT(AC)5 | 239 | F:CGTTCATGGTGTAGAACTTCAGA | 201 | JN661811 |
|  |  |  |  | R: CCTCAACCAAGACACAGCAA |  |  |

^a^ Annealing temperature

^b^ Total length of the amplicon sequence in base pairs

^c^ Total number of analysed samples
