# Supplementary material for: Always Look on Both Sides: Phylogenetic Information Conveyed by Simple Sequence Repeat Allele Sequences
Source: PLoS One. 2012 Jul 13;7(7):e40699. doi: 10.1371/journal.pone.0040699 (PMC3396589; doi:10.1371/journal.pone.0040699)
Supplement: Table S3 — List and details of alleles for the three data sets. Amplicon sizes, frequencies and number of associated haplotypes are indicated for each allele. (DOC) [file pone.0040699.s005.doc]

| SSR locus  (data set) | Amplicon size  (bp) | Allele frequency | Number of haplotype |
| --- | --- | --- | --- |
| cAGG9 (C) | 104 | 2 | 1 |
|  | 105 | 7 | 4 |
|  | 114 | 8 | 2 |
|  | 117 | 9 | 3 |
|  | 120 | 8 | 1 |
| CCT01 (C) | 158 | 7 | 3 |
|  | 161 | 12 | 8 |
|  | 164 | 8 | 6 |
|  | 167 | 7 | 3 |
| GT03 (C) | 151 | 2 | 1 |
|  | 153 | 5 | 3 |
|  | 167 | 9 | 2 |
|  | 171 | 10 | 4 |
|  | 173 | 10 | 2 |
| Jc3A10 (J) | 141 | 1 | 1 |
|  | 145 | 4 | 2 |
|  | 147 | 24 | 3 |
|  | 149 | 16 | 2 |
|  | 150 | 1 | 1 |
|  | 151 | 17 | 10 |
|  | 153 | 16 | 7 |
|  | 155 | 8 | 5 |
|  | 157 | 2 | 2 |
|  | 159 | 5 | 5 |
|  | 161 | 2 | 2 |
|  | 163 | 2 | 1 |
|  | 165 | 1 | 1 |
|  | 173 | 1 | 1 |
| Jc3F4 (J) | 112 | 1 | 1 |
|  | 113 | 4 | 2 |
|  | 114 | 18 | 3 |
|  | 116 | 2 | 2 |
|  | 117 | 21 | 1 |
|  | 118 | 4 | 1 |
|  | 119 | 1 | 1 |
|  | 120 | 10 | 3 |
|  | 121 | 6 | 1 |
|  | 122 | 8 | 3 |
|  | 123 | 8 | 2 |
|  | 124 | 1 | 1 |
|  | 125 | 3 | 1 |
|  | 127 | 1 | 1 |
|  | 128 | 1 | 1 |
|  | 129 | 1 | 1 |
|  | 130 | 2 | 2 |
|  | 132 | 1 | 1 |
|  | 134 | 1 | 1 |
|  | 136 | 3 | 1 |
|  | 140 | 3 | 1 |
|  | 142 | 1 | 1 |
| Jc3H10 (J) | 116 | 2 | 1 |
|  | 118 | 1 | 1 |
|  | 120 | 1 | 1 |
|  | 126 | 1 | 1 |
|  | 128 | 18 | 2 |
|  | 130 | 2 | 2 |
|  | 132 | 4 | 1 |
|  | 134 | 6 | 2 |
|  | 136 | 2 | 2 |
|  | 140 | 5 | 3 |
| Jc3H10 (J) | 141 | 20 | 2 |
|  | 143 | 7 | 1 |
|  | 144 | 2 | 2 |
|  | 146 | 2 | 2 |
|  | 148 | 4 | 3 |
|  | 150 | 5 | 2 |
|  | 152 | 2 | 2 |
|  | 154 | 1 | 1 |
|  | 156 | 1 | 1 |
|  | 158 | 3 | 2 |
|  | 160 | 2 | 1 |
|  | 162 | 1 | 1 |
|  | 166 | 1 | 1 |
|  | 168 | 2 | 2 |
|  | 170 | 1 | 1 |
| QrZAG30 (Q) | 133 | 4 | 2 |
|  | 149 | 1 | 1 |
|  | 159 | 3 | 2 |
|  | 161 | 2 | 1 |
|  | 163 | 5 | 2 |
|  | 165 | 3 | 2 |
|  | 167 | 1 | 1 |
|  | 169 | 2 | 1 |
|  | 171 | 3 | 2 |
|  | 173 | 4 | 2 |
|  | 175 | 2 | 2 |
|  | 181 | 1 | 1 |
|  | 182 | 1 | 1 |
|  | 185 | 1 | 1 |
|  | 187 | 3 | 2 |
|  | 189 | 2 | 1 |
|  | 193 | 2 | 1 |
|  | 197 | 1 | 1 |
|  | 201 | 2 | 2 |
|  | 213 | 1 | 1 |
|  | 219 | 3 | 2 |
